# Combinatorial targeting of the *miR-379/miR-410* cluster normalizes glucose and lipid homeostasis in male models of diabetes and obesity

**DOI:** 10.1038/s41467-026-77808-2

**Published:** 2026-09-21

**Authors:** Manuel Gil-Lozano, Estefanía Simoes, Moya Wu, Tobias Wiedemann, Yun Kwon, Revathi Sekar, Pauline Morigny, Maria Troullinaki, Juliane Merl-Pham, Michael Buettner, Annette Feuchtinger, Celine Jouffe, Adriano Maida, Daniela Hass, Daniel Samaga, Gretchen Wolff, Juliano Machado, Maude Giroud, Katarina Klepac, Carolyn L. Cummins, Marcos Ríos García, Peter Weber, Anja Zeigerer, N. Henriette Uhlenhaut, Julia Szendroedi, Matthias Blüher, Stephan Herzig

**Affiliations:** 1 Institute for Diabetes and Cancer, Helmholtz Munich, Neuherberg, Germany; 2 Joint Heidelberg-IDC Translational Diabetes Program, Inner Medicine 1, Heidelberg University Hospital, Heidelberg, Germany; 3 German Center for Diabetes Research (DZD), Neuherberg, Germany; 4 European Center for Angioscience (ECAS), Medical Faculty Mannheim, Heidelberg University, Mannheim, Germany; 5 Metabolomics and Proteomics Core, Helmholtz Munich, Neuherberg, Germany; 6 Metabolomics Core Technology Platform, University of Heidelberg, Heidelberg, Germany; 7 Core Facility Pathology and Tissue Analytics, Helmholtz Munich, Neuherberg, Germany; 8 Department of Pharmaceutical Sciences, University of Toronto, Toronto, Canada; 9 NeurObesity Group, Department of Physiology, CIMUS, University of Santiago de Compostela-Instituto de Investigación Sanitaria, Santiago de Compostela, Spain; 10 Institute of cancer and genomic science, University of Birmingham, Edgbaston, Birmingham, UK; 11 Institute for Diabetes and Endocrinology (IDE), Helmholtz Munich, Neuherberg, Germany; 12 Metabolic Programming, ZIEL Institute for Food and Health, TUM School of Life Sciences, Freising, Germany; 13 Department of Medicine I and Clinical Chemistry, University Hospital of Heidelberg, Heidelberg, Germany; 14 Institute for Metabolic, Obesity and Vascular Research, Helmholtz Munich, Leipzig, Germany; 15 Department of Medicine, University of Leipzig, Leipzig, Germany; 16 Chair Molecular Metabolic Control, Technical University Munich, Munich, Germany; 17 German Center for Cardiovascular Disease (DZHK), Munich, Germany

**Keywords:** Physiology, Molecular medicine, Diabetes complications, Mechanisms of disease

## Abstract

Non-coding RNAs from the *Dlk1-Dio3* locus are critical for the maturation of metabolic tissues in early stages of postnatal development; however, their role in the mature organs remains elusive. Herein, we show that microRNAs from the *miR-379/miR-410* cluster are robustly upregulated in livers of subjects with obesity and various mouse models of metabolic dysfunction. Adult-onset, combinatorial inhibition of this miRNA cluster by hepatocyte-specific expression of a decoy sequence reduces triglyceride, total and LDL cholesterol circulating levels, decreases basal glycemia and improves glucose tolerance and insulin sensitivity irrespective of sex. Consistent with the decoy-triggered enhancement of PI3K/mTOR signaling in these mice, hepatocytes expressing the combinatorial decoy show augmented mitochondrial mass and function. Notably, decoy therapy also ameliorates glucose and lipid homeostasis in both type 1 diabetic and diet-induced, type 2 pre-diabetic, obese male animals. Collectively, our results demonstrate that microRNAs from the *miR-379/miR-410* cluster are critical regulators of metabolic homeostasis in the mature liver. Given the preservation of miRNA dysfunction in human obesity, hepatocyte-specific combinatorial inhibition of a large miRNA cluster represents an approach towards multi-parameter improvements in diabetes and obesity.

## Abstract (web summary)

Hepatic-specific repression of the miR-379/miR-410 cluster in adult mice impacts hepatic insulin signalling and affects whole-body glucose and lipid metabolism, improving glycemic control and dyslipidemia in male models of obesity and diabetes.

## Introduction

Numerous non-coding RNAs, particularly microRNAs (miRNAs), have been implicated in the regulation of multiple biological processes over the past few years^1^. Most notably, miRNAs have been demonstrated not only to regulate metabolic homeostasis in the tissues they are expressed, but also to impact whole body energy homeostasis. Seminal studies in the field include the identification of pancreatic *miR-375* as the first miRNA able to affect whole body metabolism in mammals, by regulating insulin secretion and β-cell mass expansion^2,3^. Shortly after, hepatic *miR-122* was identified as the first miRNA with a role in liver metabolism, namely lipid homeostasis. Manipulation of this transcript by antisense oligonucleotides was associated with reductions in circulating cholesterol levels^4,5^. Another milestone was the identification of different miRNAs involved in the regulation of insulin signaling in the liver, including miR-103, miR-107, miR-802 and miR-143^6–8^. Accordingly, manipulation of such non-coding RNAs has been demonstrated to impact whole body glucose homeostasis. These and other studies exemplify the feasibility of miRNA-targeted drugs to ameliorate metabolic alterations in a variety of experimental disease models. However, none of the above miRNA-based therapeutic approaches targeting single miRNA species has reached clinical application yet.

The *Dlk1-Dio3* locus represents the largest miRNA cluster in eutherian mammals^9^; originally described as the mapping site of the callipyge mutation in sheep^10,11^. The locus is imprinted^12^; while the paternal chromosome encodes a few protein coding genes (*Dlk1*, *Rtl1* and *Dio3*), the maternal chromosome expresses a variety of non-coding RNAs, including long non-coding RNAs, small nucleolar RNAs and miRNAs^13^. Most miRNAs from the locus are grouped in two major clusters, the *miR-127/miR-136* cluster, located in the mid part of the locus, and the *miR-379/miR-410* cluster, on the distal end. Components of this latter cluster are cleaved from a common primary transcript known as l*ncMGC*^14^. This imprinted domain is highly conserved across different species, including sheep, dog, rat, mouse (on distal chromosome 12) and humans (chromosome 14q32). Maternally-expressed non-coding RNAs are critical for embryonic and early postnatal development. Thus, biallelic repression of these non-coding RNAs, by deletion on the maternal chromosome of the differentially methylated region (IG-DMR), responsible for the imprinted regulation of the locus, results in late-gestational lethality^15^. Related to this, a constitutive KO model of the larger, more distal cluster of miRNAs, also exhibits high postnatal lethality^16^. The high mortality rate was linked to severe hypoglycemia, indicating a potential role for these miRNAs in the regulation of hepatic glucose metabolism in the newborn. The high neonatal lethality makes this model not ideal to study glucose homeostasis in mature animals, since only the mice less sensitive to the action of these miRNAs, and those able to develop compensatory mechanisms, survive the weaning period. To circumvent this issue, alternative KO models carrying deletion of only a fraction of the miRNAs of the cluster have been generated^17,18^. Animals from these models are born in the expected Mendelian ratios and are viable, depicting no overt defects or abnormalities. Most notably, these KO models show reduced fat accumulation, along with improved dyslipidemia and insulin resistance when subjected to a high-fat diet^19,20^. These studies confirm our previous findings that hepatic *miR-379* controls circulating triglyceride levels by posttranscriptional regulation of hepatic very-low-density lipoprotein (VLDL) uptake transporters^21^; and overall support a role for these non-coding RNAs in the regulation of energy homeostasis. However, as constitutive knockouts, it is not possible to determine the major tissue responsible for the phenotype, which might be secondary to defects in adipocyte expansion^19^ or to muscular hypertrophy^17^.

Herein, we demonstrate that beyond *miR-379*, other miRNAs from the *miR-379/miR-410* cluster are consistently upregulated in livers of different models of hepatic insulin resistance and increased hepatic glucose output, including subjects with prediabetic obesity. We also describe an approach to repress the *miR-379/miR-410* cluster (the largest congregate of miRNAs in mammals) specifically in hepatocytes, by an AAV-delivered tough decoy (TuD), targeting distinct regions of two host transcripts of the cluster, *lncMGC* and *Mirg*. The combinatorial inhibition of those miRNAs promoted profound metabolic effects in adult mice on regular chow irrespectively of sex. Moreover, it also corrected the severe metabolic alterations in experimental models of both type 1 diabetes and diet-induced, pre-diabetic obesity. Together, these findings demonstrate that miRNAs from the *miR-379/miR-410* cluster participate in the regulation of hepatic metabolic homeostasis in adult mice, as well as the feasibility of combinatorial miRNA targeting as a therapeutic approach towards diabetes and obesity.

## Results

### *miR-379* and *miR-541* from the DLK1-DIO3 locus are upregulated in the liver of subjects with obesity and in mouse models of metabolic dysfunction

**Fig. 1:**
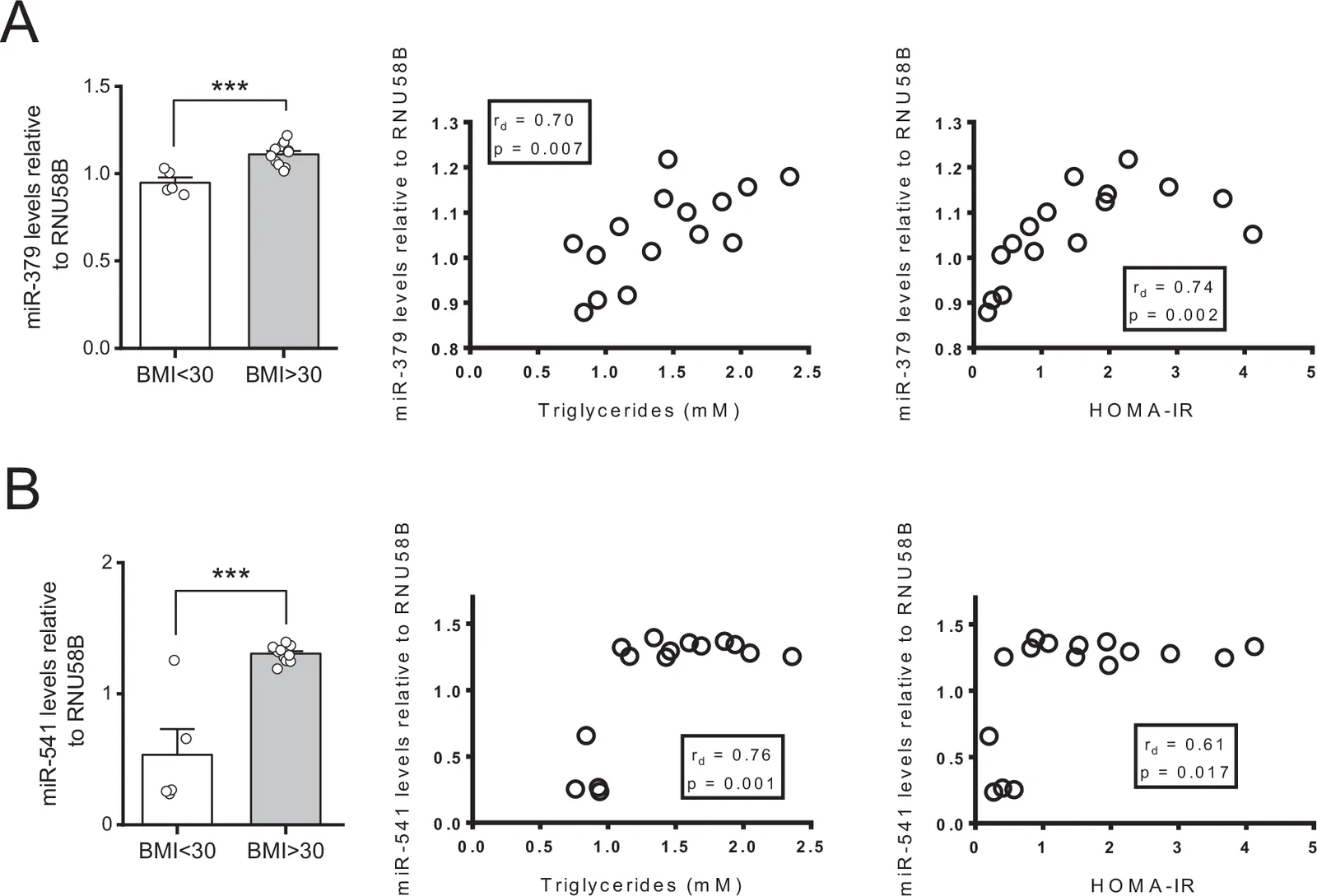
Upregulation of miR-379 and miR-541 in the liver of subjects with obesity. Levels of expression of *miR-379* (**A**) and *miR-541* (**B**) in the liver and distance correlation of these levels with circulating concentrations of triglycerides and HOMA-IR values in subjects with BMI < 30 (*n* = 5) vs. BMI > 30 (*n* = 11). Data are expressed as mean ± SEM; two-tailed Student’s *t* tests were used to compare the hepatic levels of expression of *miR-379* and *miR-541* between subjects with BMI < 30 and BMI > 30; ***P < 0.001 vs. control.

**Fig. 2:**
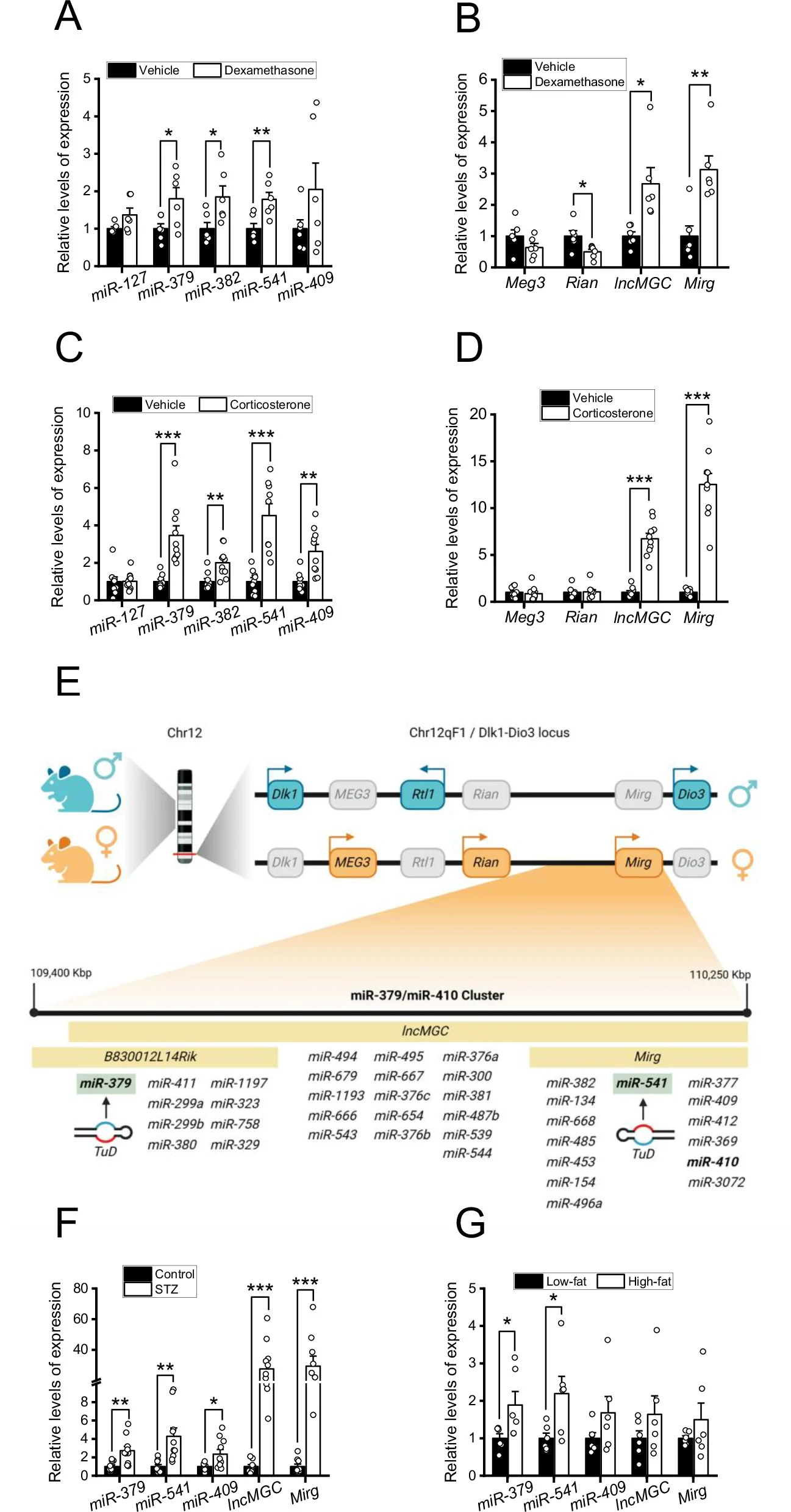
Upregulation of the *miR-379/miR-410* cluster in the liver of experimental models of increased hepatic glucose output and insulin resistance. Levels of expression of miRNAs (**A**) and lncRNAs (**B**) of the *Dlk1-Dio3* locus in the liver of male C57BL/6 J mice on short-term (48 h) dexamethasone exposure vs. vehicle (*n* = 6). Levels of expression of miRNAs (**C**) and lncRNAs (**D**) of the *Dlk1-Dio3* locus in the liver of male C57BL76J mice on long-term (4 weeks) corticosterone exposure (*n* = 10) vs. vehicle (*n* = 9). **E** Schematic representation of the *Dlk1-Dio3* locus on mouse distal chromosome 12. Paternally expressed protein-coding genes are represented in blue, while maternally expressed non-coding RNAs are depicted in orange. Arrows indicate the sense of transcription. The relative positions of lncRNAs and miRNAs of the *miR-379/miR-410* cluster are indicated in the enlarged inset. The position of *miR-379* (at the beginning of the sequence of *lncMGC*) and *miR-541* (in the middle part of the sequence of *Mirg*) is highlighted to indicate the binding sites of our TuD inhibitor. The location of *lncMGC* was represented based on ref.^14^ .Created in BioRender. Alfaro, A. J. (https://BioRender.com/g6yv7xj). Levels of expression of different components of the *miR-379/miR-410* cluster in the liver of male C57BL/6 N mice treated with STZ at 6 weeks of age and then maintained with insulin therapy for 3 months (*n* = 10) vs. vehicle (*n* = 9; **F**), or fed a fructose-palmitate-cholesterol diet for 8 weeks vs. low fat (**G**; *n* = 6). Data are expressed as mean ± SEM; two-tailed Student’s *t* tests were run to compare the levels of expression of the different miRNAs under the various conditions tested; *P < 0.05, **P < 0.01, ***P < 0.001 vs. respective control.

Our previous studies had identified *miR-379* as a mediator of hypertriglyceridemia in experimental models of type 2 diabetes^21^. To identify additional miRNAs from the DLK1-DIO3 locus linked to hepatic insulin resistance, we analyzed hepatic expression in lean subjects and individuals with obesity and normal glycemia but elevated homeostatic model assessment (HOMA-IR) index values (Supplementary Table 1), indicative of a prediabetic state. A large fraction of the miRNAs studied were significantly upregulated in the context of obesity (Supplementary Fig.1A); of all of them, *miR-379* and *miR-541* showed the most robust upregulation (Fig.1A, B). In addition, the levels of expression of both *miR-379* and *miR-541* correlated with different metabolic indicators of increased insulin resistance, such as circulating triglyceride levels and HOMA-IR index values (Fig.1A, B). Notably, upregulation of both miRNAs was confirmed in a larger cohort, with no sex differences in expression (Supplementary Fig. 1B, C). Overall, our data suggest that deregulation of non-coding RNAs from this locus might be of relevance for the development of diabetes and other metabolic alterations in humans. The regulation of these non-coding RNAs was then studied in the liver of mice exposed to short- (48 h) and long-term (4 weeks) glucocorticoid therapy, a stimulus previously demonstrated to induce *miR-379* expression^21^ and a well-established driver of increased insulin resistance and hepatic glucose output. Interestingly, only host transcripts and miRNAs from the *miR-379-miR-410* cluster (particularly *miR-379* and *miR-541*, as previously observed in human samples) were consistently upregulated by glucocorticoid stimulation in mice (Fig. 2A–D). In contrast, upstream non-coding RNAs, including *Meg3*, *Rian* and *miR-127*, were not similarly regulated, indicating independent control of this cluster (Fig. 2E shows a schematic representation of the murine *Dlk1-Dio3* locus). Furthermore, the response of the miRNAs was liver-specific and not detected in other metabolic tissues (such as epididymal and inguinal adipose tissue, brown adipose tissue, and skeletal muscle; Supplementary Fig. 2A–D). Cellular fractionation assays conducted on livers from mice treated with dexamethasone demonstrated that, although both hepatocytes and non-parenchymal cells showed comparable basal expression of different components of the *miR-379/miR-410* cluster, only hepatocytes exhibited a response to glucocorticoid stimulation (Supplementary Fig. 2E–G). Conversely, expression of *miR-127* (representative of the *miR-127/miR-136* cluster) was significantly enriched in the non-parenchymal fraction (Supplementary Fig. 2H). We also investigated whether the hepatic expression of the cluster would mirror the circadian pattern of circulating glucocorticoids. Two canonical clock genes (*Bmal1* and *Per2*), studied as positive controls, and the host transcript *Mirg* demonstrated circadian oscillations (Supplementary Fig. 3A–B). The pattern of expression of *Mirg*, with peak levels right before the dark period, resembled the daily fluctuations of glucocorticoids. In contrast, the mature miRNAs *miR-379* and *miR-541* did not conform to a 24h period, as their expression levels remained constant throughout the day (Supplementary Fig. 3C). This finding is in agreement with previous studies showing inconsistent daily patterns between miRNA precursors and their mature forms^22^. As levels of expression of the maternal non-coding RNAs from the *Dlk1-Dio3* locus were shown to decline after birth^9,16^, we studied expression of the *miR-379/miR-410* cluster in the liver at different times of the life cycle. Expression declined markedly between postnatal day 1 and 12 weeks, but regulatable basal levels persisted for up to 8 months (Supplementary Fig. 3D). Induction of the cluster was not limited to glucocorticoid treatment, as it was also demonstrated in STZ-treated mice and animals fed a fructose-palmitate-cholesterol diet (Fig. 2F, G); suggesting this is a common feature of experimental models of elevated hepatic glucose output and hepatic insulin resistance. A potential role of endoplasmic reticulum (ER) stress in these effects (as previously found in kidney^14^) was discarded by showing no concomitant elevation of ER stress markers (Supplementary Fig. 3E–G).

### Hepatocyte-specific overexpression of *miR-541* is associated with elevated insulin resistance in mice

Given its particularly strong regulation in humans and mice, we next investigated the role of *miR-541* in liver metabolism. To this end, the pre-miRNA sequence was cloned and delivered into the animals within a recombinant AAV (rAAV) vector (serotype 8). Tissue specificity was achieved using the liver specific LP1 promoter^23^. Three weeks after rAAV administration, hepatic *miR-541* expression increased 5-fold (*P* < 0.001), while other non-coding RNAs from the same cluster were unchanged (Supplementary Fig. 4A). Consistent with liver specificity, levels of expression of *miR-541* were unchanged in epidydimal white adipose tissue (Supplementary Fig. 4B). In response to this manipulation, plasma insulin and HOMA-IR values were elevated after a 6 h fast (Supplementary Fig. 4C, D), with a trend (*P* = 0.06) towards increased basal glycemia in morning-fed animals (Supplementary Fig. 4E). In contrast, triglyceride concentrations were unchanged (Supplementary Fig. 4F). Altogether, these findings suggest a role for *miR-541* in the regulation of hepatic glucose homeostasis with no discernable effects on hepatic lipid metabolism.

Together with the previously documented role of *miR-379* in lipid homeostasis, these results suggested that combined inhibition of *miR-379* and *miR-541* might affect both glucose and lipid metabolism. To test this assumption, we designed a molecule simultaneously targeting *miR-541* and *miR-379*, consisting of two antisense oligonucleotides (ASOS), each of them directed to one of the two target miRNAs, coupled to a triantennary GalNAc residue. Both targets were downregulated by 75-85% after 5 weeks of therapy, without affecting other cluster miRNAs (Supplementary Fig. 4G–I); the lowest dose tested was 1 mg/kg and increments in the dose by 3- and 10-fold did not potentiate the downregulation of the targets. Consistent with this, none of the three doses tested affected the levels of expression of the hosts transcript *lncMGC* (Supplementary Fig. 4J). Importantly, triglyceride levels decreased with treatment (Supplementary Fig. 4K), and blood glucose excursions following an exogenous bolus of insulin were also reduced (Supplementary Fig. 4L). This correlated with a trend towards reduced HOMA-IR index values (Supplementary Fig. 4M), supporting effects on glucose homeostasis. These data validate the hypothesis that combinatorial *miR-379* and *miR-541* targeting may exert a multi-parametric improvement in metabolic control.

### Hepatocyte-specific expression of a TuD inhibitor targeting both *miR-541* and *miR-379* disrupts the expression of multiple components of the *miR-379/miR-410* cluster

In order to achieve a more sustained and robust inhibition of the two target miRNAs (*miR-379* and *miR-541*), a TuD inhibitor carrying binding sites for both transcripts was constructed^24^. The activity of the TuD was first tested in vitro, in Hek293A cells transfected with a TuD-coding plasmid under control of the cytomegalovirus promoter. As demonstrated by luciferase assays, the TuD completely prevented the degradation of a target gene (*Rluc* carrying specific binding sites in the 3’-UTR), promoted by the overexpression of either miRNA. Moreover, the inhibition elicited by the TuD with the dual binding site was comparable to the one induced by TuDs specifically designed to target only one of the two miRNAs. As expected, the expression of the TuDs also induced downregulation of the target miRNAs (Supplementary Fig. 5A–F). The TuD was then delivered into mice within a rAAV8 under the control of a highly liver-specific promoter (LP1). Major advantages of this approach include the avoidance of repeated injections and the toxicity of the nucleotide modifications present in ASOS^25^, improved tissue specificity (Supplementary Fig. 6A) and increased availability of the inhibitor due to sustained endogenous production. Moreover, given rAAVs vectors are expressed in the nucleus, the potential interaction of the TuD with host transcripts might affect the processing of other miRNAs from the cluster with related functions, yielding a more robust phenotype.

**Fig. 3:**
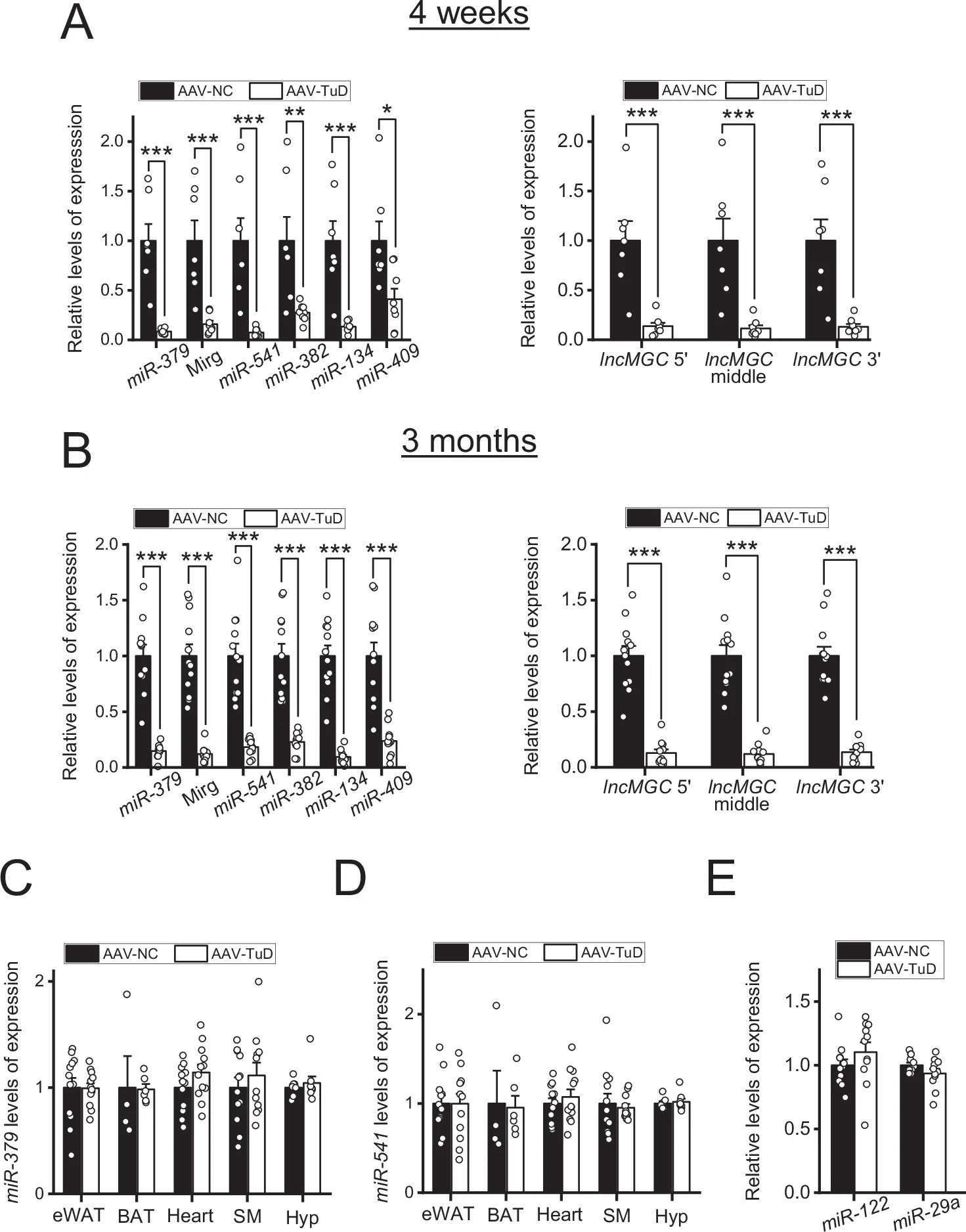
The TuD inhibitor against *miR-379* and *miR-541* promotes a general repression of the *miR-379/miR-410* cluster by targeting the host transcripts *lncMGC* and *Mirg.* Levels of expression of *miR-379, Mirg, miR-541, miR-382, miR-134, miR-410* (left panels) and *lncMGC* (right panels) in the liver of male mice receiving rAAVs and expressing the tough decoy inhibitor (AAV-TuD) or the negative control sequence (AAV-NC) for 4 weeks (**A**; AAV-NC, *n* = 7; AAV-TuD, *n* = 8) or 3 months (**B**; AAV-NC, *n* = 12; AAV-TuD, *n* = 11). Levels of expression of *miR-379* (**C**) and *miR-541* (**D**) in extrahepatic tissues of male mice receiving the rAAV-TuD therapy (eWAT epididymal adipose tissue; BAT brown adipose tissue; SM skeletal muscle; Hyp hypothalamus); eWAT, *n* = 12; BAT (AAV-NC, *n* = 4; AAV-TuD, *n* = 6); Heart, *n* = 12; SM (AAV-NC, *n* = 12; AAV-TuD, *n* = 11); Hyp (AAV-NC, *n* = 7; AAV-TuD, *n* = 8). **E** Levels of expression of two control miRNAs (*miR-122* and *miR-29a*) in male mice receiving the same rAAV therapy; (AAV-NC, *n* = 12; AAV-TuD, *n* = 11). Data are expressed as mean ± SEM, two-tailed Student’s *t* test were used to compare the levels of expression of the different non-coding RNAs under the various conditions tested; *P < 0.05, **P < 0.01, ***P < 0.001 vs. respective control.

Following tail vein injection into wild-type, chow fed mice, expression of the TuD promoted substantial downregulation of the targets, *miR-541* and *miR-379* (by >90%; *P* < 0.001), observed as early as 4 weeks after rAAV administration (Fig.3A) and it was prolonged for at least 3 months (Fig.3B). As expected, the expression of the targets in other tissues was not affected (Fig.3C, D and Supplementary Fig. 6B), highlighting the tissue specificity of the approach. Most interestingly, the levels of expression of two host transcripts of the *miR-379/miR-410* cluster, *lncMGC* and *Mirg*, were also significantly decreased (by 90%, *P* < 0.001; Fig.3A, B). Accordingly, the expression of other miRNAs from the cluster, *miR-382*, *miR-134* and *miR-409*, were also downregulated (Fig.3A, B). In contrast, the expression of hepatic miRNAs external to the cluster (*miR-122* and *miR-29a*) was not affected (Fig.3E), demonstrating specificity for the *miR-379/miR-410* cluster. Similar repression occurred in primary hepatocytes, where the TuD prevented dexamethasone-induced upregulation of *miR-541* and *Mirg* (Supplementary Fig. 6C, D). Consistent with cluster repression, known targets such as RICTOR^26^, *Atf3*^14^ and *Dlk1*^17,27^ were significantly increased (Supplementary Fig. 6E–I). Importantly, the expression of *Dlk1*, was upregulated in the liver but not in epididymal fat and skeletal muscle following miRNA repression (Supplementary Fig. 6I), supporting tissue-specificity of the effects.

Of note, no obvious toxic effects were reported in mice fed regular chow expressing the TuD sequence. Body weight, as well as food and water intake remained comparable between the TuD group and the animals expressing the NC sequence over a period of 3 months (Supplementary Fig. 7A–C) and body composition was also unchanged (Supplementary Fig. 7D). Furthermore, circulating levels of different indicators of liver (ALT, AST, ALP) and kidney (creatinine) damage remained unchanged and within the normal range (Supplementary Fig. 7E, F). Complementarily, histological inspection of the liver showed no obvious signs of inflammation^28^ (Supplementary Fig. 7G). Finally, mice expressing either the TuD or the NC sequence were studied in the PhenoMaster system for 2 weeks. No changes in food intake, locomotor activity or energy expenditure were observed (Supplementary Fig. 8), demonstrating the overall absence of obvious toxicity and/or side effects of *miR-379/miR-410* cluster targeting.

### Hepatocyte-specific repression of the *miR-379/miR-410* cluster induces profound metabolic effects in adult mice fed regular chow

**Fig. 4:**
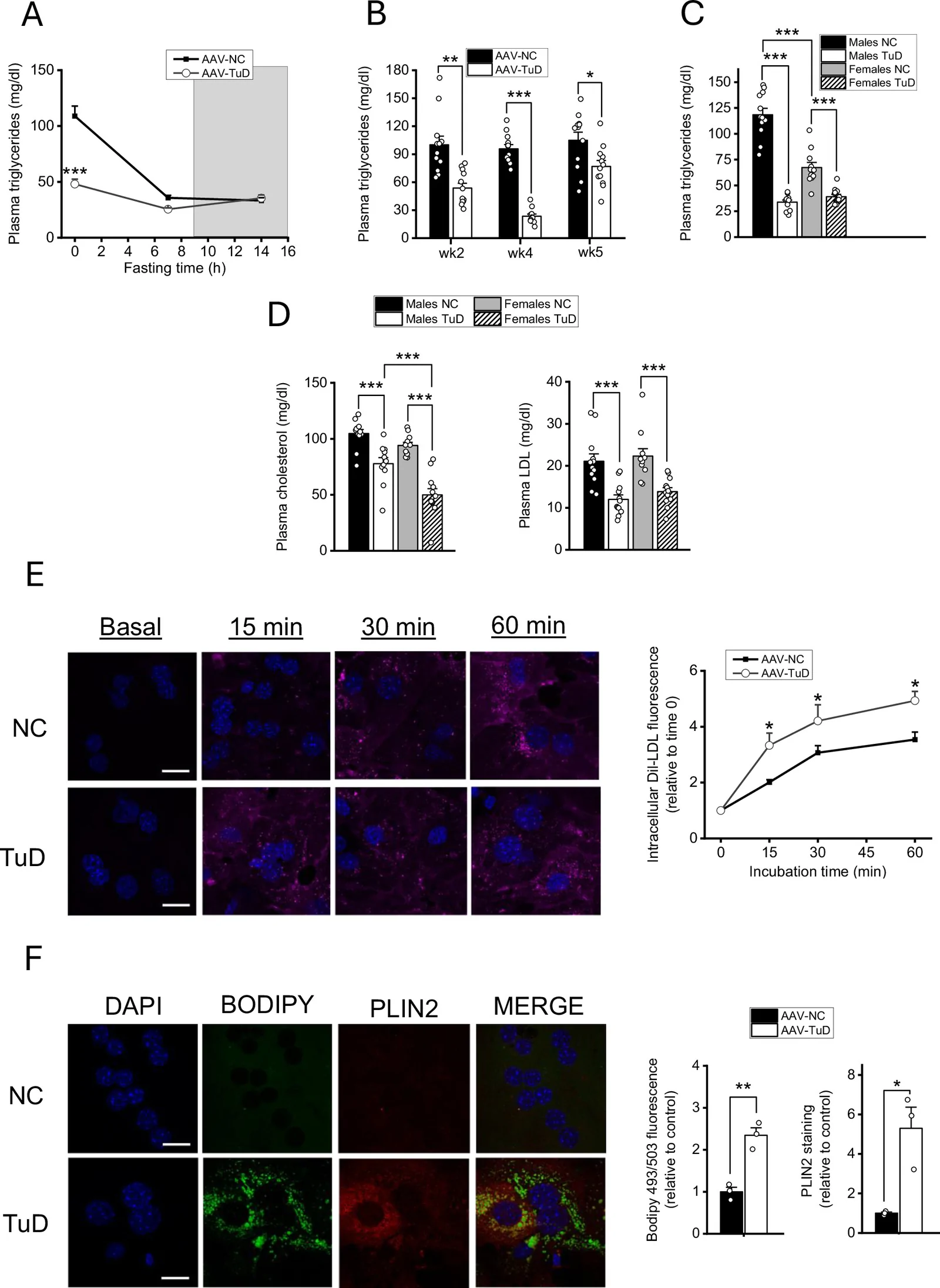
Effects of hepatic-specific targeting of the *miR-379/miR-410* cluster on lipid homeostasis. **A** Circulating triglyceride levels in morning-fed conditions and after 7 and 14 h of fasting in male animals receiving AAV-NC or AAV-TuD therapy (*n* = 7; the shadowed area indicates the dark period). **B** Postprandial triglyceride concentrations (ZT16) at different times after rAAV administration in male mice (*n* = 12). **C** Comparison of the effects of AAV-TuD therapy on postprandial triglycerides in male and female mice (female+AAV-NC, *n* = 11; remaining groups, *n* = 12). **D** Postprandial total (left panel) and LDL (right panel) cholesterol levels in both male and female mice receiving the AAV-TuD therapy vs. NC (female+AAV-NC and male+AAV-TuD, *n* = 11, remaining groups, *n* = 12). **E** Representative confocal microscopy images of male primary hepatocytes expressing the TuD or NC sequence and incubated with Dil-LDL for different time points, 48 h after seeding. On the right, time course profile of LDL internalization (*n* = 3). Scale bars, 10 µm. **F** Representative confocal microscopy images of male primary hepatocytes expressing the TuD or NC sequence and stained with DAPI (blue), BODIPY (green), and anti-PLIN2 antibody (red); and quantification of BODIPY and PLIN2 staining on the right (*n* = 3). Scale bars, 10 µm. Data are expressed as mean ± SEM; results from 4A-E were analysed by two-way ANOVA followed by Holm-Sidak multiple comparison test, while the results from 4 F were analysed by two-tailed Student’s *t* test; *P < 0.05; **P < 0.01; ***P < 0.001 vs. respective control.

Expression of the TuD triggered significantly reduced circulating triglyceride concentrations irrespective of sex (Fig.4A–C). This effect was particularly robust under postprandial conditions, suggesting improved chylomicron clearance. In addition, circulating levels of total and LDL cholesterol were also significantly diminished in both male and female animals (Fig.4D). Remarkably, enhanced LDL uptake was observed in dispersed hepatocytes in culture expressing the inhibitor, indicating that the effect on lipoprotein clearance is liver specific (Fig.4E). Supporting this notion, increased lipid accumulation was observed in primary hepatocytes expressing the decoy and incubated with 10% FBS for 48 h (Fig.4F). However, hepatic triglyceride content remained normal in chow-fed mice expressing the TuDs (Supplementary Fig. 9A, B). Along with the reduced triglyceride and LDL levels and enhanced lipoprotein clearance in primary hepatocytes, the levels of expression of major determinants for lipid uptake (*Lpl*, *Cd36* and *Lipa*) were significantly upregulated (Supplementary Fig. 9C, D). Interestingly, bioinformatic analysis predicted these genes to represent direct targets for several miRNAs from the *miR-379/miR-410* cluster (Supplementary Fig. 9E).

**Fig. 5:**
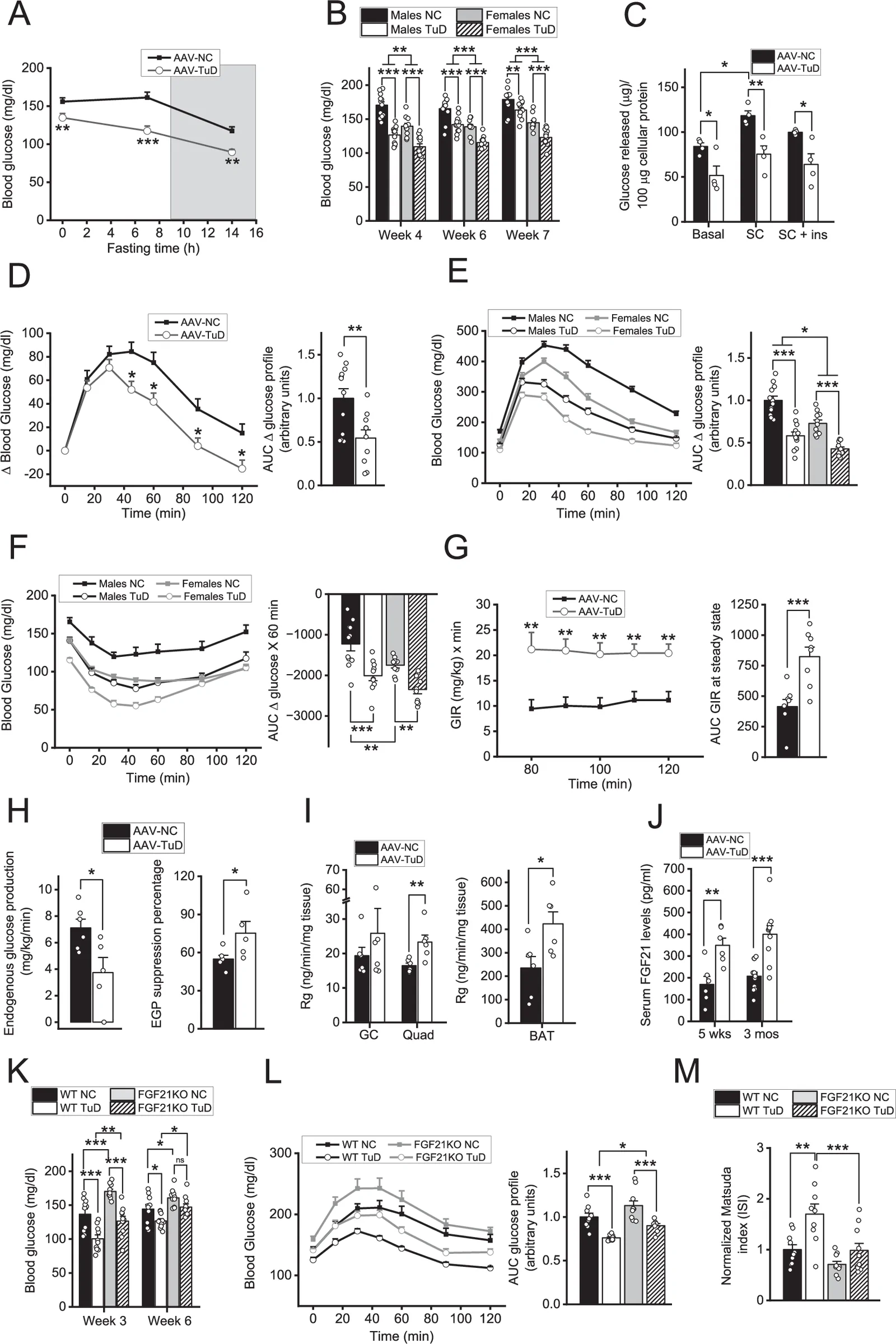
Effects of hepatic-specific targeting of the *miR-379/miR-410* cluster on glucose homeostasis. **A** Blood glucose levels in morning-fed conditions and after 7 and 14 h of fasting in male animals receiving AAV-NC or AAV-TuD therapy (*n* = 7; the shadowed area indicates the dark period). **B** Short-term fasting (6 h) blood glucose concentrations at different times after rAAV administration in both male and female mice (female+AAV-NC at all time points, and male+AAV-NC on weeks 6 and 7, *n* = 11; remaining of the groups, *n* = 12). **C** Glucose production from male primary hepatocytes in culture expressing the TuD or NC sequence and incubated with the stimulation cocktail, with or without insulin, or vehicle for 24 h (*n* = 4). **D** Time course profile of Δ blood glucose concentrations following a pyruvate tolerance test (2 g/kg) 12 weeks after rAAV administration in male mice and corresponding AUC (AAV-NC, *n* = 12; AAV-TuD, *n* = 10). Time course profiles of blood glucose levels in response to an ipGTT (2 g/kg; **E** male groups, *n* = 12; female groups, *n* = 11) and an ipITT (0.7 IU/kg; **F** male+AAV-NC, *n* = 11; remaining groups, *n* = 10), and corresponding AUCs, 4 and 6 weeks after rAAV administration, respectively in both male and female mice. **G** Time course profile of glucose infusion rate (left panel) and corresponding AUC (right panel) during a hyperinsulinemic-euglycemic clamp conducted in male mice, 6 weeks after rAAV administration (*n* = 8). **H** Endogenous glucose production (Ra, right panel) and insulin-mediated percent suppression of endogenous Ra (left panel) in male mice during a hyperinsulinemic-euglycemic clamp (AAV-NC, *n* = 6; AAV-TuD, *n* = 5). **I** Tissue glucose uptake (Rg) in skeletal muscle (left panel) and brown adipose tissue (right panel) of male mice after the hyperinsulinemic-euglycemic clamp (*n* = 6). **J** Circulating levels of FGF21 in male mice after a short period of fasting (6 h), 5 weeks (*n* = 6) and 3 months (AAV-NC, *n* = 12; AAV-TuD, *n* = 10) after rAAV administration. **K** Short-term fasting (6 h) blood glucose concentrations at different times after rAAV administration in male FGF21KO and WT mice (*n* = 10). **L** Time course profile of blood glucose concentrations following a pyruvate tolerance test (2 g/kg) 5 weeks after rAAV administration and corresponding AUC in male FGF21KO (AAV-NC, *n* = 10; AAV-TuD, *n* = 8) and WT mice (*n* = 9). **M** Matsuda insulin sensitivity index calculated from glucose and insulin levels following and OGTT (3 g/kg) in male FGF21KO and WT mice, 3 weeks after rAAV administration (WT + AAV-NC, *n* = 9; remaining groups, *n* = 10). Data are expressed as mean ± SEM; results from 5A-G and 5K-M were analysed by two-way ANOVA followed by Holm-Sidak multiple comparison test, while the results from 5H-J were analysed by two-tailed Student’s *t* test; *P < 0.05; **P < 0.01; ***P < 0.001 vs. respective control.

TuD therapy also improved glucose homeostasis in chow-fed mice of both sexes, significantly reducing basal glycemia (Fig.5A, B) without affecting the circulating levels of two of the most important substrates for gluconeogenesis, lactate and glycerol^29^ (Supplementary Fig. 10A, B). The decrease in blood glucose concentrations after a short period of fasting (6 h) was maintained for up to 7 weeks (Fig.5B), while diminished glucose production in dispersed hepatocytes expressing the decoy was also found (Fig.5C). Accordingly, mRNA levels of key gluconeogenic enzymes (*Pck1* and *G6pc*) were significantly decreased in vitro and their response to dexamethasone stimulation completely prevented (Supplementary Fig. 10C, D). Pyruvate tolerance tests showed reduced glucose excursions up to 3 months after rAAV administration (Fig.5D and Supplementary Fig. 10E), despite normalization of basal glucose. Hepatic glycogen content was dramatically reduced after 6 h of fasting (Supplementary Fig. 10F), indicating preserved glycogen breakdown. Along with the reduction in glycemia, liver-specific expression of the TuD was also associated with marked improvements in glucose clearance irrespective of sex (Fig.5E). This effect was not driven by an exacerbated insulin secretory response, as demonstrated by normal insulin concentrations in response to an oral glucose load (Supplementary Fig. 10G). In fact, basal insulin levels were lower in TuD-treated animals (Supplementary Fig. 10H), and insulin tolerance improved, again in both sexes (Fig.5F), indicating better insulin sensitivity. Plasma concentrations of representative counter-regulatory hormones (corticosterone and glucagon) remained comparable between the two groups (Supplementary Fig. 10I-J), precluding defective secretion of these hormones. The improved insulin sensitivity in the group expressing the TuD was further confirmed by euglycemic-hyperinsulinemic clamp studies, which showed a 2-fold increase in glucose infusion rate (*P* < 0.001) in this group, as compared to negative control (Fig.5G and Supplementary Fig. 10K, L). Moreover, endogenous glucose production was also significantly lower in the animals expressing the decoy (Fig.5H). Finally, uptake of 2-deoxy-glucose was also significantly enhanced in skeletal muscle and brown adipose tissue by the TuD therapy (Fig.5I), indicating superior insulin action in extrahepatic tissues; an observation that could be partially explained by the upregulation (Supplementary Fig. 10M) and increased circulating levels (Fig.5J) of FGF21, a hepatokine known to modulate glucose uptake by peripheral tissues^30,31^. To test this hypothesis, rAAVs expressing either the TuD inhibitor or a negative control sequence were delivered to Fgf21 KO mice^32^. As anticipated, the 2.5-fold increment in FGF21 circulating levels elicited by the TuD therapy in WT animals was abolished in Fgf21 KO mice (Supplementary Fig. 10N). Lipid-lowering effects remained unchanged in Fgf21 KO mice (Supplementary Fig.10O), but reductions in fasting glucose and PTT excursions were attenuated (Fig.5K, L). Glucose clearance remained improved (Supplementary Fig. 10P), but insulin sensitivity was partially reduced, as indicated by the insulin sensitivity index (ISI; Fig.5M) and the HOMA-IR (Supplementary Fig. 10Q). Collectively, our findings demonstrate that the *miR-379/miR-410* cluster plays a major role in the regulation of lipoprotein uptake by the liver, as well as in the regulation of hepatic glucose output and hepatic insulin sensitivity in young adult mice, effects that have an impact in whole-body energy homeostasis. Although not instrumental, the elevation in FGF21 levels partially contributes to the effects on glucose homeostasis.

### Hepatocyte-specific repression of the *miR-379/miR-410* cluster enhances insulin and mTOR signaling and activates mitochondrial function in the liver

**Fig. 6:**
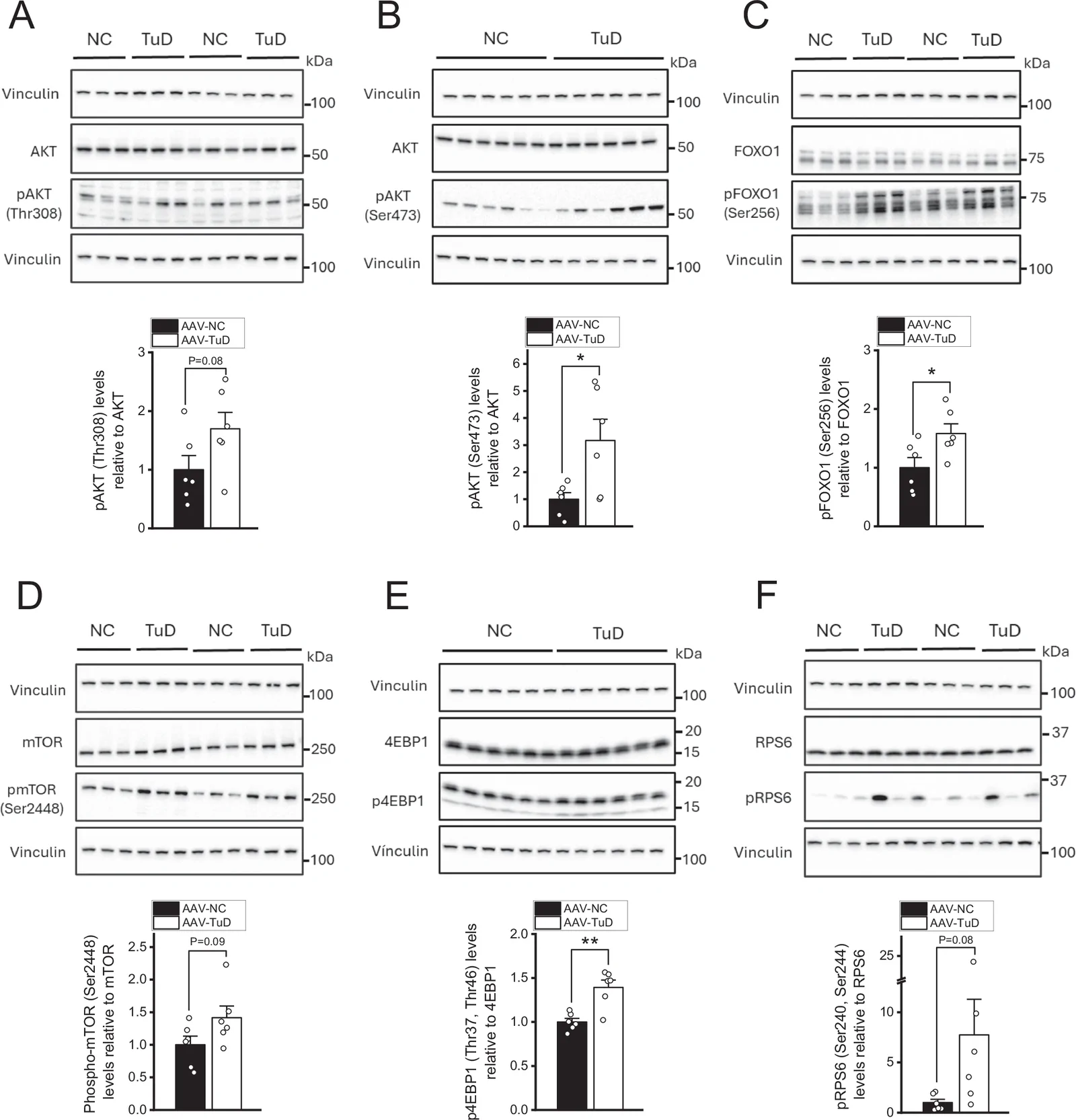
Activation of PI3K/mTOR signaling pathway in the liver of animals expressing the TuD inhibitor against *miR-379/miR-410* cluster. Immunoblots to study PI3K signaling activation; including total and phosphorylated-AKT at Thr308 residue (**A**); total and phosphorylated-AKT at Ser473 residue (**B**); and total and phosphorylated-FOXO1 at Ser256 residue (**C**) in the liver of male animals receiving AAV-NC or AAV-TuD therapy (*n* = 6) and corresponding densitometric quantification. Immunoblots to study mTOR signaling activation; including total and phosphorylated-mTOR at Ser2448 residue (**D**); total and phosphorylated-4EBP1 at residues Thr37 and Thr46 (**E**); and total and phosphorylated-RPS6 at residues Ser240 and Ser244 (**F**) in the liver of male animals receiving AAV-NC or AAV-Tud therapy (*n* = 6) and corresponding densitometric quantification. Immunoblots for every protein are shown with respective loading control (vinculin). Data are expressed as mean ± SEM; two-tailed Student’s *t* tests were run to analyze the densitometric results; *P < 0.05; **P < 0.01 vs. respective control.

To explore underlying mechanisms, predicted targets of *miR-379*, *miR-541* and other miRNAs repressed by our TuD (*miR-382*, *miR-134* and *miR-409*) were identified by bioinformatic analysis. Pathway enrichment analysis revealed PI3K/mTOR signaling pathway as a common target of these miRNAs (Supplementary Fig. 11). RNA-sequencing analysis from liver samples expressing either the TuD or the NC sequence confirmed these predictions, as entries related to the PI3K/mTOR signaling pathway such as “focal adhesion”, “regulation of actin cytoskeleton” and “PI3K-AKT signaling” were among the top upregulated pathways by TuD therapy (Supplementary Fig. 12). Because miRNAs often regulate target expression by affecting translation and not mRNA stability, proteomic analysis was also conducted to complement these findings (Supplementary Fig. 13A–C). As determined by gene set enrichment analysis, the top upregulated pathways in the animals expressing the decoy include pathways involved in cholesterol and protein synthesis. Conversely, pathways related to biological oxidations, amino acid metabolism, as well as bile acid and fatty acyl-CoA synthesis, were among the top downregulated ones (Supplementary Fig. 13D, E). Interestingly, GKAP1 was strongly upregulated (fold-change 11.6; Supplementary Fig. 13C and 13F) and predicted as a direct target of multiple cluster miRNAs (Supplementary Fig. 13G). GKAP1 is known to activate insulin signaling, and repression of this protein has been linked to the occurrence of insulin resistance^33^. Immunoblots showed elevated AKT (Thr308 and Ser473) and FOXO1 phosphorylation (Fig.6A–C), indicating enhanced insulin signaling, in line with the upregulation of RICTOR and the superior hepatic insulin sensitivity demonstrated by the euglycemic-hyperinsulinemic clamp. Complementarily, mTOR signaling was also found to be activated, as demonstrated by augmented phosphorylation of mTOR, 4EBP1 and Ribosomal protein S6 (Fig.6D–F). Conversely, a similar outcome was not observed in skeletal muscle samples from the same animals, demonstrating that the effect is liver specific and not secondary to whole-body improvement in insulin sensitivity (Supplementary Fig. 14).

**Fig. 7:**
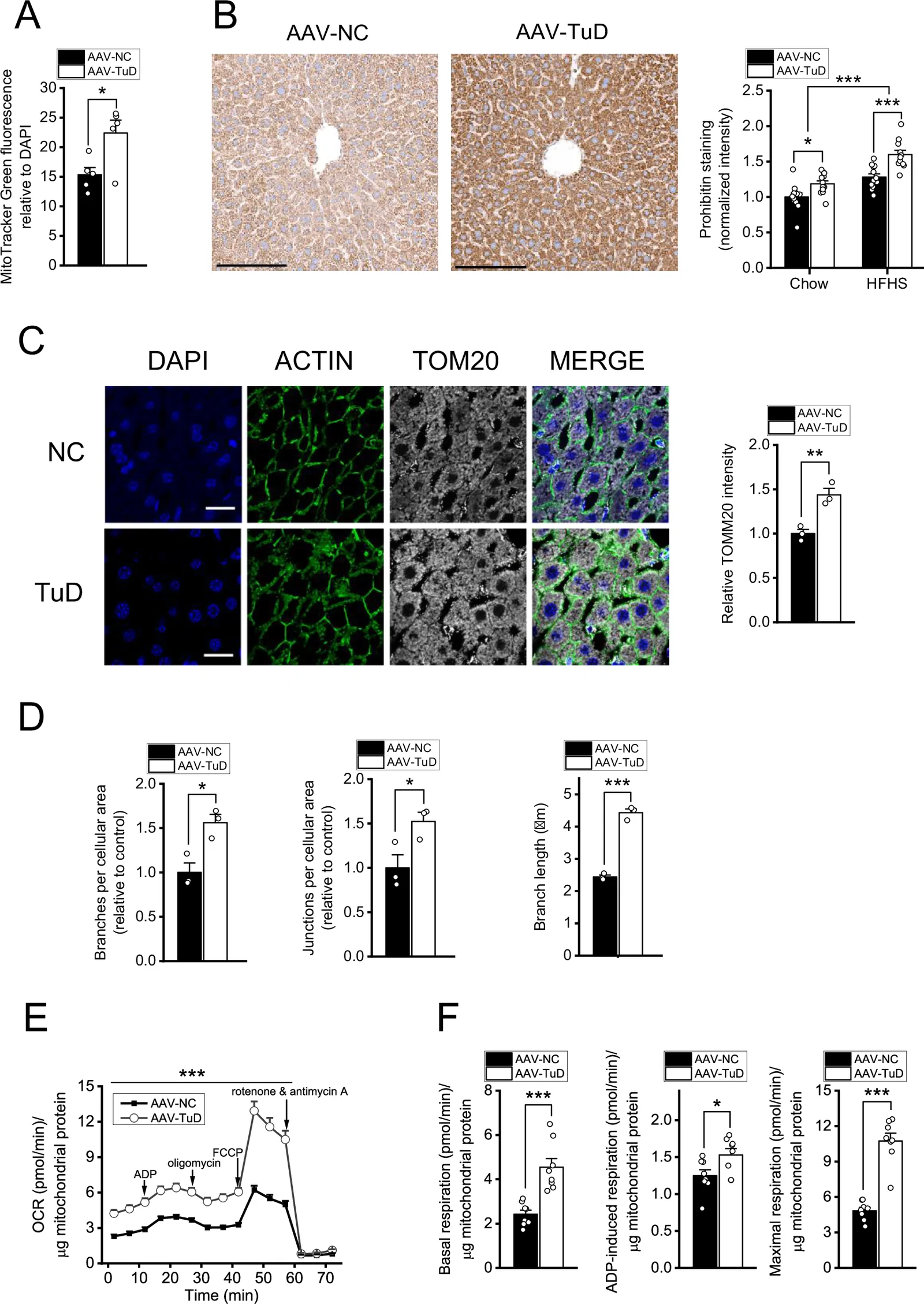
Effects of *miR-379/miR-410* cluster silencing on mitochondrial biology. **A** MitoTracker Green staining of male primary hepatocytes expressing the TuD or NC sequence (*n* = 5). **B** Representative microscopy images of prohibitin staining from liver sections of male mice receiving AAV-TuD or AAV-NC therapy for 5 weeks (left panel) and quantification of the liver sections from all the animals (right panel; HFHS + AAV-TuD, *n* = 11; remaining groups, *n* = 12). Scale bars, 200 µm. **C** Representative confocal microscopy images of liver sections stained with DAPI (blue), phalloidin (green) and TOM20 (grey) of male mice receiving AAV-TuD or AAV-NC therapy for 6 weeks (left panel) and corresponding quantification of the intensity from all the sections (right panel; *n* = 3). Scale bars, 25 µm. **D** Quantification of the number of branches, number or junctions per area, and mean length of the branches using Fiji (*n* = 3 biological replicates/group; 2 slides were studied per animal with 6 technical replicates per slide). **E** Oxygen consumption rate, measured with a Seahorse Analyzer, of mitochondrial extracts, isolated from livers of male animals expressing AAV-TuD or AAV-NC 7 weeks after rAAV administration, in response to basal conditions (5 mM pyruvate and 5 mM malate), as well as to sequential incubation with ADP (3.5 mM), oligomycin (3.5 µM), FCCP (4 µM), and rotenone (2 µM) and actinomycin A (4 µM; *n* = 8 biological replicates/group). **F** Basal respiration, ADP-induced respiration and maximal respiration from the Seahorse Assay shown in (**E**). Data are expressed as mean ± SEM; results from 7 A, 7C-D, and 7 F were analysed by two-tailed Student’s *t* test, while results from 7B and 7E by two-way ANOVA followed by Holm-Sidak multiple comparison test; *P < 0.05; **P < 0.01; ***P < 0.001 vs. respective control.

Untargeted metabolomic revealed that various amino acids and intermediates of amino acid catabolism were significantly accumulated in the liver of animals expressing the decoy, supporting decreased amino acid catabolism in the tissue (Supplementary Fig. 15A). As many of these amino acids contribute to gluconeogenesis^34^, their accumulation suggests reduce substrate flux into this pathway. In agreement with this, reduced glucose and lactate content was found in the same animals (Supplementary Fig. 15B), also concurring with the improved pyruvate tolerance (Fig.5D). As high intracellular amino acid content is among the most powerful inducers of mTOR signaling^35^, this may also contribute to the mTOR signaling activation observed in the liver. Given the links between mTOR activation and mitochondrial biogenesis^36^, as well as inhibition of the cluster and enhanced mitochondrial activity^26,37^, mitochondrial mass and function were assessed. Mitochondrial mass was significantly enhanced in hepatocytes expressing the TuD, as demonstrated by MitoTracker Green fluorescence in dispersed hepatocytes (Fig.7A) as well as prohibitin (Fig.7B) and TOM20 staining of liver samples (Fig.7C). In addition, mitochondrial connectivity was also found to be increased (Fig.7D), as were the levels of expression of many mitochondrial proteins (Supplementary Fig. 16A) and critical genes for the regulation of mitochondrial activity not detected with the proteomic analysis, *Cyptb1b* and *Ucp2* (Supplementary Fig. 16B). In line with this, oxygen consumption rate increased under basal, ADP-induced and maximal respiration (Fig.7E, F) in mitochondrial extracts with comparable mitochondrial protein content (Supplementary Fig. 16C–E). Furthermore, pyruvate carboxylase as well as pyruvate dehydrogenase kinase 1 and 2 abundance decreased, favoring mitochondrial pyruvate oxidation (Supplementary Fig. 16F). These findings demonstrate elevated mitochondrial function, likely contributing to the overall favorable metabolic phenotype.

### Hepatocyte-specific silencing of the *miR-379/miR-410* cluster improves glycemic control and dyslipidemia in diabetes and diet-induced obesity

**Fig. 8:**
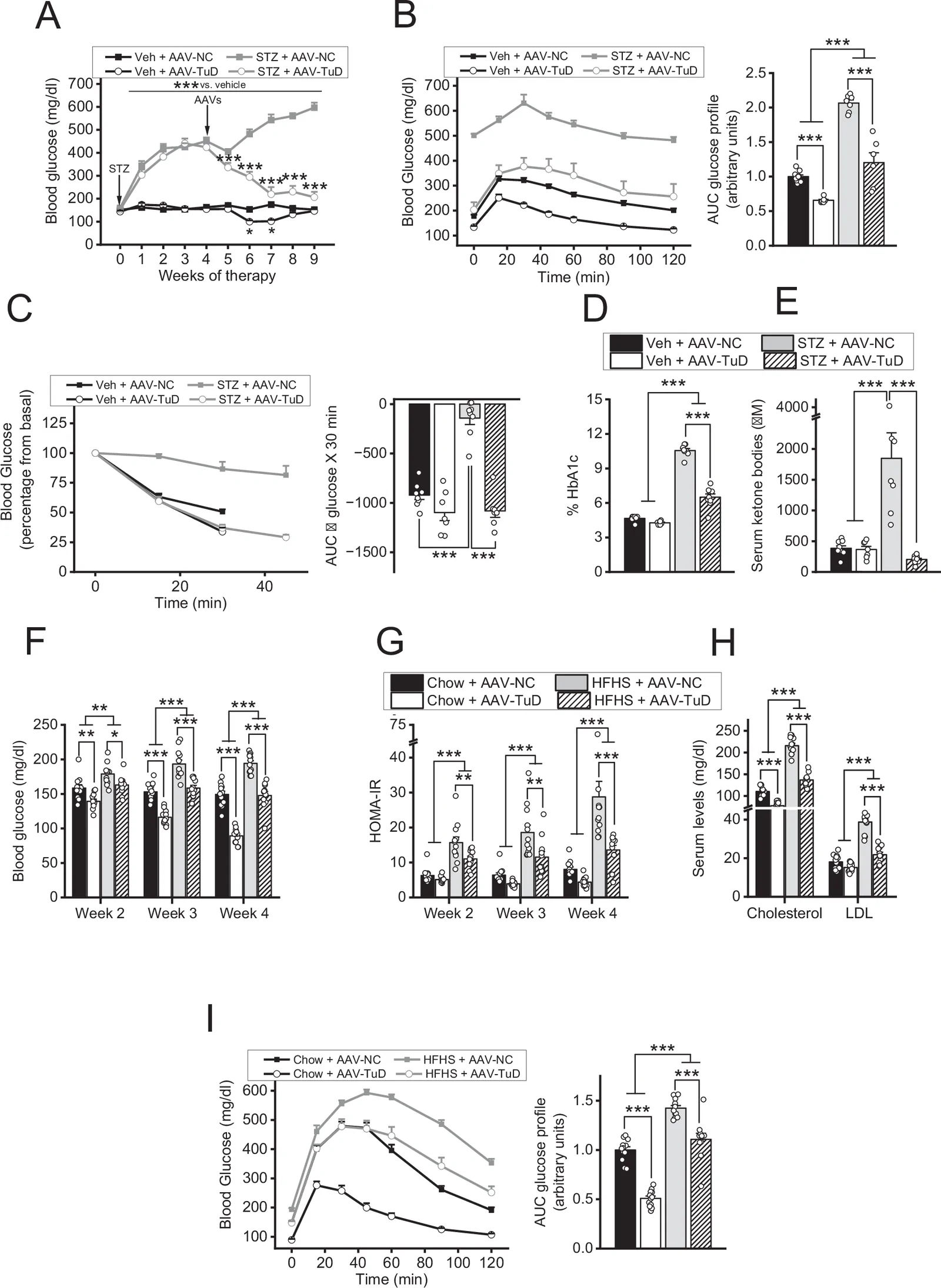
Hepatic-specific repression of the *miR-379/miR-410* cluster improves glycemic control and dyslipidemia in male models of type I diabetes and diet-induced obesity. **A** Weekly determination of morning-fed glucose levels in male STZ-induced diabetic mice receiving either AAV-NC or AAV-TuD therapy (Veh+AAV-NC, *n* = 10; Veh+AAV-TuD, *n* = 7; STZ + AAV-NC, *n* = 8; STZ + AAV-TuD, *n* = 9). The arrows indicate the time of induction of diabetes (STZ) and the time of AAV administration. Time course profile of blood glucose concentrations in some of the animals studied in A, in response to an ipGTT (1 g/kg; **B** Veh+AAV-NC, *n* = 10; Veh+AAV-TuD, *n* = 7; STZ + AAV-NC, *n* = 7; STZ + AAV-TuD, *n* = 6) and an ipITT (0.7 IU/kg; **C** Veh+AAV-NC, *n* = 8; Veh+AAV-TuD, *n* = 7; STZ + AAV-NC, *n* = 8; STZ + AAV-TuD, *n* = 7); and corresponding AUCs. The ipITT was stopped at time 30 in the non-diabetic (vehicle-treated) animals given the large number of animals with blood glucose levels dropping below 50 mg/dl. Percentage of glycated hemoglobin (HbA1c; **D**) and serum ketone body levels (**E**) in animals from the same experiment at termination (Veh+AAV-NC, *n* = 10; Veh+AAV-TuD, *n* = 7; STZ + AAV-NC, *n* = 8; STZ + AAV-TuD, *n* = 9; ketone body levels measurements are missing in one animal from the Veh+AAV-NC group and another from the STZ + AAV-NC group due to limited sample volume). Weekly determination of fasting (6 h) glucose levels (**F**) and corresponding HOMA-IR values (**G**) in male diet-induced obese animals receiving either AAV-NC or AAV-TuD therapy (HFHS + AAV-TuD=11; remaining groups, *n* = 12). Total cholesterol and LDL levels (**H**), and time course profile of blood glucose concentrations following an ipGTT (2 g/kg) with corresponding AU (**I**) in the same animals as in (**F**, **G**). Data are expressed as mean ± SEM; two-way ANOVA followed by Holm-Sidak multiple comparison test was used to compare the different groups; *P < 0.05; **P < 0.01; ***P < 0.001.

Finally, to test the therapeutic potential of our TuD inhibitor, we employed streptozotocin (STZ)-treated mice as a model for type 1 diabetes. As expected, the animals developed severe hyperglycemia shortly after the STZ injections. Remarkably, as early as 1 week after the administration of rAAVs, the animals receiving TuD therapy displayed significantly reduced morning glycemia (by 13%; *P* < 0.001). Blood glucose levels continued to decrease over time, to the point that they were almost normalized 5 weeks after rAAV administration ( > 60% reduction; *P* < 0.001; Fig.8A). According to this, the levels of expression of *Pck1* in the liver were markedly diminished indicating substantial reduction in hepatic glucose output (Supplementary Fig. 17A). Glucose handling and insulin tolerance were also vastly improved in the STZ-treated animals expressing the TuD (Fig.8B, C). Importantly, these effects were independent of pancreatic changes, as α- and β-cell mass and their ratio were unchanged, and insulin levels remained low in both STZ groups (Supplementary Fig. 17B, C). The improved glycemic control was further supported by marked reduction in the fraction of glycated hemoglobin (Fig.8D), normalization of the ketonemia (Fig.8E), as well as the reduced water intake (Supplementary Fig. 17D). Triglyceride and NEFA levels were also reduced (Supplementary Fig. 17E, F), indicating broad metabolic improvement.

Further, mice fed a HFHS diet were used as a model of diet-induced obesity, displaying hyperglycemia, hyperinsulinemia, hypercholesterolemia and marked defects in glucose clearance (Fig.8F–I). Hepatocyte-specific expression of the TuD sequence strongly improved all these parameters (Fig.8F–I), while it also reduced circulating triglyceride levels and attenuated fat deposition in the animals (Supplementary Fig. 18A, B). Consistent with lower glycemia, levels of expression of key gluconeogenic enzymes were diminished in the liver (Supplementary Fig. 18C); while, in agreement with the significantly smaller fat depots, HFHS-fed mice receiving the TuD therapy also displayed reduced body weight at termination (Supplementary Fig. 18D). Of note, these effects were not dependent on differences in food intake since the daily consumption of calories was similar between the animals expressing the TuD and the negative control group (Supplementary Fig. 18E). Finally, despite showing a higher degree of liver steatosis (Supplementary Fig. 9A, B) and concomitant mild increments in ALT and AST circulating levels (Supplementary Fig. 18F), the obese mice on TuD therapy did not manifest signs of liver inflammation (Supplementary Fig. 18G).

Altogether, our data showed substantial improvements in glycemic control and dyslipidemia in two widely used experimental models of metabolic disease in response to hepatocyte-specific, combinatorial silencing of miRNAs from the *miR-379/miR-410* cluster, thereby providing first proof-of-concept for the usability and efficacy of combinatorial miRNA targeting in obesity and diabetes therapy.

## Discussion

Non-coding RNAs from the *Dlk1-Dio3* locus are indispensable for embryonic development and fetuses with paternal uniparental disomy for chromosome 12 (which do not express the maternal non-coding RNAs) do not survive to term^38^. They are also critical for postnatal development, as reduced gene dosage causes early neonatal lethality^16,39^. The fact that these animals fail to maintain body temperature and normoglycemia supports a role for these miRNAs in the maturation of metabolic organs such as the brown adipose tissue and the liver. Although rare, imprinting disorders affecting the human DLK1-DIO3 locus on chromosome 14q32 have also been described. Individuals affected with these disorders often manifest physical defects and require obligatory supportive therapies early in life. Gene imbalances leading to reduced dosage of the maternal non-coding RNAs cause Kagami-Ogata syndrome, characterized by multiple congenital abnormalities such as abnormal formation of the thoracic cavity. In contrast, excess dosage of the non-coding RNAs leads to Temple syndrome, characterized by low birth weight and endocrine anomalies like precocious puberty, obesity, diabetes and hypercholesterolemia^40^. Supporting a role for these non-coding RNAs in the development of endocrinopathies, genome-wide analyses have revealed enhanced expression of these transcripts in atherosclerotic plaques^41^. Herein, we demonstrate that miRNAs from the *miR-379/miR-410* cluster are robustly upregulated in the liver of different experimental models associated with hepatic insulin resistance and increased hepatic glucose output, including animals on glucocorticoid therapy, treated with streptozotocin, or fed a MASH-inducing diet. Indicating that, despite postnatal decline, these miRNAs remain inducible in adulthood. In agreement with this, upregulation of components of the *miR-379/miR-410* cluster has also been reported in models of persistent mTOR signaling activation^42^, as well as models of type 2 diabetes^21^. Most interestingly, we also provide evidence that miRNAs from the *Dlk1-Dio3* locus, particularly *miR-379* and *miR-541*, are also upregulated in subjects with obesity and insulin resistance. This observation confirms previous findings in the lab regarding *miR-379* expression^21^, and it is consistent with reports from other groups showing enrichment of *miR-379* and other miRNAs from the cluster in the serum of patients with NAFLD^43,44^. Altogether this demonstrates conservation across species and supports translational relevance. Interestingly, comparable levels of expression of *miR-379* and *miR-541* were detected between males and females in both lean subjects and individuals with obesity, indicating lack of sex differences in the hepatic expression of the cluster.

Previous works have reported coregulation of the different components of the *miR-379/miR-410* cluster in response to various stimuli; MF2A in neurons^45^ and skeletal muscle^37^, TGFβ1 and CHOP in kidney cells^14^, and glucocorticoids in liver^21^. These studies demonstrate that the cluster is independently regulated from the lncRNAs located in the proximal (centromeric) end of the locus. Supporting this, here we report consistent upregulation of the cluster in response to glucocorticoid stimulation without parallel changes in *Meg3*, *Rian* or the *miR-127/miR-136* cluster. The coregulation of the miRNAs from the cluster suggests that they may derive from a common long primary transcript, identified as *lncMGC* in the kidney^14^. Our data show that *miR-379* and *miR-541* are particularly responsive to the upregulation of the cluster, suggesting differences in stability or processing. We have previously demonstrated a role for *miR-379* in the control of lipoprotein clearance by the liver^21^; herein, we show that hepatic overexpression of miR-541 increases insulin and glucose concentrations, as well as HOMA-IR values, indicating a potential role of this miRNA in insulin sensitivity. Supporting this, silencing both miRNAs with an inhibitor carrying two LNA-based ASOS, conjugated with a GalNAc moiety to improve liver specificity^46^, promoted reductions in circulating levels of triglycerides and enhanced the action of exogenous insulin.

As ASOS fail to localize in the nuclei of hepatocytes when administered in vivo^46–48^, they are known to not affect the nuclear precursors of miRNAs^4,48^, accordingly our ASOS failed to target the host transcripts *lncMGC* and *Mirg*. In contrast, the use of a TuD inhibitor carrying binding sites for both *miR-379* and *miR-541* promoted severe downregulation of not only the intended miRNA targets, but also of the host transcripts *lncMGC* and *Mirg*. Hence, several other miRNAs from the cluster hosted by these lncRNAs were also repressed. Persistent nuclear accumulation of the TuD^49^ likely contributed to this effect. The improved ability of TuDs to promote degradation of their target sequences as compared to ASOS^24,49,50^ could have also played a role. As opposed to the modest biological effects often induced by the targeting of single miRNAs or a limited number of miRNAs, we show here that combinatorial targeting of a large cluster of miRNAs with related functions results in remarkable metabolic effects. Moreover, as it has recently reported that the host transcript *lncMGC* may exert its own independent effects by interacting with nucleosome remodeling factors^51^, it cannot be discounted that the direct repression of *lncMGC* and *Mirg* may contribute to the metabolic phenotype here reported. Similarly to our own, previous studies have also successfully targeted *lncMGC* with ASOS directed against its transcription start site^14^. However, despite remarkable repression of the lncRNA in the kidney, only modest reduction of the miRNAs from the cluster was achieved; suggesting that host transcripts other than *lncMGC* may be important for the regulation of the *miR-379/miR-410* cluster. In contrast, our TuD induced comparable downregulation of the host transcripts *lncMGC* and *Mirg*, as well as several miRNAs from the cluster in the liver. The reduction in the expression levels of these transcripts was markedly robust ( ≈ 90%), indicating general repression of the cluster. The TuD also allowed us to avoid the cytotoxic effects^25,46^ promoted by the nucleotide modifications present in the ASOS as well as the induction of long-term inhibition of miRNA function^50^ without the need of repeated injections. Finally, the expression of our TuD is driven by a promoter highly liver specific^23,52^, restricting the manipulation of the miRNAs to this particular organ. This clearly separates our study from previous works involving whole-body deletion of the maternally-expressed miRNAs, as repression of these miRNAs has been associated with skeletal muscle hypertrophy^17,53^, enhanced insulin secretory capacity of β-cells^54^, and inhibition of adipogenesis, resulting in marked reduction of adipose tissue mass^19,20^, all of them serious confounder effects for the characterization of a metabolic phenotype.

In agreement with previous studies^26^, our findings indicate that miRNAs from the *miR-379/miR-410* cluster are important regulators of PI3K-mTOR signaling, as multiple components of the pathway are predicted to represent direct targets of these miRNAs. Accordingly, we demonstrate that PI3K-mTOR signaling is indeed activated in hepatocytes in which the cluster is repressed by our TuD inhibitor. This activation is not secondary to the whole-body improvement in insulin sensitivity but rather liver specific, as it is not observed in skeletal muscle. Supporting this, RICTOR, the key component of the mTORC2 complex, is upregulated in response to the repression of the cluster in the liver, but not in the muscle. Since mTORC2 is a major regulator of insulin signaling^55^, upregulation of RICTOR is one of the main mechanisms by which the components of the cluster activate the pathway, while upregulation of GKAP1 may likely contribute^33^. Following the activation of insulin signaling, FOXO1, a key regulator of the gluconeogenic program^56^, is inhibited. In parallel, upregulation of ATF3, another direct target of the miRNAs from the cluster which competes with CREB for the same responsive elements, also inhibits the gluconeogenic program^57,58^. As a result, hepatic expression of critical gluconeogenic enzymes like *Pck1* and *G6pc* is decreased in the animals carrying the TuD and blood glucose levels are markedly reduced; supporting previous studies showing that constitutive deletion of the *miR-379/miR-410* cluster leads to severe hypoglycemia and significant lethality during the weaning period^16^. Moreover, metabolomic analysis revealed accumulation of several glucogenic amino acids in the liver of animals expressing the TuD inhibitor, and proteomics showed that some of the enzymatic transformations that the amino acids undergo to be incorporated in the gluconeogenic pathway^34^ are inhibited. Since amino acids are particularly relevant substrates for gluconeogenesis during short-term fasting^29,59^, defects in the anaplerotic reactions involving amino acids might have also contributed to the reduced hepatic glucose output. Related to this, branched-chain amino acids (BCAA; valine, leucine and isoleucine) are among the top enriched amino acids in the liver, and their catabolism among the top downregulated pathways. Considering the well-stablished role of BCAA as potent inducers of mTOR signaling^60^, this mechanism could have also contributed to the mTOR pathway activation reported here. As mTOR activation stimulates mitochondrial biogenesis and activity, it is likely involved in the enhanced mitochondrial function, mass and connectivity induced by the repression of the cluster in the hepatocytes. The increased mitochondrial oxidation in the hepatocytes with silencing of the *miR-379/miR-410* cluster supports previous findings linking this cluster of miRNAs with restriction of mitochondrial activity in both muscle^37^ and hematopoietic stem cells^26^. Related to this, the proteomic analysis revealed substantially reduced levels of pyruvate carboxylase as well as pyruvate dehydrogenase kinase 1 and 2, ultimately favoring the conversion of pyruvate into acetyl-CoA to be oxidized in the mitochondria over its conversion into oxaloacetate to be utilized in gluconeogenesis^61^, representing yet another mechanism by which hepatic glucose output is reduced in the animals carrying the TuD. Complementary to the reduced hepatic glucose output, hepatic-specific silencing of the *miR-379/miR-410* cluster also improved whole body insulin sensitivity and enhanced glucose uptake by peripheral tissues, as indicated by our hyperinsulinemic-euglycemic clamp results. In this regard, our data indicate that hepatokines such as FGF21KO, able to stimulate glucose disposal, particularly in brown adipose tissue^30,31^ and a well-known insulin sensitizer^62^, contribute to the improvement in insulin sensitivity. Altogether, these effects contribute to the improved glycemic control observed in both animals with deficient insulin secretion (STZ-treated mice) and animals with elevated insulin resistance (diet-induced obese mice). Importantly, the metabolic benefits observed in STZ-treated animals are not driven by the preservation of pancreatic islets and their ability to secrete insulin, as shown in studies involving whole body KO of *lncMGC*^54^.

In line with previous studies demonstrating a role for *miR-379* in the control of lipoprotein clearance by the liver, herein we show that a more extensive repression of the *miR-379/miR-410* cluster not only decreases circulating triglyceride concentrations, but it also diminishes cholesterol and LDL levels. This effect is liver specific, as demonstrated by the enhanced LDL uptake of primary hepatocytes in culture expressing our TuD. Of note, major determinants for lipid uptake including *Lpl*, *Cd36* and *Lipa* are predicted to be direct targets of several miRNAs from the cluster, and they are also amongst the top upregulated genes in livers where the cluster is repressed, suggesting their implication in these effects. Supporting this, LPL is widely known to enhance lipoprotein uptake by the liver^63^, and hepatic LPL de-repression by miRNA targeting has been shown to stimulate hepatic lipid uptake in previous studies^64^. Despite the enhanced lipoprotein uptake, no increased hepatic lipid deposition was observed in animals fed regular chow, likely because of the concomitantly elevated mitochondrial oxidation. However, hepatic triglyceride content was slightly elevated in our model of diet-induced obesity, suggesting that the increased mitochondrial function is not sufficient to overcome the augmented hepatic lipid flux in this model. In contrast, previous studies have found amelioration of hepatic steatosis in high-fat diet fed mice with constitutive deletion of a fragment of the cluster^20^. This discrepancy might be explained by the dramatic reduction in adipose depot mass observed in the constitutive model (likely decreasing the fatty acid influx from the adipose tissue); and by presumably enhanced fatty acid consumption in the skeletal muscle, as the constitutive model presents marked muscular hypertrophy^17^ and inhibition of this cluster in the muscle has demonstrated to locally stimulate mitochondrial function^37^. Considering the increased lipid accumulation and slightly elevated levels of aminotransferases in our obese mice in response to repression of the cluster, the safety of this manipulation in other models of obesity, insulin resistance or MASLD deserves further evaluation as it might exacerbate hepatic steatosis in those models or even accelerate the progression to more advanced stages of liver disease. However, in this regard it must be emphasized that no major signs of inflammation were noted in the liver of our obese model by histological analysis.

Finally, given the relevance of sex as biological factor for metabolism, we studied the effects of hepatocyte-specific repression of the *miR-379/miR-410* cluster in both males and females fed regular chow. As expected, female mice showed reduced circulating levels of triglycerides and fasting glycaemia, as well as improved glucose clearance and reduced glucose excursions in response to pyruvate and insulin tolerance test, as compared to males^65,66^. Despite the profound sex differences, repression of the cluster promoted comparable effects on these parameters irrespective of sex, denoting no sexual variation in the response to this manipulation. However, we failed to evaluate the effect of sex in our models of diet-induced obesity and STZ-induced diabetes as only males were used. We acknowledge this as an important limitation of our study. Because diabetic female patients respond better to insulin sensitizers than males^67^, further studies on the repression of the cluster in female models of diabetes and prediabetes are clearly warranted.

In summary, our data demonstrate that dysregulation of the *miR-379/miR-410* cluster in the liver is a common feature in the context of hepatic insulin resistance, both in mice and humans. The miRNAs from this cluster are highly relevant for the regulation of hepatic metabolic homeostasis in young adult animals, as liver-specific repression of the cluster induces profound metabolic effects in both sexes. Remarkably, our data also demonstrate that therapeutic targeting of the *miR-379/miR-410* cluster in the liver can greatly improve the metabolic complications in experimental models of type 1 diabetes and diet-induced, pre-diabetic obesity, to the point that both glucose and lipid homeostasis are almost normalized, making this cluster a potential druggable target for the treatment of metabolic disorders in the future.

## Methods

### Ethics statement

All experimental procedures involving animals were carried out in accordance with European Union guidelines regarding the use of animals for experimental purposes (Council Directive CEE 86/609) and were approved by the Government of Upper Bavaria (Regierungspräsidium Oberbayern; ROB-55.2-2532.Vet_02-15-164, ROB-55.2-2532.Vet_02-16-117, ROB-55.2-2532.Vet_02-21-66, ROB-55.2-2532.Vet_02-17-49). For studies involving human samples obtained from the Leipzig Obesity Biobank, all study protocols were approved by the ethics committee of the University of Leipzig in accordance with the principles of the WMA Declaration of Helsinki. For samples provided by the Tissue Bank of the National Center for Tumor Diseases (NCT) in Heidelberg, protocols were approved by the ethics committee of Heidelberg University. All participants gave written informed consent to use their data in anonymized form for research purposes before taking part in the studies.

### Vector construction

TuD inhibitors were designed following the recommendations by Haraguchi et al.^49^. Briefly, oligonucleotide pairs (Sigma-Aldrich), listed in Supplementary Table 2, were annealed and cloned into the pcDNA6.2-GW/EmGFP-miR plasmid (Invitrogen) digested with SalI and BglII restriction enzymes in order to test the activity of these inhibitors in vitro; or into the pdsAAV-LP1-EGFPmut vector^68^ also digested with SalI and BglII to be delivered into the animals via recombinant AAV vectors. The pdsAAV-LP1-EGFPmut vector includes a mutated EGFP sequence with 3 stop codons proximal to the translation starting point^68^, and a highly liver-specific artificial promoter derived from the fusion of human apolipoprotein hepatic control region and human alpha-1-antitrypsin promoter^23,52^. Recombinant AAV vectors were packaged into AAV8 capsids by Vector Biolabs (Malvern, PA).

For the generation of miRNA expressing vectors, the pri-miRNA sequence was inserted into the pcDNA6.2-GW/EmGFP-miR or pdsAAV-LP1-EGFPmut plasmids digested with SalI and BglII, as above.

To test the activity of the TuD inhibitors and the miRNA expressing vectors, reported vectors were generated by inserting a copy of perfectly complementary miRNA target site in the 3’-UTR of *Rluc* of the psiCHECK2 plasmid (Promega) digested with NotI and XhoI restriction enzymes.

### Luciferase assays

Genetically female HEK293A cells (Life Technologies, catalog #R705-07) were cultured in Dulbecco´s Modified Eagle Medium (DMEM; Gibco) supplemented with 10% FBS (Gibco), 100 IU/ml of penicillin and 100 µg/ml streptomycin (Gibco). Cells were seeded in 24-well plates at a density of 50000 cells/well one day before transfection, which was carried out using Lipofectamine 3000 (Invitrogen) according to manufacturer´s recommendations. Each well was transfected with 8 ng of psiCHECK2 reporter plasmid, 80 ng of corresponding miRNA expressing vector, and 400 ng of corresponding TuD encoding-plasmid or the backbone vector with no insert. Forty-eight h after transfection, the activity of *Renilla* and firefly luciferase was determined with the Dual Luciferase Reporter-Assay System (Promega) following the manufacturer´s protocol. *Renilla* luciferase activity values were normalized to firefly, all experiments were repeated 4 times.

### Human liver samples

Liver samples from a total of 22 individuals (5 self-reported as male and 17 self-reported as female) were obtained from the Leipzig Obesity Biobank. Five of the subjects had a BMI below 25 kg/m^2^ and were considered as controls, while the other 17 presented BMI ranging between 39–55 kg/m^2^. Out of the participants with obesity, 6 presented overt type 2 diabetes and were taking antidiabetic medications, these subjects were excluded from the analysis due to the confounder effects that the drugs would have on the metabolic parameters we intended to correlate with the expression of the miRNAs (e.g., triglycerides and HOMA-IR). Given the small number of male participants, sex was not considered in the study design. The liver biopsies were taken when the subjects presented in the clinic to undergo elective cholecystectomy, explorative laparotomy, or gastric sleeve resection, they were snap-frozen in liquid nitrogen, and stored at −80 °C for subsequent determinations. Blood samples from the same individuals were collected between 0800 and 1000 after an overnight fast, 4–5 days prior to the surgery. Plasma insulin was measured with an enzyme immunometric assay for the Immulite automated analyzer (Diagnostic Products, Los Angeles, CA). Serum triglycerides and glucose were measured as described elsewhere^69^. The homeostatic model assessment (HOMA-IR) index was calculated from fasting plasma insulin (FPI) and fasting plasma glucose (FPG) concentrations [FPI (mU/l) × FPG (mmol/l)/22.5] as originally described^70^. The subjects’ anthropometric characteristics and metabolic profile are shown in Supplementary Table 2.

#### Study of sexual differences in hepatic *miR-379* and *miR-541* levels

Human liver samples from non-diabetic lean subjects were provided by the Tissue Bank of the National Center for Tumor Diseases (NCT), Heidelberg, Germany, in accordance with the regulations of the tissue bank and the approval of the ethics committee of Heidelberg University. Samples from 24 subjects (14 males and 10 females) with an average age of 60.2 ± 2.1 years (62.7 ± 8.6 for males and 56.6 ± 11.6 for females) and BMI 23.8 ± 0.6 kg/m^2^ (24.4 ± 2.2 and 22.8 ± 3.3 for males and females, respectively) were studied. Liver samples from 42 subjects with obesity (13 males and 29 females) were obtained from the Leipzig Obesity Biobank. The cohort evaluated here had an average age of 59.2 ± 1.5 years (62 ± 9 for males and 58 ± 9 for females) and BMI 45.2 ± 0.8 kg/m^2^ (45.2 ± 3.9 and 45.3 ± 5.3 for males and females, respectively). Sex of the study participants was self-reported.

### Animal studies

Young male and female C57BL/6 J mice (6 weeks of age) were obtained from Charles River Laboratories (Sulzfeld, Germany). Female mice were used in only one study designed to evaluate the sex-dependent effects of hepatocyte-specific repression of the *miR-379/miR-410* cluster in animals fed regular chow, for the rest of the studies only male mice were used. The animals were acclimated to our animal housing facility for four weeks before taking part in the experiments, unless stated otherwise. They were housed 2-3 mice per cage and maintained with free access to tap water and standard chow (D12102C; Research diets, New Brunswick, NJ) under a 12:12-h light-dark cycle with lights on at 0600 and at controlled room temperature (20–22 °C) and humidity conditions (50 ± 15%). Cages were provided with bedding and nesting material, hiding structures and chewing implements to enhance environmental enrichment. As a general rule, samples were collected at ZT2 as a representation of a postprandial state, while food was removed at ZT3 and then samples collected at ZT9, as a representation of a fasting state. Animals were killed by cervical dislocation and tissues isolated and stored at – 80 C until analysis in all the studies. The following protocols were performed:

#### Short-term dexamethasone exposure

Over a period of 48 h, the animals received 3 ip injections ( ≈ 0.2 ml) of dexamethasone (1 mg/kg, USP grade; Sigma-Aldrich) or vehicle (2% ethanol), and they were killed 4-6 h after the last injection.

#### Long-term corticosterone exposure

Corticosterone (100 µg/ml, Sigma-Aldrich) or vehicle (0.66% ethanol) were supplemented in the drinking water for 4 weeks; animals were killed at ZT2 (samples from^71^).

#### Low-fat vs high-fat, high-sucrose feeding

C57BL/6 N mice were fed a fructose-palmitate-cholesterol diet (TD.140154; Tekland) or low-fat diet (A06071314; Research Diets, Inc) for 10 weeks.

#### Induction of diabetes by streptozotocin treatment

C57BL/6 N mice were treated with daily injections of streptozotocin (50 mg/kg, Sigma-Aldrich) or vehicle (0.1 M citric buffer, pH 4.5) for 5 consecutive days at 6 weeks of age. Body weight and blood glucose were monitored every other day and the STZ-treated mice received 3–4 injections of insulin per week (1 IU/animal) while control animals received an equal volume of saline. Experiment was terminated 2 months after induction of diabetes.

#### Circadian study

Mice were killed every 3 h over a period of 24 h, while having free access to food and water throughout the study.

#### Life cycle study

Mice were killed on postnatal day 1, at 12 weeks or at 8 months of age at ZT7 while having free access to food.

#### Hepatic-specific overexpression of miR-541

Mice were treated via tail vein injection with rAAV vectors (5 × 10^11^ genome copies/mouse) expressing either the pri-miR-541 or a negative control sequence. Animals fasted for 5-6 h were killed 4 weeks after rAAV administration.

#### Inhibition of miR-541 and miR-379 activity by antisense oligonucleotides

Animals received weekly injections of LNA-based antisense oligonucleotides against miR-541 and miR-379 coupled to a residue of triantennary N-acetylgalactosamine (GalNAc; sc, 1, 3 or 10 mg/kg doses) or scrambled control sequence for 5 consecutive weeks. Mice fasted for 5–6 h were killed 4 days after the last injection. The antisense oligonucleotides (Axolabs, sequence shown in Supplementary Table 3) were fully phosphorothioated, contained both LNA and 2´-O-methyl-ribonucleotide residues in their structure, and were bound to each other by a desoxyribonucleotide linker.

#### Hepatic-specific inhibition of miR-541 and miR-379 activity by TuD inhibitor

Mice were treated via tail vain injection with rAAV vectors (5 × 10^11^ genome copies/mouse) expressing either the TuD antimiR-541/379 or a negative control sequence. Animals fasted for 5–6 h were killed 4 weeks-3 months after rAAV administration. To study the effects of the TuD in a model of type I diabetes, diabetes was induced by STZ treatment as above and 4 weeks later the animals were administered the rAAV vectors. The study was terminated 5 weeks after rAAV administration and insulin therapy was not provided at any time. To study the effects of the TuD in the context of diet-induced obesity, 6-week-old mice were fed a high-fat, high-sucrose (HFHS) diet (45% kcal coming from fat and 20% from sucrose; D12451; Research Diets, Inc.) for 6 weeks before rAAV administration. Then, they were maintained on this diet for 5 extra weeks until the end of the study. To study whether the metabolic effects promoted by the TuD therapy were sex-dependent, both males and females were administered the rAAVs and killed 7 weeks later. To study the role of FGF21 in the metabolic effects promoted by the TuD therapy, FGF21KO animals (B6.129 Sv(Cg)-Fgf21tm1.1Djm/J^32^; Charles River Laboratories) or corresponding WT controls were treated with the rAAV vectors and killed 7 weeks later.

#### Indirect calorimetry

Mice were single housed in a climate-controlled indirect calorimetric system (TSE System, Germany). After a period of acclimatization of 24 h, food intake, locomotor activity, oxygen consumption, carbon dioxide production, energy expenditure and respiratory exchange ratio (RER) were measured every 10 min for 4–5 days. Then, body composition was determined by EchoMRI and animals were placed back in the TSE system for 5–6 more days of measurements.

#### Metabolic tests

To study glucose homeostasis, an oral (by gavage) or intraperitoneal glucose load (3 and 1-2 g/kg, respectively), intraperitoneal pyruvate injection (2 g Na-pyruvate/kg) or an intraperitoneal bolus of insulin (0.7 IU/kg; Humulin; Eli Lilly) was given to 5–6 h fasted mice (tests consistently run at ZT9). Blood glucose levels were checked by glucometer (Accu-chek, Roche) at time 0, 10, 20, 30, 45, 60, 90, 120 min during the OGTT and at 0, 15, 30, 45, 60, 90, 120 min during the other tests. Blood samples were drawn from the tail vain at time 0 and, occasionally, at other time points.

#### Euglycemic-Hyperinsulinemic Clamp

Permanent catheters (Instech Laboratories) were implanted in the right jugular vein (for infusion of substances) and left carotid artery (for blood sampling) of mice under isoflurane anesthesia. The catheters were tunneled under the skin and exteriorized at the back of the neck where they were coupled to a vascular access button (Instech Laboratories). The clamps were performed in conscious animals fasted for 5 h. Briefly, a bolus of [3-3H]glucose (27,75 kBq/min, for 2 min) was given right before a continuous infusion of [3-3H]glucose (2,78 kBq/min) that was maintained for 90 min. After that, the insulin clamp started (2,5 mU/kg/min; Humulin, Eli Lilly) and was prolonged for 120 min. Blood glucose concentrations were measured at 10 min intervals, and target glycemia ( ~ 110–120 mg/dl) was established by a variable infusion of 20% glucose containing 1,85 kBq/µl [3–3H]glucose tracer. Saline-washed erythrocytes from donor mice were also infused to compensate for the blood loss due to repeated sampling. The clamp steady-state was achieved within 60–70 min. Blood samples were then collected every 10 min for the next 40 min and processed to determine blood glucose-specific activity. To estimate insulin-stimulated glucose uptake in individual tissues, 2-deoxy-d-[1-14 C]glucose (370 kBq) was injected intravenously at the end of the clamp, and 30 min later the animals were terminated by ketamine/xylazine overdose and tissues (gastrocnemius, quadriceps femoris, epididymal and inguinal adipose tissue, interscapular brown adipose tissue and brain) isolated. Plasma [3H]- and [14 C]-radioactivity was determined in deproteinized (BaOH/ZnSO4) plasma after [3H2O] evaporation. Ra and Rd were calculated using Steele’s non-steady-state equations^72^, while endogenous glucose production (endoRa [mg/kg/min]) was determined by subtracting the GIR from total Ra. Glycolytic rates were estimated from the increment per unit of time of 3H2O multiplied by the estimated body water divided by [3-3H]glucose specific activity. 3H2O appearance was determined by linear regression of the measurements at t = 80 min to 120 min^73^. Tissue 2-[14 C]deoxyglucose-6-phosphate was extracted, and glucose uptake rates were calculated as described^74^.

#### Hormone and metabolite measurements

Insulin, FGF21, glucagon and corticosterone levels were determined by ELISA (manufactured by Alpco, R&D Systems, CrystalChem and Arbor Assays, respectively). Total and LDL cholesterol, non-esterified fatty acids (NEFA), lactate, ketone bodies, alanine and aspartate aminotransferase (ALT and AST), and alkaline phosphatase (ALP) concentrations were measured with an automated analyzer (AU480; Beckman Coulter). Plasma triglycerides and free glycerol levels were quantified with the Serum Triglyceride Determination Kit (Sigma-Aldrich). Hepatic glycogen and lipid contents were extracted and measured as previously described^75^.

#### Mitochondrial mass

For prohibitin staining, liver samples were fixed in RotiHistofix (Carl Roth), embedded in paraffin and cut into 3 µm slices. Immunohistochemical staining was then performed under standardized conditions on a Discovery XT automated stainer (Ventana Medical Systems) using rabbit anti-prohibitin (1:200; Abcam) as the primary antibody, and Discovery Universal (Ventana Medical Systems) as the secondary. For TOM20 staining, liver samples were obtained from mice perfused with 4% paraformaldehyde. Then, 6 µm cryostat sections were cut and collected on Superfrost Plus-treated slides and incubated with primary antibody against TOM20 (1:100, Abcam) and goat anti-rabbit secondary antibody (1:000, A-21428, Thermo Fischer Scientific), as well as phalloidin (1:400, A12379, Thermo Fischer Scientific) to label F-actin and DAPI (1:10000, D3571, Thermo Fischer Scientific) to label the nuclei. Only cells distinctively identified as hepatocytes were considered in the analysis.

### Primary liver culture studies

Primary hepatocytes were isolated from mice (already expressing the sequences of interest), as described^76^, with minor modifications. Briefly, under ketamine/xylazine anesthesia, the vena cava was perfused with ~100 ml of EGTA buffer (composition detailed in ref. ^77^) and the portal vein was cut to release the pressure in the liver. Then, perfusion continued with 100 ml of collagenase solution (0.9 mg/ml in high glucose DMEM supplemented with GlutaMax). Both solutions were warmed at 37 °C and supplied at a 10 ml/min flow. After perfusion, the liver was dissociated in William´s E medium (WEM) and the cell suspension filtered through a 100 µm strainer, washed, filtered again and washed a second time. Hepatocytes were seeded in collagen-coated 24-well plates at a density of 150.000 cells/well. Cells were maintained in 10% Sera Plus (Pan-Biotech) WEM with stable glutamine (Pan-Biotech), supplemented with 1% penicillin-streptomycin solution (Gibco) and 100 nM dexamethasone (Sigma-Aldrich) for 24 h. Twelve h before the assays (except for the LDL uptake), the incubation medium was changed to 0.5% Sera Plus WEM with stable glutamine, supplemented with the antibiotic cocktail but without dexamethasone. The following assays were run on hepatocytes expressing either the TuD antimir-541/379 or a negative control sequence:

### Gene expression assay

Cells were incubated for 8 h in WEM with stable glutamine and 0.5% Sera Plus, alone or containing 100 nM dexamethasone.

### Glucose production assay

Cells were incubated for 24 h in phenol-free DMEM with no glucose, supplemented with 4 mM L-glutamine, 2 mM sodium pyruvate and 20 mM sodium lactate, alone or containing 100 nM dexamethasone, 10 nM gucagon, and 10 µM forskolin, with or without 100 nM insulin.

### LDL uptake assay

Thirty-six h after seeding, hepatocytes were incubated for 2 h in WEM without serum or dexamethasone. Afterwards, medium was supplemented with Dil-LDL (2.5 µg/ml), and LDL uptake was determined at various time points in cells washed with cold PBS and fixed in 4% PFA as described^78^.

### Immunofluorescence assay for PLIN2 staining

Cells were fixed in 4% paraformaldehyde, washed and blocked in 10% horse serum. Then, incubated with primary antibody (PLIN2, 1:200; Progen) and with secondary antibody (goat anti-guinea pig, 1:1000, Thermo Fischer Scientific) and bodipy (1:1000, Thermo Fischer Scientific).

### MitoTracker green assay

Hepatocytes were plated in 96-well plates precoated with collagen at a density of 30000 cells/well. Thirty-six h after seeding, cells were incubated with 200 nM MitoTracker Green in growth medium. Results were normalized to cell number as determined by DAPI fluorescence.

### Liver fractionation

The liver was perfused and dissociated as above. During the first wash, the cell suspension was centrifuged at 40 g for 2min at 4 °C to pellet the hepatocyte fraction, while the supernatant was collected to isolate non-parenchymal cells. The process was repeated three more times on both fractions to minimize the risk of cross-contamination. To pellet the non-parenchymal cells, the supernatant was then centrifuged at 300 g for 5 min at 4 C.

#### Asessment of mitochondrial respiration

Pieces ( ≈ 100 mg) of freshly isolated liver samples were kept in ice-cold William’s E Medium (Pan Biotech) supplemented with 10% FBS (Pan Biotech) and 5% penicillin/streptomycin cocktail (Thermo Fisher Scientific) for 1–2 h before further processing. The liver pieces were then homogenized in 1 mL of ice-cold isolation buffer (250 mM mannitol, 75 mM sucrose, 100 μM K-EDTA, 10 mM KHEPES, 500 μM K-EGTA pH 7.4) using the TissueLyser (Qiagen). Tissue lysates were then centrifuged at 1000 xg for 10 min at 4 °C, supernatants collected and centrifuged two more times. After the third centrifugation, the mitochondrial pellets were washed two times in solation buffer supplemented with 0.5% fatty acid-free BSA (Sigma-Aldrich), the final mitochondrial pellet was resuspended in 100 µl of ice-cold isolation buffer without BSA for protein quantification. Samples were then diluted with ice-cold MAS buffer (250 mM mannitol, 75 mM sucrose, 100 μM K-EDTA, 10 mM KHEPES, pH 7.4) supplemented with 5 mM malate and 5 mM pyruvate, to a concentration of 12 µg / 20 µL. Then 20 µL of the mitochondria suspension was loaded on the wells of a Seahorse XF96 microplate (Agilent Technologies), the final volume per well was then completed to 180 µl adding more supplemented MAS buffer. Respiratory measurements were performed in a Seahorse XF96 Analyzer using the Seahorse XF Cell MitoStress Test kit (Agilent Technologies). The following chemicals in MAS buffer supplemented with 5 mM malate and 5 mM pyruvate were injected: ADP at port A (3.5 mM final concentration), oligomycin at port B (3.5 µM), FCCP at port C (4 µM), and rotenone (2 µM) and actinomycin A (4 µM) at port D. Mix and measure times were 0.5 and 4 min, respectively. Equal loading of mitochondria among biological replicates was further confirmed by immunoblot analysis of TOM20 and OXPHOS protein levels in the mitochondria suspensions.

#### Rna analysis

Pieces of snap-frozen hepatic human biopsies, as well as mouse liver, heart, skeletal muscle, hypothalamus, white and brown adipose tissues were homogenized in TRIzol using a TissueLyser (Qiagen, Hilden, Germany). Total RNA was then isolated from these homogenates or cell lysates using the miRNeasy Mini Kit (Qiagen), according to manufacturer’s recommendations, and then quantified by a spectrophotometric assay, obtaining purity ratios above 2.0 for all samples. Reverse transcription of 1 µg of total RNA was performed using the QuantiTect Reverse Transcription Kit (Qiagen), following manufacturer’s intructions. For the analysis of microRNAs, 10 ng of total RNA were reverse transcribed using TaqMan^®^ MiRNA Reverse Transcription Kit (Thermo Fischer Scientific). Semiquantitative real-time PCR was conducted in an Applied Biosystems® QuantStudio™ 7 Flex Real-Time PCR System (Thermo Fischer Scientific), using Taqman assays with Taqman universal master mix II, without UNG (Thermo Fischer Scientific) for the study of non-coding RNAs or with self-designed primers and PowerUp SYBR green master mix (Thermo Fischer Scientific) for the study of mRNAs. Samples were run in triplicate, data were analyzed using QuantStudio software and relative expression was determined following the 2^-ΔΔC(t)^ method^79^. The amplicons RNU48 (microRNAs Hek293A cells), RNU58B (microRNAs human liver samples), snoRNA202 (microRNAs mouse samples) and Tbp (rest of the assays) were used as internal controls. Taqman assays and primers used are listed in Supplementary Table 4 and 5, respectively.

#### Rna-seq analysis

Total RNA was extracted as above and an input of 500 ng was used to generate DNA libraries using the QuantSeq 3’ mRNA-Seq V2 Library Prep Kit (Lexogen), according to the manufacturer’s recommendation. The libraries were sequenced on an Illumina HiSeq4000 at the sequencing core facility of Helmholtz Munich. The reads were then trimmed by BBDuk^80^ and aligned to mouse genome GRCm38 using STAR 2.5.3a. Gene read quantification was performed using Rsubread. Only genes with more than 10 reads in, at least, 5 samples were included in the analysis. Differential expression analysis was performed with DESeq2 (1.38.3)^81^. *P* values were corrected for multiple testing with the Benjamini-Hochberg method and adjusted *p* value < 0.05 was considered significant. Downstream enriched pathway analyses of the significant up- and downregulated genes were run utilizing the KEGG pathway database (https://bioinformatics.sdstate.edu/go/).

#### Immunoblot analysis

Liver or skeletal muscle tissue was homogenized in RIPA buffer, supplemented with the appropriate protease inhibitor cocktail (Santa Cruz Biotechnology), using a mixer mill (Retsch). Total protein concentration was determined by Pierce BCA protein assay (Thermo Fischer Scientific), and protein lysates were then mixed with Laemmli buffer (Alfa Aesar). Twenty micrograms of protein were loaded per well and run on Novex Tris-Glycine gradient (4–12% or 8–16%, as appropriate) polyacrylamide gels (Thermo Fischer Scientific), and transferred onto nitrocellulose membranes (0.2–0.45 µm pore size, as appropriate) using the Trans-Blot Turbo transfer system (Bio-Rad). Membranes were blocked for 1 h in 5% skim-milk solution in Tris-buffered saline with 0.1% Tween-20 (TBST), and incubated overnight with the corresponding antibody (listed in Supplementary Table 6) in 3% BSA solution in TBST at 4 C (1:1000 dilution), following by incubation with anti-rabbit IgG secondary antibodies (Bio-Rad) at room temperature (1:10000 dilution). All washes were done with TBST. The membranes were imaged with Amersham ECL prime detection reagent (Cytiva) on a ChemiDoc imaging system (Bio-Rad).

#### Proteomic analysis

Fifteen liver samples (7 AAV-NC and 8 AAV-TuD) were homogenized in RIPA buffer as above. Ten µg of protein per sample were digested with Lys-C and trypsin following the filter-aided sample preparation procedure, as described^82^. Briefly, the proteins were reduced using dithiothreitol and alkylated using iodoacetamide in solution prior to dilution with 8 M urea buffer to reach a final urea concentration >4 M. Denatured Proteins were centrifuged on a 30kD cutoff filter (Sartorius), washed three times with 8 M urea buffer and three times with 50 mM ammonium bicarbonate followed by over-night digestion using Lys-C (Wako Chemicals) and trypsin (Promega). Tryptic peptides were collected by centrifugation and the resulting peptide solution was acidified with trifluoroacetic acid and then loaded into a Q Exactive HF-X mass spectrometer (Thermo Fisher Scientific) coupled to the Dionex UltiMate 3000 RSLCnano HPLC Systerm (Thermo Fischer Scientific), as described in data-dependent acquisition mode^83^. Tryptic peptides were trapped on a C18 pre column (300 µm inner diameter (ID) × 5 mm, Acclaim PepMap100 C18, 5 µm, 100 Å, LC Packings) at 30 µl/min flow rate followed by reversed-phase separation on a C18 analytical column (nanoEase MZ HSS T3 Column, 100 Å, 1.8 µm, 75 µm × 250 mm, Waters) at 250 nl/min flow rate in a 95 min nonlinear acetonitrile gradient from 3 to 40% in 0.1% formic acid. The precursor spectrum was acquired at a resolution of 60,000 (full width at half-maximum) with a mass range from 300–1500 m/z with automatic gain control target set to 3 × 10E6 and a maximum injection time of 30 ms. From the MS prescan, the 15 most abundant peptide ions were selected for fragmentation (MSMS spectrum) if they were at least doubly charged, with a dynamic exclusion of 30 s. MSMS spectra were recorded at a resolution of 15,000 with automatic gain control target set to 5 × 10E2 and a maximum injection time of 50 ms. The normalized collision energy was 28.

Raw data was analyzed using the Proteome Discoverer software 2.4 (Thermo Fischer Scientific) for protein quantification and identification via database search (Sequest HT search engine) against the SwissProt Mouse database (release 2020_02, 17601 sequences), considering full tryptic specificity, and one missed tryptic cleavage site was allowed with a minimum peptide length of 6 amino acids. Precursor mass tolerance was set to 10 ppm and fragment mass tolerance to 0.02 Da. Carbamidomethylation of C was set as a static modification. Dynamic modifications included deamidation of N and Q, oxidation of M; and a combination of Met loss with acetylation on protein N-terminus. Quantification of proteins was based on intensity values (at RT apex) for all unique peptides per protein with a XCorr score >1 and a peptide false discovery rate of <1% using the percolator node. Match between runs was enabled within the Minora Feature Detection node with 1 ppm mass tolerance and 0.5 min retention time tolerance. Peptide abundance was normalized to total amount. Missing values were imputed by low abundance resampling separately for each sample within Proteome Discoverer 2.4. All unique peptide abundances were used for calculation of normalized protein abundances. Protein ratios between the two groups were calculated through a pairwise ratio calculation using the Precursor Ions Quantifier node, taking the geometric median of all unique peptide-level replicate combinations. A background-based *t* test with FDR correction (adjusted *p* values) was used as hypothesis test. Protein identifications and quantifications were exported and filtered for a protein false discovery rate of <5%. Gene set enrichment analysis was conducted using the GSEA software developed by the UC San Diego and the Broad Institute^84^.

#### Metabolomic analysis

Fragments ( ≈ 20 mg) of 15 liver samples (7 AAV-NC and 8 AAV-TuD) were extracted in 100% methanol solution (190 µl) containing ribitol (0.02 mg/ml) at 70 °C with vigorous shaking for 15 min. Then, 100 µl of a 100% chloroform solution was added, and the samples were shaken at 37 °C for 5 extra minutes. Polar and organic phases were separated by centrifugation (11000*g* for 10 min) after the addition of 200µL HPLC-grade water. The polar upper phase was then transferred to a fresh vial and dried using the Eppendorf Concentrator Plus.

Sequential methoximation and silylation reactions were performed using a MPS autosampler (Gerstel, Mülheim Ruhr). Methoximation was conducted by adding 20 µL 20 mg/ml methoxyamine hydrochloride (Sigma 226904) in pyridine (Sigma 270970) and incubation at 37 °C for 90 min in an Gerstel MPS Agitator Unit (250 rpm). For silylation reactions, 45 µl of N-Methyl-N-(trimethylsilyl)trifluoroacetamide (MSTFA; Sigma 69479) were added and samples were incubated at 37 °C for 30 min with gentle shaking. Before injection, samples were incubated at RT for 45 min. For gas chromatography/mass spectrometry (GC/MS) the samples were loaded into a GC-ToF system. This system included an Agilent 7890 Gas Chromatograph (Agilent, Santa Clara) fitted with a Rxi-5Sil MS column (Restek Corporation, Bellefonte) coupled to a Pegasus BT Mass Spectrometer (LECO Corporation, Michigan). The GC was operated with an injection temperature of 250 °C and 1 µL sample was injected in splitless mode. The GC temperature program started with a 1 min. hold at 40 °C followed by a 6 °C/min ramp up to 210 °C, a 20 °C/min ramp up to 330 °C and a bake-out at 330 °C for 5 min. using Helium as carrier gas with constant linear velocity. The ToF mass spectrometer was operated with ion source and interface temperatures of 250 °C, a solvent cut time of 9 min and a scan range (m/z) of 50–600 with an acquisition rate of 17 spectra/second.

The ChromaTof v5.50 software (LECO Corporation, Michigan) was used for data processing. The resulting signal intensities (peak area) were transformed and analyzed using Perseus software^85^.

#### Statistical analysis

Data are expressed as the mean ± SE. Areas under the curves (AUC) were calculated by the trapezoid rule. Matsuda’s composite index was calculated as previously described^86^. Distance correlation, a measure of statistical dependence that captures both linear and non-linear–including non-monotonic–relationships, was calculated to assess associations between variables^87^. In contrast to Pearson’s correlation, distance correlation equals zero if and only if the variables are statistically independent, making it particularly suitable for quantifying complex relationships in biological data. Calculations were performed using the *energy* package in R version 4.4.2 (R Core Team, ^88^). Two-tailed Student’s *t* tests were conducted to compare two independent groups; while one-way or two-way ANOVA, as appropriate, to compare three or more groups. Homocedasticity of the data was checked by the Bartlett’s test and a Holm-Sidak multiple comparison test was run for post hoc analysis. Significance was set at p ≤ 0.05. GraphPad Prism 9 software was used to perform the statistical analysis. Nonlinear curve fitting calculation was performed using OriginPro 2024 software. ANCOVA/generalized linear model using lean body mass as covariate was calculated by CalR^89^ to compare data on food intake, energy expenditure, oxygen consumption and carbon dioxide production collected by the TSE’s PhenoMaster system. For the analysis of transcriptomic and proteomic data, changes with a false discovery rate ≤0.05 were considered significant. While, for the analysis of metabolomic data, changes with a false discovery rate ≤0.1 were considered significant.

### Reporting summary

Further information on research design is available in the Nature Portfolio Reporting Summary linked to this article.

## Supplementary information

Supplementary information

Reporting Summary

Transparent Peer Review file

## Source data

Source Data

## Data Availability

All data generated or analyzed during this study are included in this manuscript (and its supplementary information and Source Data files). The mass spectrometry proteomics data have been deposited to the ProteomeXchange Consortium via the PRIDE partner repository with the dataset identifier PXD044478. The complete transcriptomic datasets were deposited to the National Center for Biotechnology Information’s Gene Expression Omnibus Database and are accessible through GEO Series accession number GSE304566. Metabolomics data are available at Mendeley data (https://data.mendeley.com/datasets/76zzw9gd4b/1). Source data are provided as Source Data files. Source data are provided with this paper.
