# Supplementary material for: Combinatorial targeting of the miR-379/miR-410 cluster normalizes glucose and lipid homeostasis in male models of diabetes and obesity
Source: Nat Commun. 2026 Sep 21;17:10014. doi: 10.1038/s41467-026-77808-2 (PMC13594115; doi:10.1038/s41467-026-77808-2)
Supplement: Supplementary file 1 [file 41467_2026_77808_MOESM1_ESM.pdf]

SUPPLEMENTARY INFORMATION

Table of Contents:

Supplementary Figure Legends 3

Supplementary Figure 1..12

Supplementary Figure 2 13

Supplementary Figure 3..14

Supplementary Figure 4..15

Supplementary Figure 5 16

Supplementary Figure 6 17

Supplementary Figure 7 18

Supplementary Figure 8 19

Supplementary Figure 9 20

Supplementary Figure 10 21

Supplementary Figure 11 22

Supplementary Figure 12 23

Supplementary Figure 13 24

Supplementary Figure 14 25

Supplementary Figure 15 26

Supplementary Figure 16 27

Supplementary Figure 17 28

Supplementary Figure 18 29

Supplementary Table Legends 30

Supplementary Table 1 31

Supplementary Table 2 32

Supplementary Table 3 33

Supplementary Table 4 34

Supplementary Table 5 35

Supplementary Table 6 36

SUPPLEMENTARY FIGURE LEGENDS

Supplemental Figure 1: **A**-Levels of expression of different miRNAs from the DLK1-DIO3 locus in the liver of human subjects with BMI<30 (n=5) vs. BMI>30 (n=11). Absence of sex differences in the hepatic levels of expression of *miR-379* (**B**) and *miR-541* (**C**) of both lean subjects and subjects with obesity (Lean male, n=14; lean female, n=10; male with obesity, n=13; female with obesity, n=29). Data are expressed as mean ± SEM. Levels of expression of the miRNAs were compared by two-tailed Student's t test in 1A and two-way ANOVA, followed by Holm-Sidak multiple comparison test, in 1B and 1C; **P<0.01; ***P<0.001 vs. control.

Supplemental Figure 2: Profile of expression of *miR-379* in different metabolic tissues of male mice in response to short-term (48 h) dexamethasone (**A**, n=6) and long-term (4 wks) corticosterone (**B**) exposure (Vehicle-treated groups, n=9; corticosterone-treated liver and BAT, n=10; corticosterone-treated eWAT, n=8). Profile of expression of *miR-541* in different metabolic tissues of male mice in response to short-term (48 h) dexamethasone (**C**, n=6)t and long-term (4 wks) corticosterone (**D**) exposure (Vehicle-treated groups and corticosterone-treated liver, n=9; corticosterone-treated eWAT, n=8; corticosterone-treated BAT, n=10; **eWAT**=epididymal adipose tissue; **scWAT**=subcutaneous, inguinal, adipose tissue; **BAT**=brown adipose tissue; **SM**=skeletal muscle). Levels of expression of hepatocyte (**E**) and non-parenchymal cells (**F**) markers, as well as non-coding RNAs from the miR-379/miR-410 (**G**) and the miR-127/miR-136 (**H**) clusters in hepatocyte and non-parenchymal fractions of livers isolated from male mice previously treated with dexamethasone for 48 h (n=3 animals/group). Data are expressed as mean ± SEM. Results were anaylsed by two-tailed Student's t test in 2A-D, and by two-way ANOVA followed by Holm-Sidak multiple comparison test in 2E-H; *P<0.05; **P<0.01; ***P<0.0001 vs. respective control.

Supplemental Figure 3: Daily variation in the mRNA levels of two canonical clock genes (*Bmal1* and *Per2*; **A**) as well as in the levels of expression of the host transcript *Mirg* (**B**) and the mature miRNAs, *miR-379* and *miR-541* (**C**) from the *miR-379/miR-410* cluster, in the liver

of male mice with ad libitum access to food (n=3 mice/time point). Data are double plotted and separated by a broken line. The insets in each graph show the adjusted R-Square and the p-value of fitting the daily profile of expression to a waveform function with a 24 h-period. **D**-Levels of expression of the host transcripts, *lncMGC* and *Mirg*, as well as the mature miRNAs, *miR-379* and *miR-541*, from the *miR-379/miR-410* cluster in liver samples isolated on postnatal day 1 (P1; n=7), as well as from male animals with 12 wks (n=6) and 8 months of age (n=6). mRNA levels of two markers of ER stress, *Ddit3* (CHOP) and *Atf4*, in the liver of male animals treated with dexamethasone injections for 48 h vs. vehicle (**E**; n=6), supplemented with corticosterone in the drinking water for 4 wks (n=10= vs. vehicle (**F**; n=9) and male mice treated with STZ at 6 wks of age and then maintained with insulin therapy for 6 months (n=10) vs. vehicle (**G**; n=9). Data are expressed as mean ± SEM. Two-tailed Student's t tests were used to analyse the data in 3E-G; *P<0.05; **P<0.01; ***P<0.001 vs. respective control.

Supplemental Figure 4: Metabolic effects in response to hepatic-specific overexpression of *miR-541* and simultaneous targeting of *miR-379* and *miR-541* by antisense oligonucleotides. Levels of expression of *miR-541* and other components of the *miR-379/miR-410* cluster in the liver (**A**) and epididymal adipose tissue (**B**) of male mice with hepatic-specific overexpression of *miR-541* pre-miR sequence (n=5). Fasting plasma insulin levels (**C**) and corresponding HOMA-IR values (**D**), as well as morning-fed blood glucose (**E**) and triglyceride concentrations (**F**) of the same animals. Levels of expression of *miR-379* (**G**) and *miR-541* (**H**) in the liver of male animals receiving LNA-based ASOS against miR-379 and miR-541, coupled to GalNAc, at different doses for 5 wks (1 mg/kg dose, n=14; 3 and 10 mg/kg doses, n=6). **I**-Hepatic levels of expression of different components of the miR-379/miR-410 cluster in male animals receiving the same LNA-based ASOS therapy at dosage of 1 mg/kg for 5 weeks (*miR-379* and *Mirg,* n=14; *miR-541*, n=13; remaining non-coding RNAs, n=6). **J**-Levels of expression of the host transcript lncMGC in the liver of male animals receiving LNA-based ASOS against miR-379 and miR-541, coupled to GalNAc, at different doses for 5 wks

(n=6). Fasting plasma triglyceride levels (**K**; n=14), time course profile of the glucose response to an exogenous insulin bolus (0.7 IU/kg) and corresponding AUC (**L**; n=12), and HOMA-IR values (**M**; n=14) in male mice receiving the same LNA-based ASOS therapy coupled to GalNAc at dosage of 1 mg/kg for 5 wks. Data are expressed as mean ± SEM. Two-tailed Student's t tests were run to analyse the results; *P<0.05, **P<0.01, ***P<0.001 vs. respective control.

Supplemental Figure 5: Relative *Renilla* to Firefly luciferase ratio in HEK293A cells in culture transfected with a reporter vector carrying a *miR-379* target site in the 3'-UTR of the Renilla gene; a second vector expressing *miR-379* or miR-NC; and a third vector expressing a TuD-NC, a TuD antimiR-379 (**A**) or a TuD antimiR-541/miR-379 (**B**) sequence (n=4 independent experiments). **C**: Levels of expression of *miR-379* in HEK293A cells transfected with a vector overexpressing *miR-379* and a second vector expressing one of the TuD sequences indicated in the X axis (n=3 independent experiments). **D-E**: Same as in A and B, but this time the reported vector carries a *miR-541* target site, the second vector expresses *miR-541* or miR-NC, and the third vector TuD-NC, TuD-antimiR-541 or TuD antimiR-541/miR-379 (n=4 independent experiments). **F**: Same as in C, but this time the levels of expression of *miR-541* are reported and, accordingly, the first vector overexpresses that miRNA sequence (n=3 independent experiments). Data are expressed as mean ± SEM. Two-tailed Student's t tests were run to compare the groups in 5A-B and 5D-E, while one-way ANOVA followed by Holm-Sidak multiple comparison test was used in 5C and 5F; *P<0.05; **P<0.01; ***P<0.001 vs. respective control.

Supplemental Figure 6: **A**-Relative mRNA levels of *EGFP* in liver, epidydimal fat, or heart of male mice receiving rAAV-TuD therapy or treated with PBS; *EGFP* is transcribed but not translated in the cells expressing the rAAV vector (PBS-treated liver and TuD-treated liver for 3 wks, n=6; remaining groups, n=12). **B**-Profile of expression of *Mirg* in extrahepatic tissues of male mice receiving rAAVs and expressing the tough decoy inhibitor (AAV-TuD) or a negative control sequence (AAV-NC; **eWAT**=epididymal adipose tissue;

scWAT=subcutaneous, inguinal, adipose tissue; **BAT**=brown adipose tissue; **SM**=skeletal muscle; **Hyp**=hypothalamus; eWAT and heart, n=12; BAT AAV-NC, n=4; BAT AAV-TuD, n=6; SM AAV-NC, n=11; SM AAV-TuD, n=10; Hyp AAV-NC, n=7; Hyp AAV-TuD, n=8). Induction of miR-541 (**C**) and Mirg (**D**) expression by dexamethasone incubation in male primary hepatocytes in culture expressing a NC sequence, and abrogation of this effect in the cells expressing the TuD sequence (hepatocytes expressing NC, n=5; hepatocytes expressing TuD, n=3). Immunoblots showing upregulation of RICTOR protein levels in the liver of male animals with hepatic-specific repression of the *miR-379/miR-410* cluster for 5 weeks (**E**) and 3 months (**F**, n=6). Upregulation of hepatic *Atf3* mRNA levels following the liver-specific silencing of the *miR-379/miR-410* cluster in male regular chow-fed animals under different feeding conditions (**G**; n=6) as well as in male diet-induced obese animals (**H**; chow+AAV-TuD, n=12; remaining groups, n=11). **I**-Upregulation of *Dlk1* mRNA levels in the liver, but not in the skeletal muscle (gastrocnemius) or epidydimal fat of male animals with hepatic-specific repression of the *miR-379/miR-410* cluster, (liver and eWAT; n=12; SM, n=11). Data are expressed as mean ± SEM. Two-tailed Student's t tests were run to compare the groups in 6B, 6E-F and 6I, while two-way ANOVA followed by Holm-Sidak multiple comparison test was used to analyse the data in 6C-D and 6G-H; *P<0.05; **P<0.01; ***P<0.001 vs. respective control.

Supplemental Figure 7: **A**-Change in body weight over a 13-week period in male animals expressing the rAAV vectors (TuD vs. NC sequence; AAV-NC, n=12; AAV-TuD, n=11). Cumulative food (**B**, n=4 cages) and water intake (**C**, n=4 cages), and change in body composition (**D**; AAV-NC, n=12, AAV-TuD, n=11), as determined by EchoMRI, in the same animals over the same period of time. Circulating levels of ALT (**E**), AST (**F**), ALP (**G**) and creatinine (**H**) in male animals on rAAV therapy (NC vs. TuD) at 5 weeks and 3 months after rAAV administration (AAV-NC 5 wks, n=8; AAV-TuD 5 wks, n=7; AAV-NC 3 mos, n=12; AAV-TuD 3 mos, n=11). **G**-Inflammation score (based on number of foci per 200X field of view) of male animals expressing the rAAV vectors (TuD or NC sequence) for 3 months. Data are

expressed as mean ± SEM. Two-way ANOVA followed by Holm-Sidak multiple comparison test was used to compare groups in 7A-C, while two-tailed Student's t tests were run to analyse data in 7D-F.

Supplemental Figure 8: Hourly profile of food intake (**A**), locomotor activity (**B**), energy expenditure (**C**), oxygen consumption (**D**), carbon dioxide production (**E**), and respiratory exchange ratio (**F**) over a period of 4 consecutive days in male mice expressing the tough decoy inhibitor (AAV-TuD) or a negative control sequence (AAV-NC) in the liver, and which were individually housed in the TSE's PhenoMaster system (n=14). Data are expressed as mean ± SEM. **G**-Results of the ANCOVA/generalized linear model of data presented in A, C, D and E with lean body mass as covariate. **H**-Results of the one-way ANOVA of data presented in B and F.

Supplemental Figure 9: Hepatic triglyceride content (**A**) and percentage of the cellular area occupied by lipids in liver sections (**B**) of male animals receiving rAAVs therapy and expressing either the tough decoy inhibitor (AAV-TuD) or a negative control sequence (AAV-NC) and maintained on regular chow or a high-fat, high-sucrose diet (HFHS; HFHS+AAV-TuD, n=11; remaining groups, n=12). Upregulation of *Lpl*, *Cd36* and *Lipa* mRNA levels in the liver of male animals with hepatic-specific repression of the *miR-379/miR-410* cluster maintained for 5 weeks (**C**; n=12) and 3 months (**D**; AAV-NC, n=12; AAV-TuD, n=11). Data are expressed as mean ± SEM. Two-way ANOVA followed by Holm-Sidak multiple comparison test were run to compare groups in 9A-B, while two-tailed Student's t tests were used to analyse data in 9C-D; ***P<0.001 vs. respective control. **E**: List of miRNAs belonging to *the miR-379/miR-410* cluster predicted by miRwalk to interact with *Lpl*, *Cd36* and *Lipa*. It is specified the region of the gene that the miRNAs are predicted to bind; 5'UTR, 5' untranslated region; CDS, coding sequence; 3'UTR, 3' untranslated region.

Supplemental Figure 10: Plasma lactate (**A**) and glycerol (**B**) levels in morning-fed conditions and after 7 and 14 h of fasting in male animals receiving AAV-NC or AAV-TuD therapy (n=7; the shadowed area indicates the dark period). Induction of *Pck1* (**C**) and *G6pc* (**D**) mRNA

levels by dexamethasone incubation in male primary hepatocytes in culture expressing a NC sequence (n=5), and abrogation of this effect in cells expressing the TuD sequence (n=3). **E**-Time course profile of Δ blood glucose concentrations following a pyruvate tolerance test (2 g/kg) 5 wks after rAAV administration and corresponding AUC in both male and female mice (female+AAV-TuD, n=12; female+AAV-NC, n=11; male+AAV-NC, n=10; male+AAV-TuD, n=9). **F**: Hepatic glycogen content after a short period of fasting (6 h) in male mice with hepatic-specific silencing of the *miR-379/miR-410* cluster (n=6). **G**: Time course profile of plasma insulin levels in response to an oral glucose load (3 g/kg) in male animals receiving AAV-NC (n=12) or AAV-TuD therapy (n=8), 4 weeks after rAAV administration. **H**: Short-term fasting (6 h) plasma insulin concentrations at different times in male mice after rAAV administration (5 wks, n=6; remaining time points, n=12, insulin levels are missing in one animal from the AAV-TuD wk2 group due to limited sample volume). Plasma corticosterone levels at the peak (ZT9) and trough (ZT17) of the endogenous daily rhythm (**I**; n=6) and plasma glucagon levels in response to a 10 h fasting (**J**; AAV-NC, n=12; AAV-TuD, n=9) in male animals with hepatic-specific silencing of the *miR-379/miR-410* cluster. Basal and clamp plasma insulin levels (**K**), as well as blood glucose concentrations (**L**) achieved during the steady state of the hyperinsulinemic-euglycemic clamp presented in Fig.5G (n=8). **M**: Upregulation of *Fgf21* mRNA levels in the liver of male mice with hepatic-specific repression of the *miR-379/miR-410* cluster (AAV-NC 3 mos, n=12; AAV-TuD 3 mos, n=11; remaining groups, n=6). **N**-Circulating levels of FGF21 in male FGF21KO and WT mice with hepatic-specific silencing of the *miR-379/miR-410* cluster (WT+AAV-NC, n=9; remaining groups, n=10). **O**-Plasma concentrations of triglycerides (left panel) and LDL cholesterol (right panel) in male FGF21KO and WT receiving rAAVs expressing either the tough decoy (TuD) or a negative control sequence (NC; n=10). **P**-Time course profile of blood glucose levels in response to an ipGTT (2 g/kg) in the same animals as in O, 4 wks after rAAV administration (n=10). **Q**-HOMA-IR values over time in male WT and FGF21KO mice treated with rAAVs expressing the tough decoy inhibitor (n=10). Data are expressed as mean ± SEM. Two-way ANOVA followed by Holm-Sidak multiple comparison test was used to analyse the data in 10A-E, 10G-I, 10K-L,

and 10N-P. Two-tailed Student's t tests were run to compare groups in 10F, 10J, 10M, and 10Q; *P<0.05; **P<0.01; ***P<0.001 vs. respective control.

Supplemental Figure 11: Pathway enrichment analysis of genes predicted to be targeted by miR-379 (**A**), miR-541 (**B**), miR-382 (**C**), miR-134 (**D**) and miR-409 (**E**). Potential targets of each miRNA were predicted by miRNA target prediction algorithms (http://mirwalk.umm.uni-heidelberg.de/). Only genes predicted to be targeted by the same microRNA at multiple sites were considered for the enriched pathway analysis, which was run utilizing the KEGG pathway database (https://bioinformatics.sdstate.edu/go/). The 20 significantly enriched pathways for each microRNA are shown in the figure.

Supplemental Figure 12: Principal component analysis of the RNA-seq data (**A**), as well as heatmap and hierarchical clustering (**B**), and volcano plot representation (**C**) of the differentially expressed genes in the livers of male animals receiving rAAV therapy and expressing either the tough decoy (TuD, n=6) or a negative control sequence (NC, n=5). Enrichment pathway analysis of the significantly upregulated and downregulated genes in TuD-treated animals was run utilizing the KEGG pathway database, top upregulated (**D**) and downregulated (**E**) pathways were ranked based on false discovery rate (FDR) after filtering out pathways unrelated to liver tissue.

Supplemental Figure 13: Principal component analysis of the proteomics data (**A**), as well as heatmap and hierarchical clustering (**B**), and volcano plot representation (**C**) of the proteins differentially regulated in the livers of male animals receiving rAAV therapy and expressing either the tough decoy (TuD, n=8), or a negative control sequence (NC, n=7). Pathways among the top upregulated (**D**) and downregulated (**E**), as determined by GSEA, in the liver of male animals expressing AAV-TuD as compared to AAV-NC. ES, enrichment score; NES, normalized enrichment score; FDR, false discovery rate. **F**-Heatmap showing the relative abundance of GKAP1 in the liver, as determined by the proteomic analysis. **G**-List of miRNAs belonging to *the miR-379/miR-410* cluster predicted by miRwalk to bind *Gkap1*, the miRNAs for which there is proof of direct repression in response to the TuD therapy are highlighted in

bold. It is specified the region of the gene that the miRNAs are predicted to bind; 5'UTR, 5' untranslated region; CDS, coding sequence; 3'UTR, 3' untranslated region.

Supplemental Figure 14: Immunoblots to study PI3K-mTOR signaling activation; including total and phosphorylated-AKT at Thr308 residue (**A**); total and phosphorylated-AKT at Ser473 residue (**B**); total and phosphorylated-FOXO1 at Ser256 residue (**C**); RICTOR (**D**); total and phosphorylated-4EBP1 at residues Thr37 and Thr46 (**E**); and total and phosphorylated-RPS6 at residues Ser240 and Ser244 (**F**) in the skeletal muscle (gastrocnemius) of male animals receiving AAV-NC or AAV-TuD therapy (n=6), and corresponding densitometric quantification. Data are expressed as mean ± SEM. Two-tailed Student's t tests were run to analyse the densitometric results; *P<0.05 vs. control.

Supplemental Figure 15: **A-**Relative abundance of metabolites found to be significantly enriched in the liver of male animals expressing the TuD (n=8) as compared to negative control (n=7). **B-** Relative abundance of glucose and lactic acid, as determined by metabolomic analysis, in the liver of the same animals as above. Data are expressed as mean ± SEM. False discovery rate was calculated using Perseus software; P=FDR-adjusted p-value.

Supplemental Figure 16: **A-**Relative abundance of the top upregulated mitochondrial proteins in the liver of male mice receiving rAAV-TuD therapy as compared (n=8) to NC (n=7). **B-**mRNA levels of *Cyptb1b* and *Ucp2* in the liver of the same animals as in A. **C**-Ponceau staining of the immunoblot to study the abundance of the different mitochondrial complexes (**D**) in aliquots of mitochondrial extracts, isolated from the liver of male animals on AAV-TuD or AAV-NC therapy (n=7), that were then seeded in a Seahorse plate to study mitochondrial oxygen consumption. **E**-Densitometric quantification of the immunoblot shown in panel D (n=7). **F-**Heatmap showing the relative abundance of pyruvate carboxylase (PC) and pyruvate dehydrogenase kinase (PDK) 1 and 2 in the same liver samples as in A, as determined by proteomic analysis. Data are expressed as mean ± SEM. Two-tailed Student's t tests were run to compare the qPCR and densitometric results; *P<0.05 vs. respective control.

Supplemental Figure 17: **A**-Hepatic mRNA levels of G6pc and Pck1 in male STZ-treated mice on either AAV-TuD (n=9) or AAV-NC therapy (n=8). **B**: α- and β-cell mass, as well as alpha-to-beta cell ration in the pancreatic islets of male STZ-induced diabetic mice receiving either AAV-NC or AAV-TuD therapy and corresponding vehicle-treated non-diabetic controls (Veh+AAV-NC, n=10; Veh+AAV-TuD, n=7; STZ+AAV-NC, n=8; STZ+AAV-TuD, n=9). Serum insulin levels at termination (**C**); cumulative water intake throughout the entire duration of the experiment (9 wks, **D**; Veh+AAV-TuD, n=3 cages; STZ+AAV-NC, n=4 cages; remaining groups, n=5 cages); serum NEFA levels (**E**); and plasma triglyceride concentrations (**F**) of the same animals as in B. Data are expressed as mean ± SEM. Two-tailed Student's t test was used to compare groups in 17A, while two-way ANOVA followed by Holm-Sidak multiple comparison test was run to analyse data in 17B-F; *P<0.05; **P<0.01; ***P<0.001 vs. respective controls.

Supplemental Figure 18: Plasma triglyceride concentrations at different feeding conditions (**A**) and adipose tissue mass at termination (**B**) of male diet-induced obese animals receiving either AAV-NC or AAV-TuD therapy and corresponding regular chow-fed non-obese controls (HFHS+AAV-TuD, n=11; remaining groups, n=12). **C**-Hepatic mRNA levels of G6pc and Pck1 in male diet-induced obese animals on either AAV-TuD or AAV-NC therapy (n=6). **D**-Change in body weight over a 5-week period and average daily caloric intake (**E**) of male diet-induced obese animals and corresponding regular chow-fed non-obese controls receiving rAAV vectors and expressing either the tough decoy inhibitor or a negative control sequence (HFHS+AAV-TuD, n=11; remaining groups, n=12; for average daily caloric intake, n=4 cages). **F**-Circulating levels of ALT and AST at termination and inflammation score (**G**, based on number of foci per 200X field of view) in male diet-induced obese animals on either AAV-TuD (n=11) or AAV-NC therapy (n=12). Data are expressed as mean ± SEM. Two-way ANOVA followed by Holm-Sidak multiple comparison test was used to analyse data in 18A-B and 18D-E, while two-tailed Student's t test was used to compare data in 18C and 18F; *P<0.05; **P<0.01; ***P<0.001 vs respective controls.

Supplemental Figure 1.

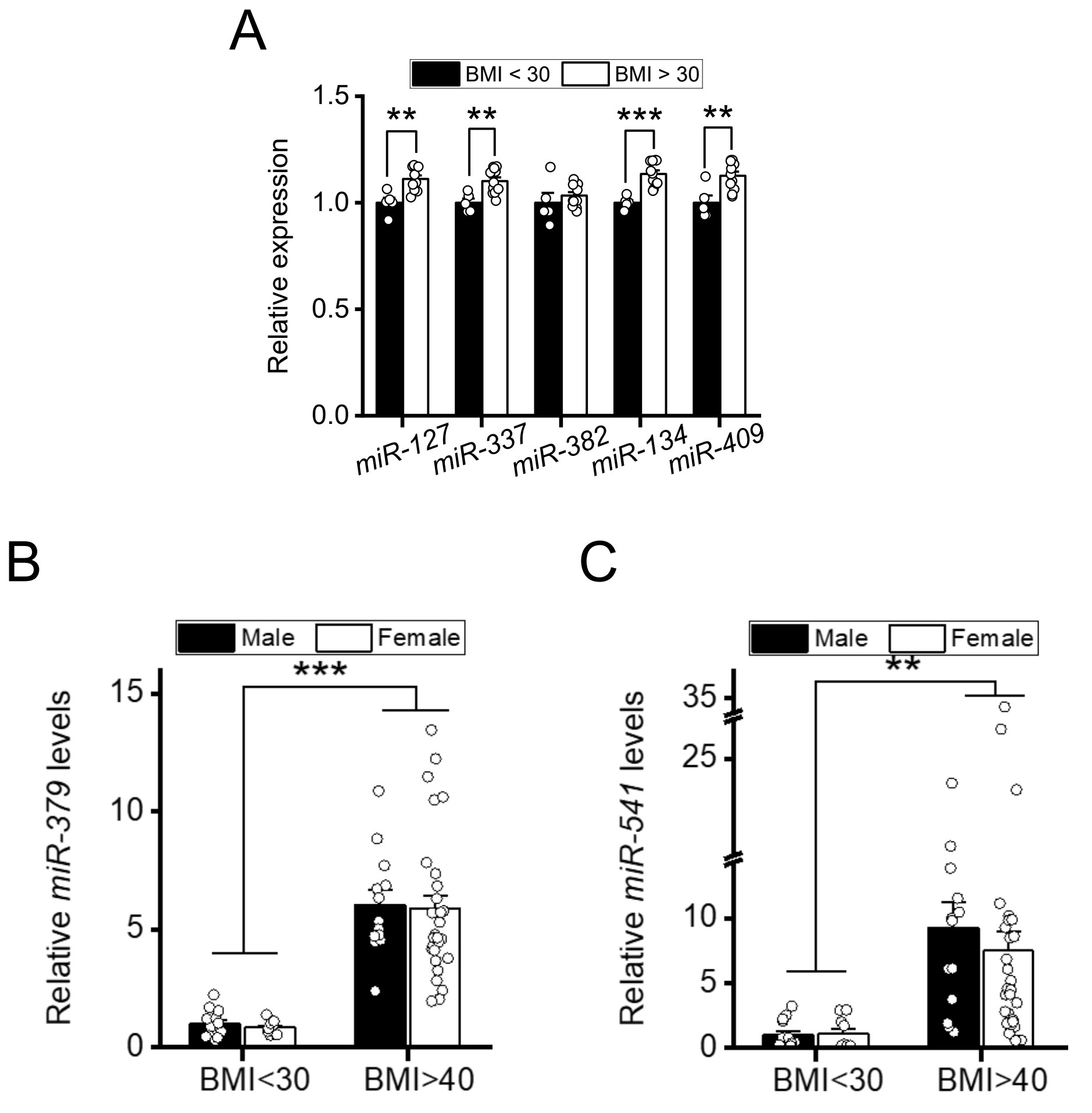

Supplemental Figure 2.

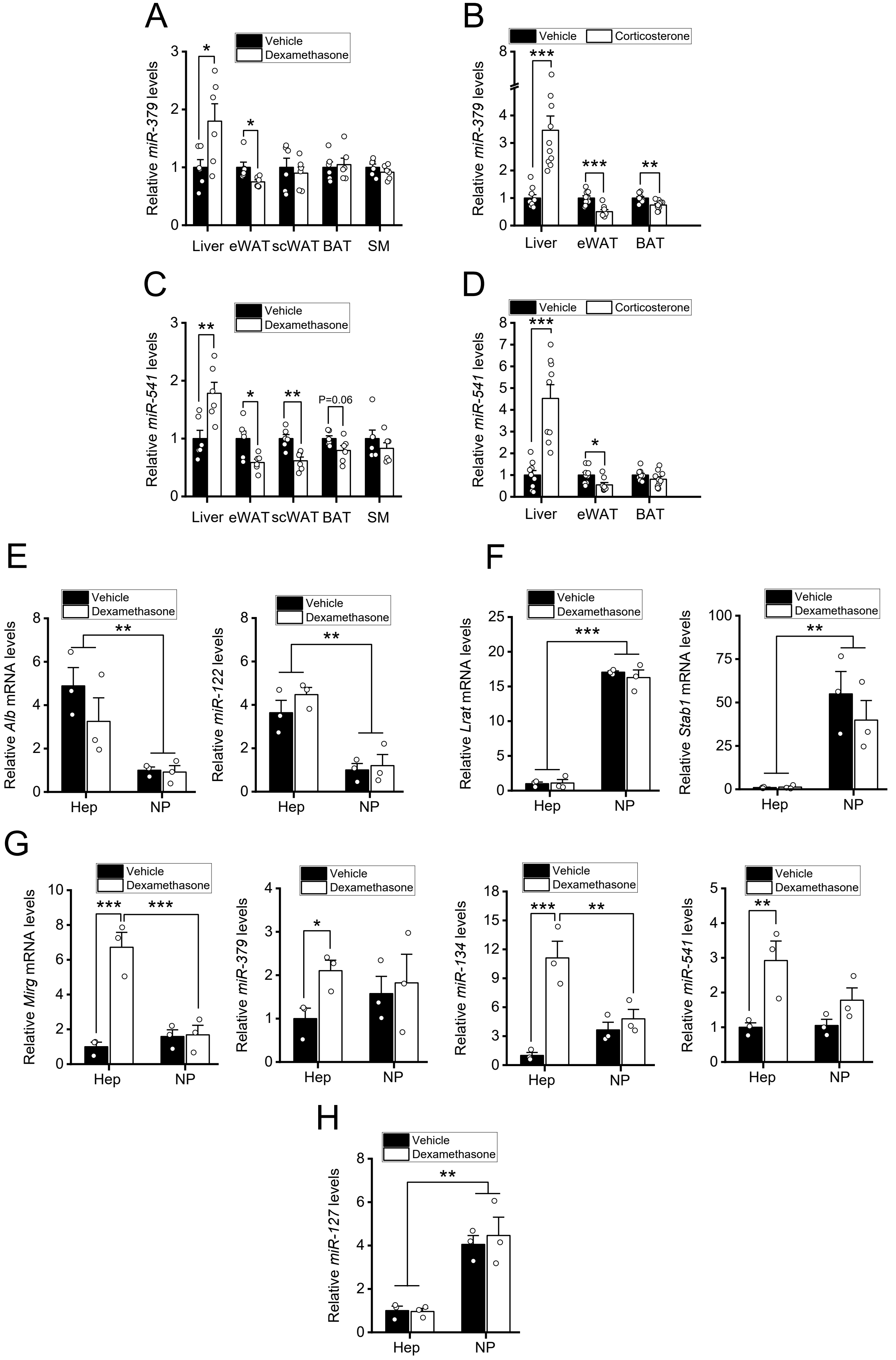

Supplemental Figure 3.

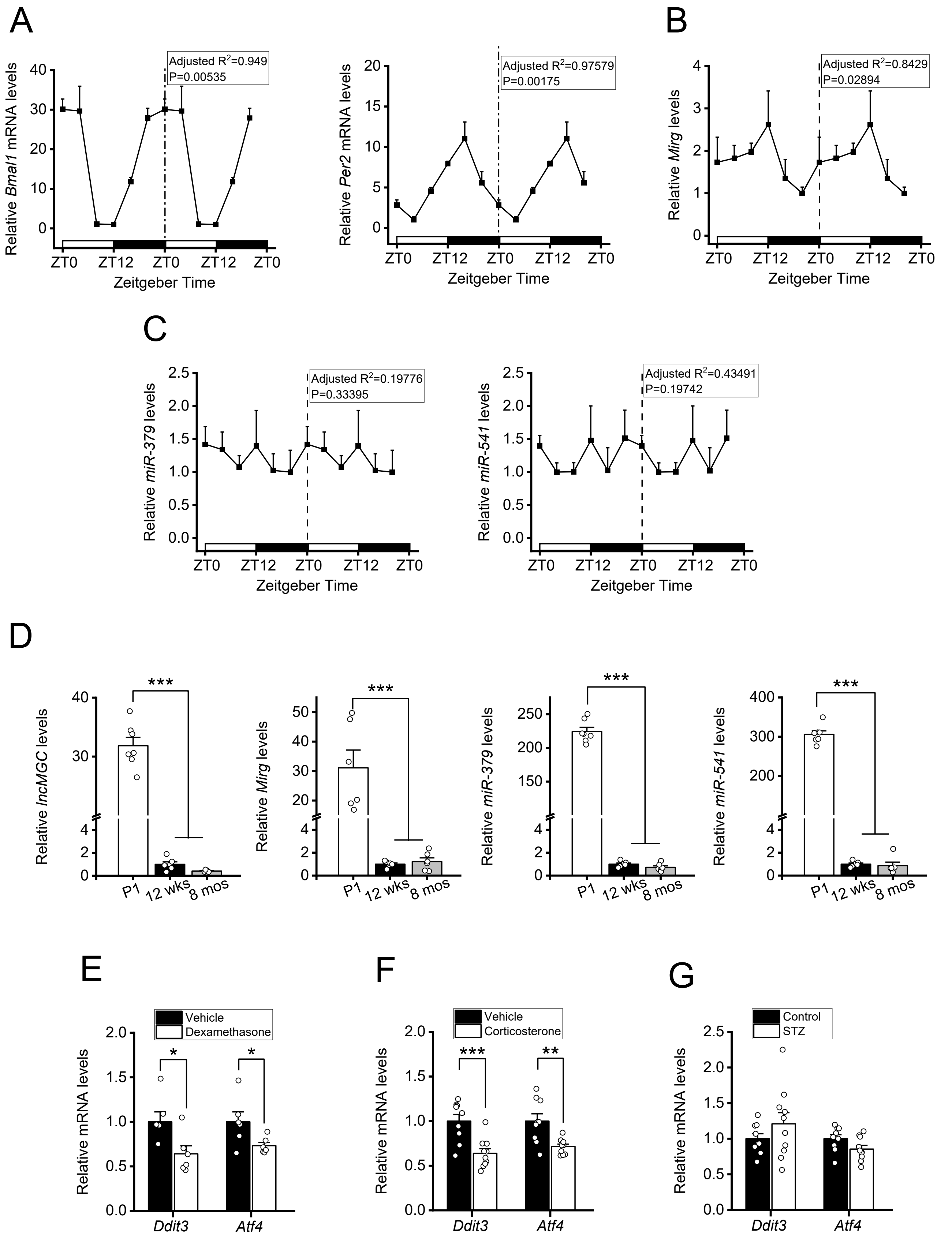

Supplemental Figure 4.

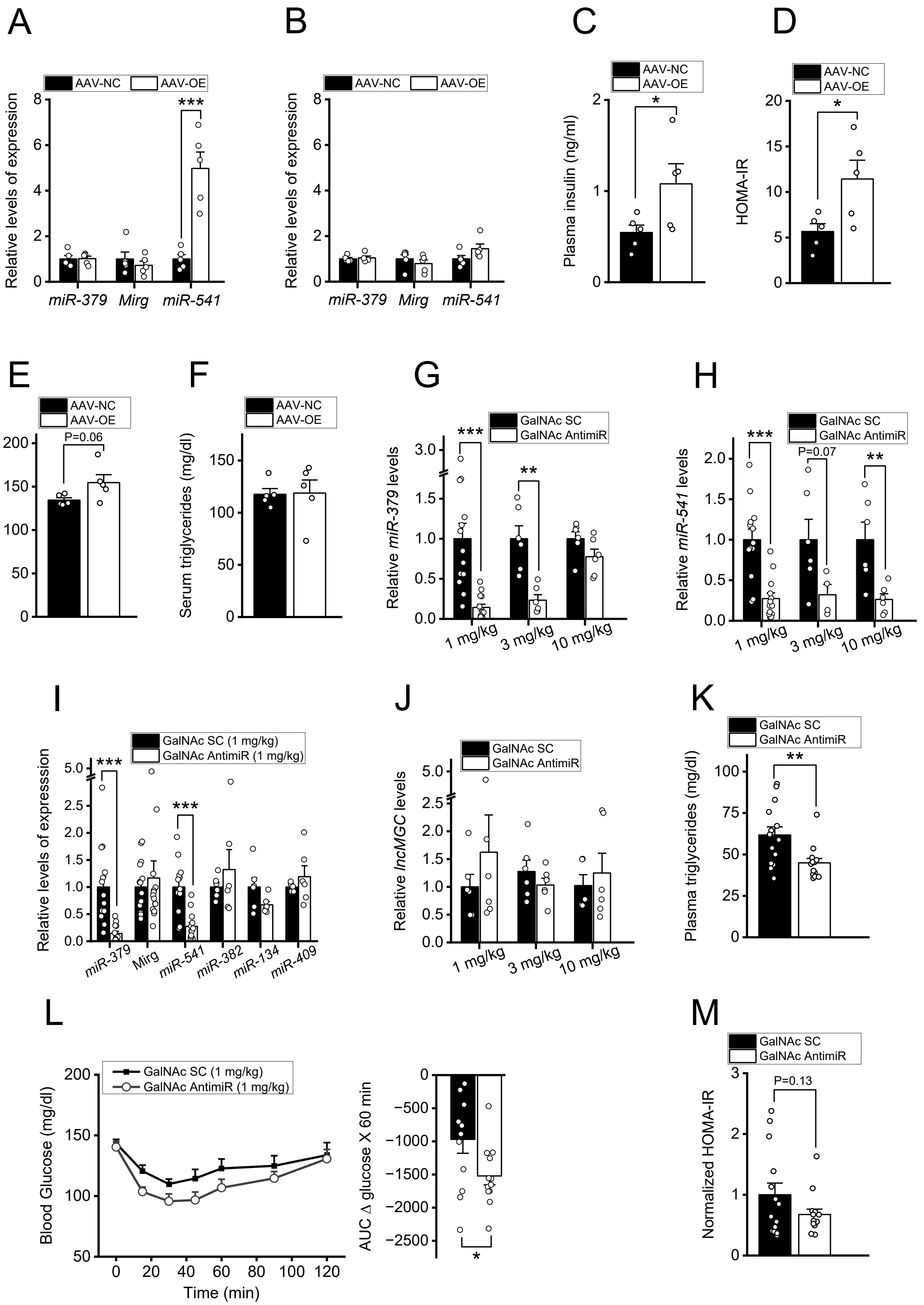

Supplemental Figure 5.

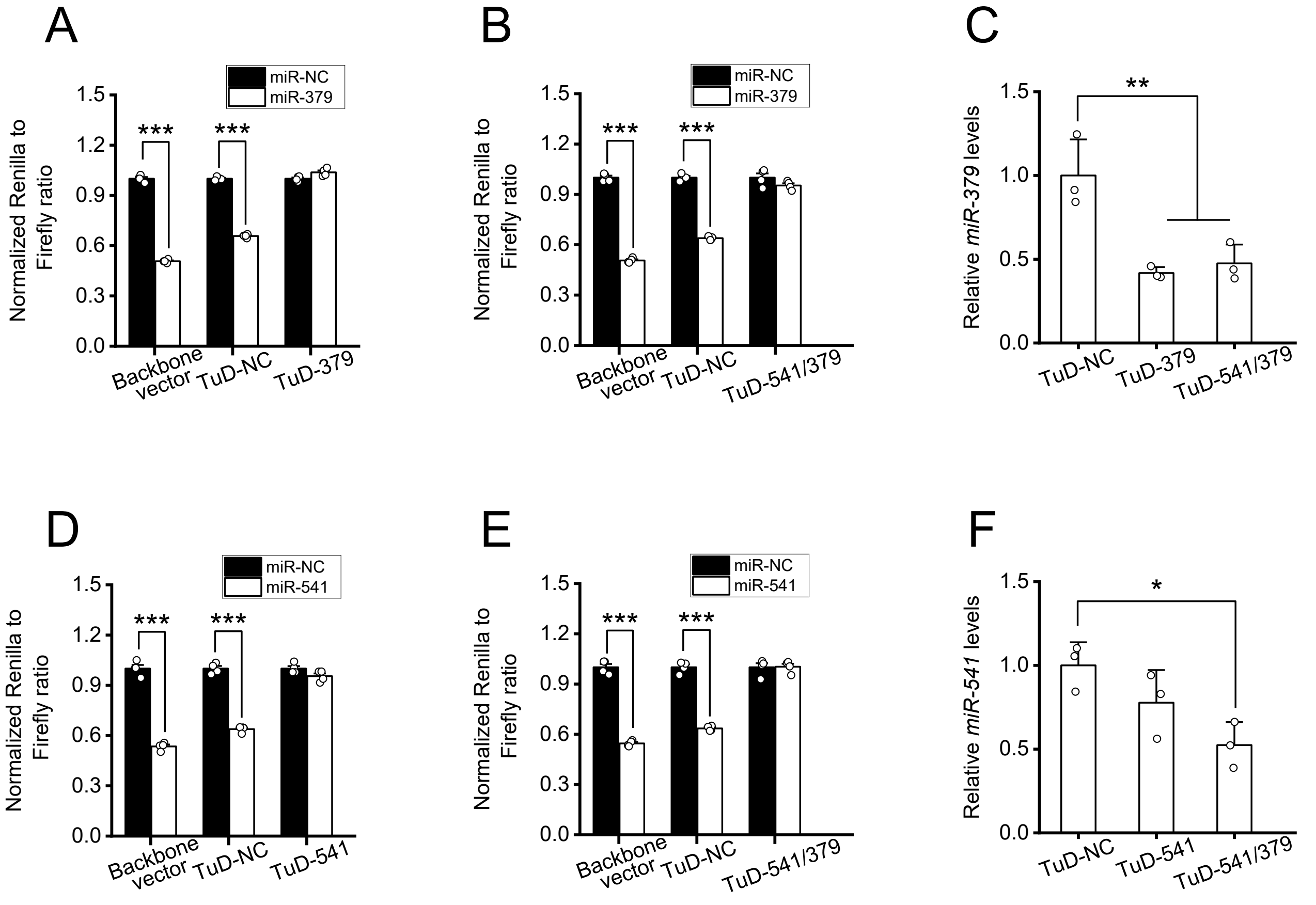

Supplemental Figure 6.

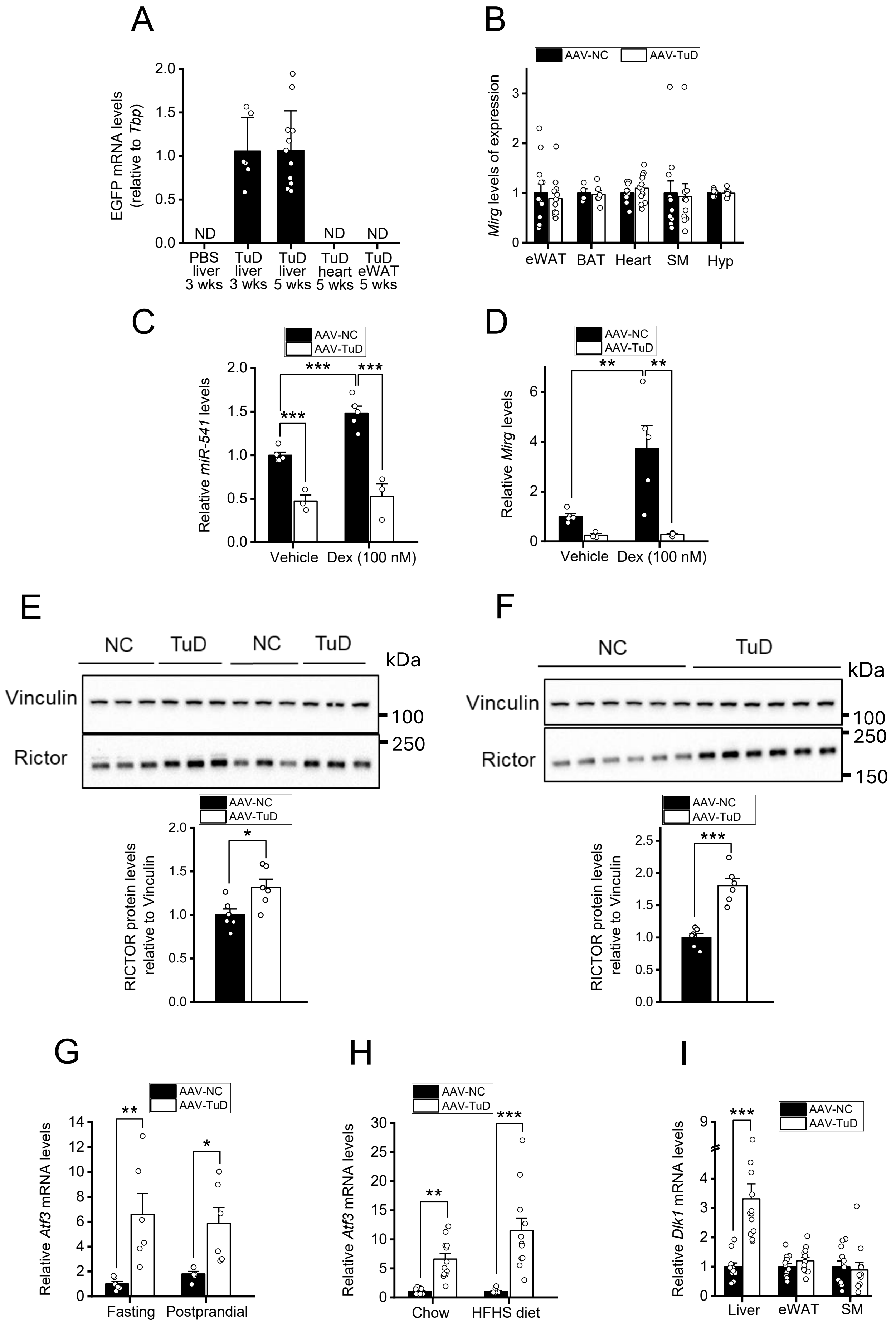

Supplemental Figure 7.

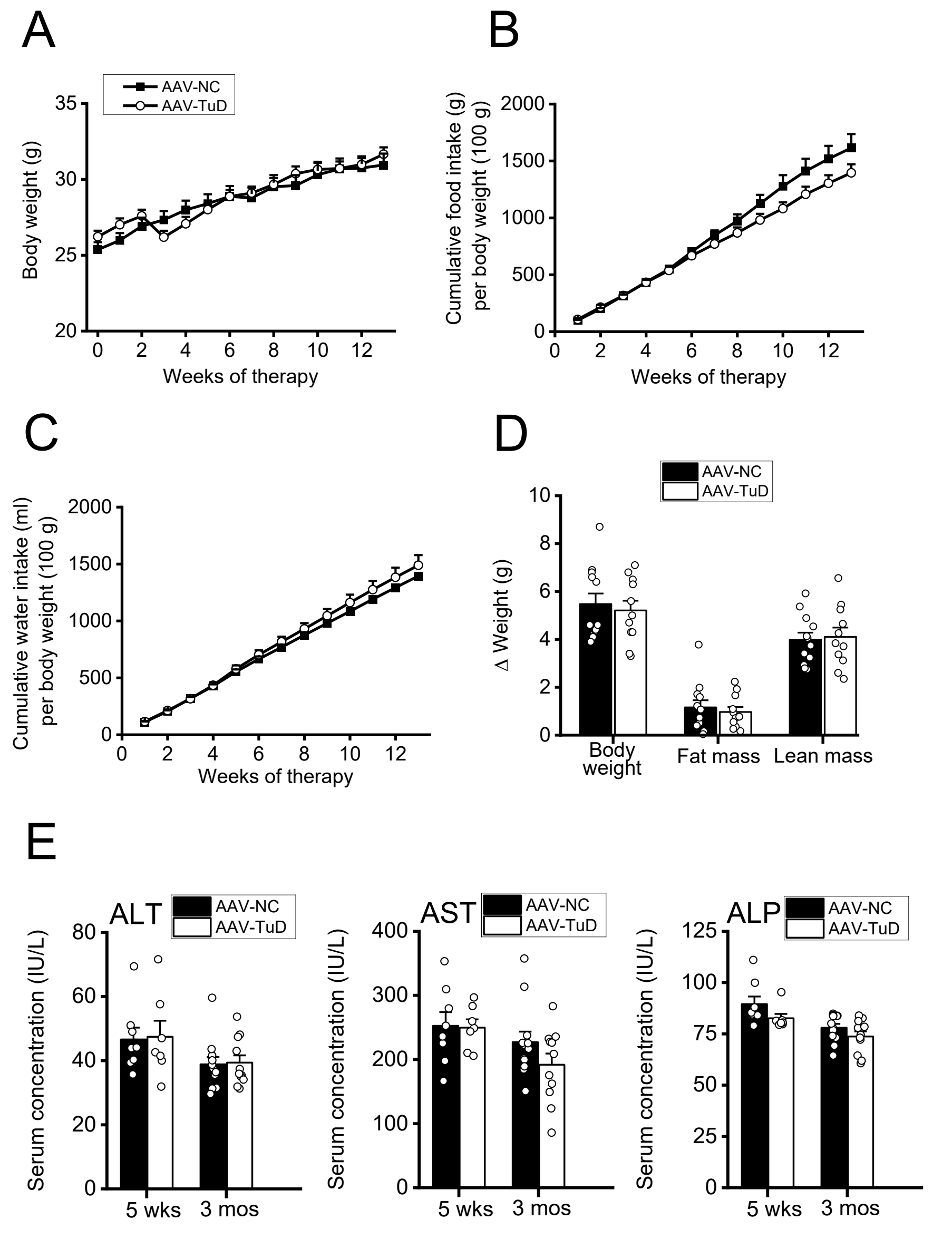

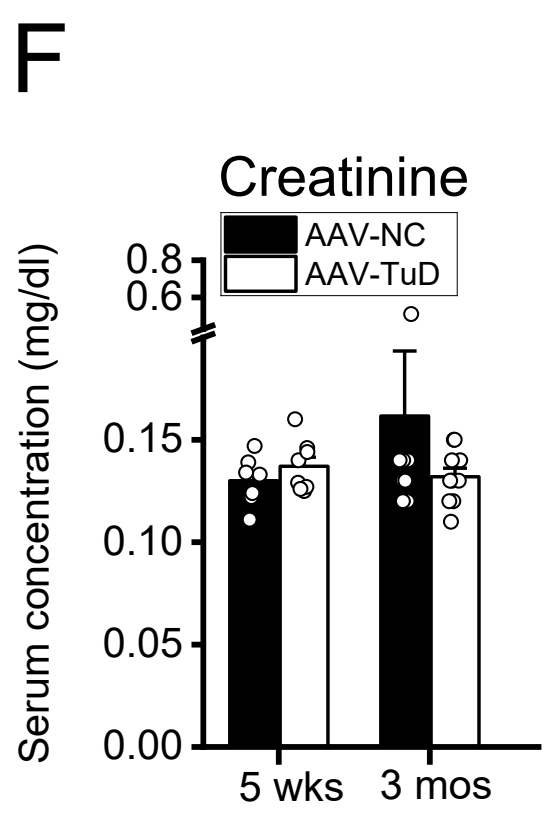

G

Group	AAV-NC	AAV-TUD
Total number of animals	12	11
Animals with 0 foci per 200X FOV (inflammation score = 0)	9	9
Animals with 1-2 foci per 200X FOV (inflammation score = 1)	3	2
Animals with 2-4 foci per 200X FOV (inflammation score = 2)	0	0
Animals with >4 foci per 200X FOV (inflammation score = 3)	0	0

Supplemental Figure 8.

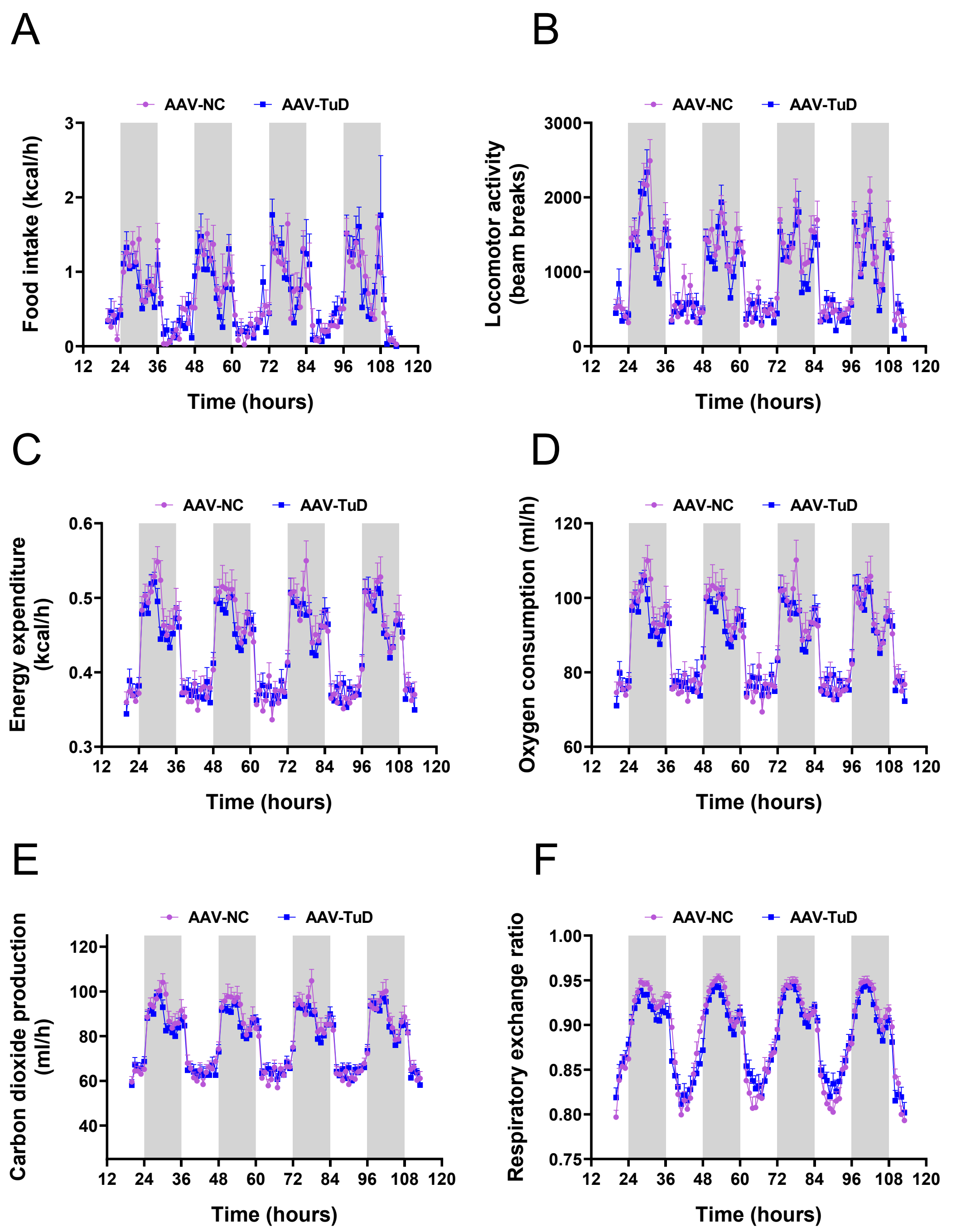

G

Effect	Mass	Group	Interaction
Food intake (Kcal/h)	0.0420*	0.7552	ns
Energy expenditure (Kcal/h)	0.2047	0.7839	ns
Oxygen consumption (ml/h)	0.2047	0.7685	ns
Carbon dioxide production (ml/h)	0.2078	0.8462	ns

H

Effect	Group
Locomotor activity	0.1948
Respiratory Exchange Ratio	0.6367

Supplemental Figure 9.

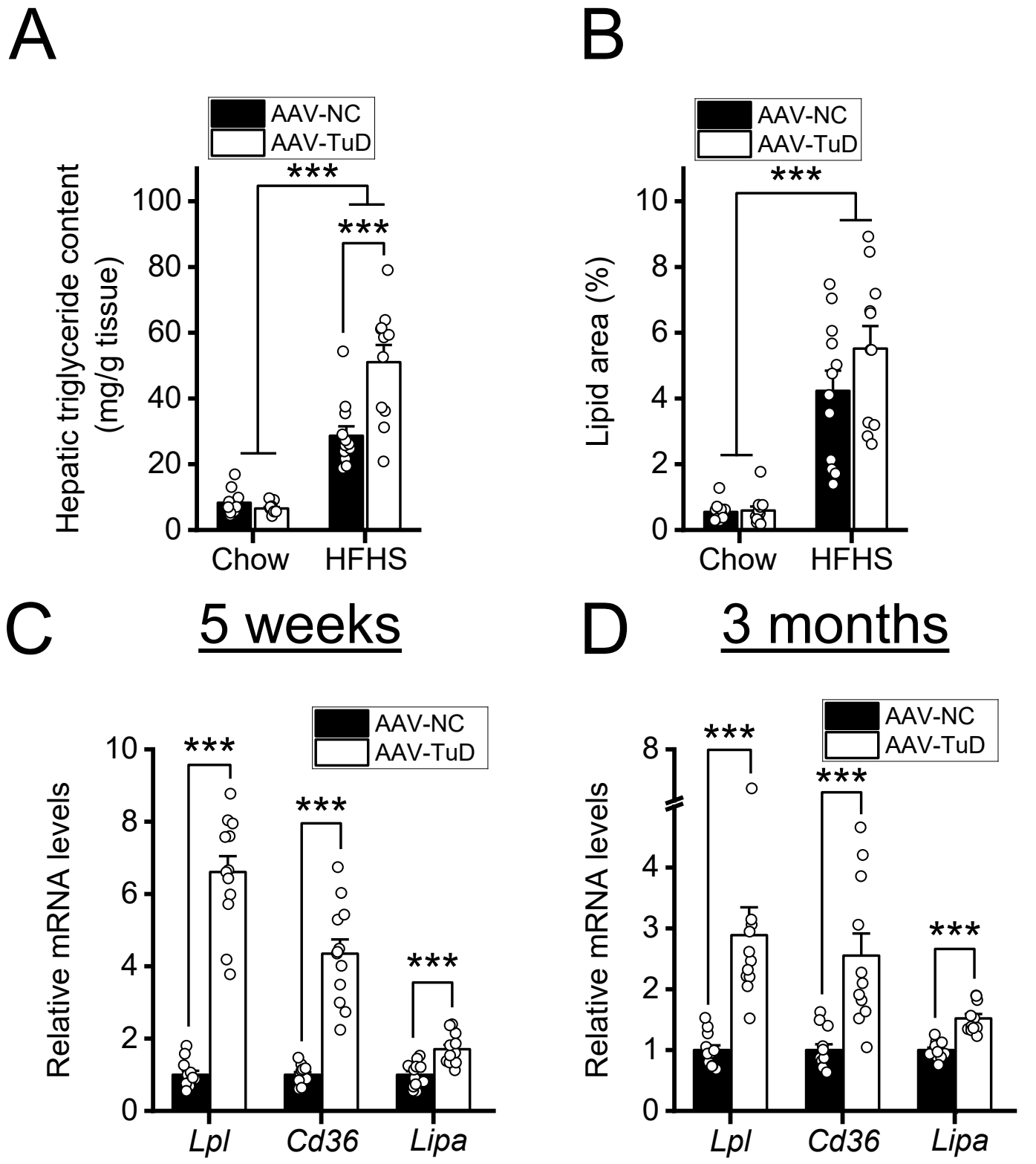

E

Gene (Symbol)	microRNAs binding 5'UTR	microRNAs binding CDS	microRNAs binding 3'UTR
Lpl	miR-667, miR-381, miR-3072	miR-380, miR-323, miR-329, miR-494, miR-679, miR-1193, miR-666, miR-667, miR-376b, miR-376c, miR-654, miR-381, miR-487b, miR-539, miR-544, miR-668, miR-485, miR-154, miR-541, miR-409, miR-412, miR-3072	miR-299a, miR-329, miR-494, miR-1193, miR-667, miR-376b, miR-300, miR-487b, miR-134,miR-485, miR-453, miR-496a, miR-412, miR-3072
Cd36	miR-299a, miR-299b, miR-323, miR-758, miR-329, miR-494, miR-1193, miR-666, miR-543, miR-667, miR-654, miR-381, miR-134, miR-668, miR-485, miR-154, miR-377, miR-541, miR-412, miR-3072	miR-379, miR-380, miR-1197, miR-323, miR-758, miR-329, miR-494, miR-679, miR-666, miR-543, miR-495, miR-667, miR-654, miR-300, miR-381, miR-487b, miR-539, miR-668, miR-485, miR-453, miR-154, miR-496a, miR-541, miR-412, miR-410, miR-3072	miR-379, miR-380, miR-1197, miR-758, miR-329, miR-679, miR-1193, miR-666, miR-495, miR-667, miR-381, miR-487b, miR-382, miR-134, miR-485, miR-453, miR-496a, miR-377, miR-409, miR-412, miR-3072
Lipa	miR-1197, miR-323, miR-667, miR-668, miR-453, miR-412	miR-299a, miR-299b, miR-323, miR-758, miR-494, miR-1193, miR-666, miR-667, miR-654, miR-487b, miR-134, miR-668, miR-485, miR-154, miR-496a, miR-3072	miR-379, miR-411, miR-299a, miR-299b, miR-1197, miR-758, miR-329, miR-666, miR-543, miR-667, miR-376a, miR-376b, miR-376c, miR-654, miR-300, miR-381, miR-539, miR-134, miR-485, miR-154, miR-496a, miR-377, miR-541, miR-412, miR-410, miR-3072

Supplemental Figure 10.

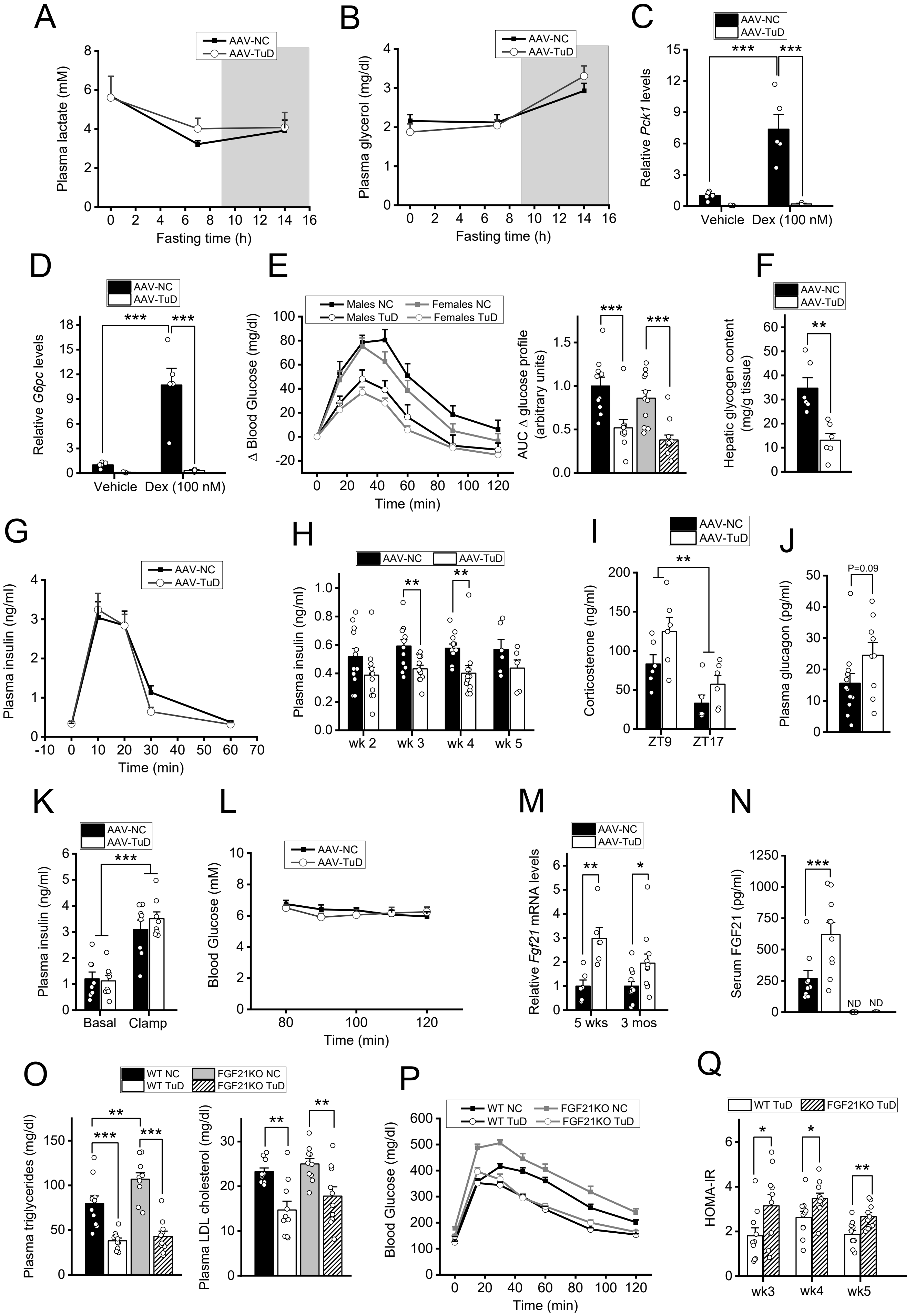

Supplementary Figure 11.

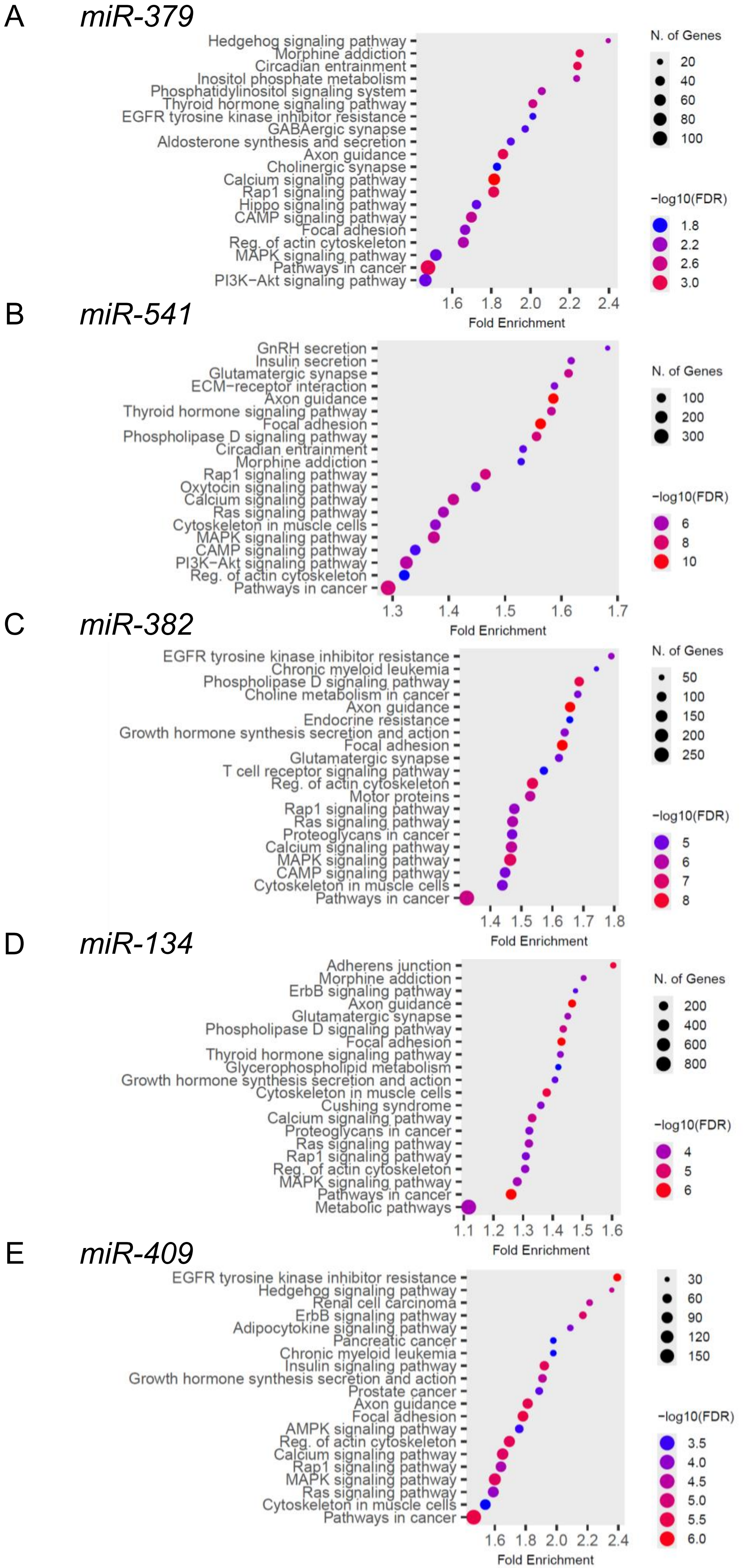

Supplementary Figure 12.

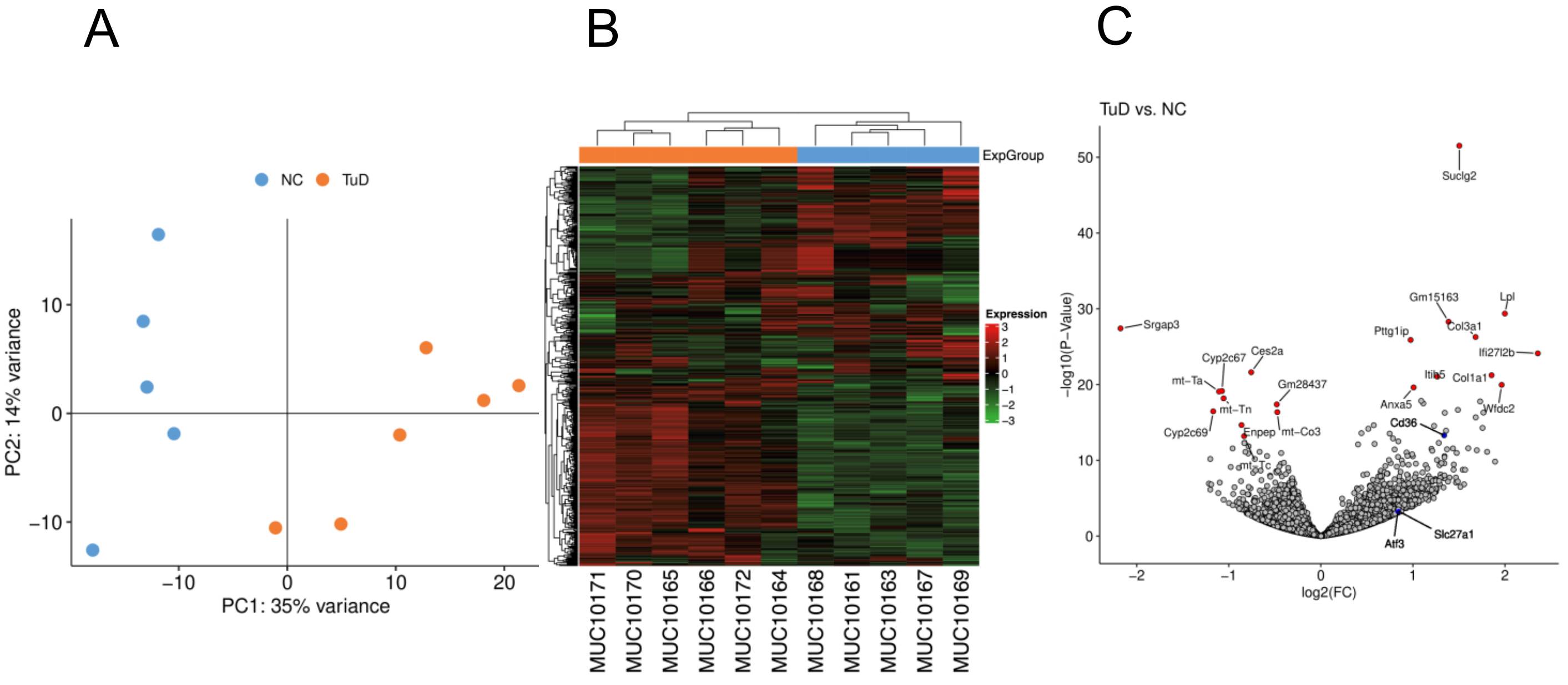

D

Pathway	Fold enrichment	FDR
ECM-receptor interaction	5.3	1.5E-09
Focal adhesion	3.1	2.1E-09
Cell adhesion molecules	3.5	1.4E-06
Regulation of actin cytoskeleton	2.5	6.8E-06
AGE-RAGE signaling	2.7	1.2E-03
PI3K-AKT signaling pathway	1.9	2.0E-03
Gap junction	2.8	3.4E-03
MicroRNAs in cancer	2.1	4.2E-03
Steroid biosynthesis	4.8	4.8E-03

E

Pathway	Fold enrichment	FDR
Ribosome	8.6	1.4E-36
Metabolic pathways	1.9	1.4E-10
Chemical carcinogenesis-reactive oxygen species	3.1	4.8E-07
Steroid hormone biosynthesis	5.5	5.8E-07
Oxidative phosphorylation	3.8	1.1E-06
Chemical carcinogenesis-DNA adducts	4.6	1.6E-05
Complement and coagulation cascades	4.1	6.2E-05
Retinol metabolism	3.6	8.5E-04

Supplementary Figure 13.

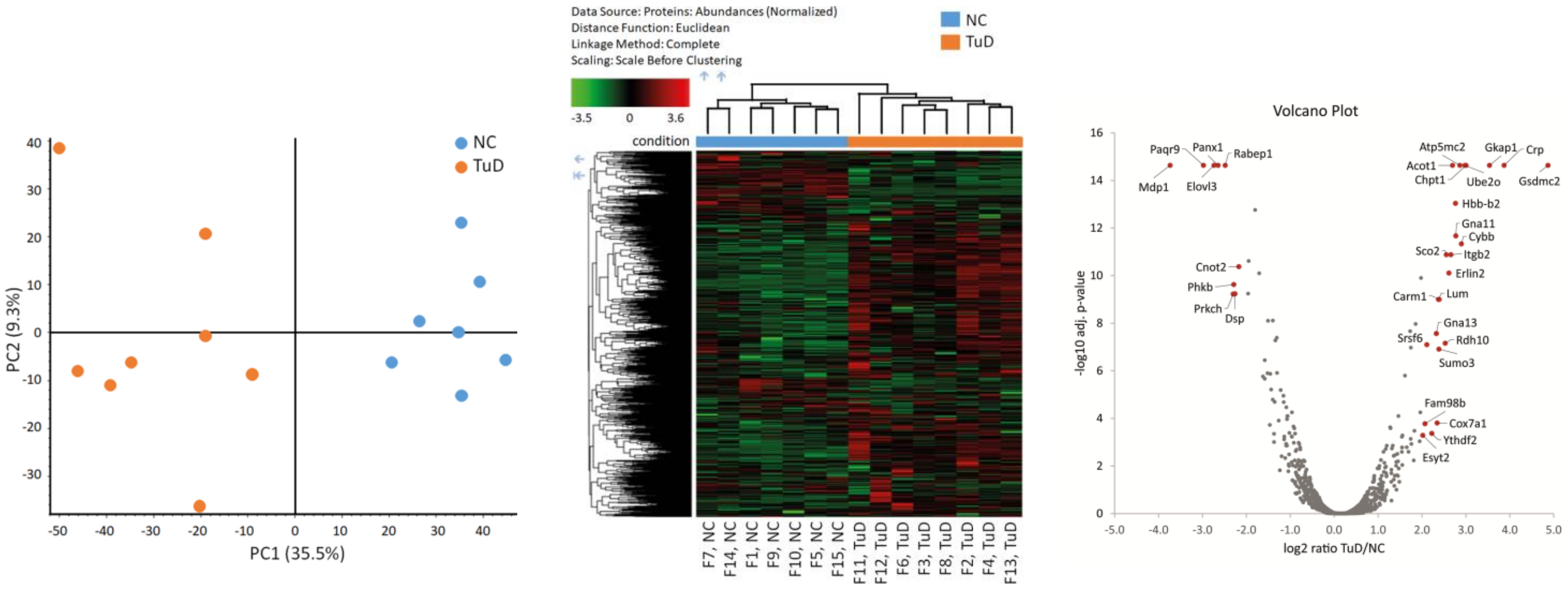

D

Pathway	ES	NES	FDR
Cholesterol biosynthesis	0.78	2.10	0.002
Activation of the mRNA	0.59	2.03	0.002
Translation initiation	0.54	2.01	0.002
Formation of a pool of free 40S subunits	0.54	2.00	0.003
Nonsense mediated decay	0.54	1.99	0.002

E

Pathway	ES	NES	FDR
Biological oxidations	-0.49	-2.39	0.000
Metabolism of amino acids	-0.44	-2.29	0.000
Bile acid and bile salt metabolism	-0.63	-2.29	0.000
Synthesis of bile acids	-0.66	-2.21	0.002
Fatty acyl-coA biosynthesis	-0.56	-1.92	0.028

F

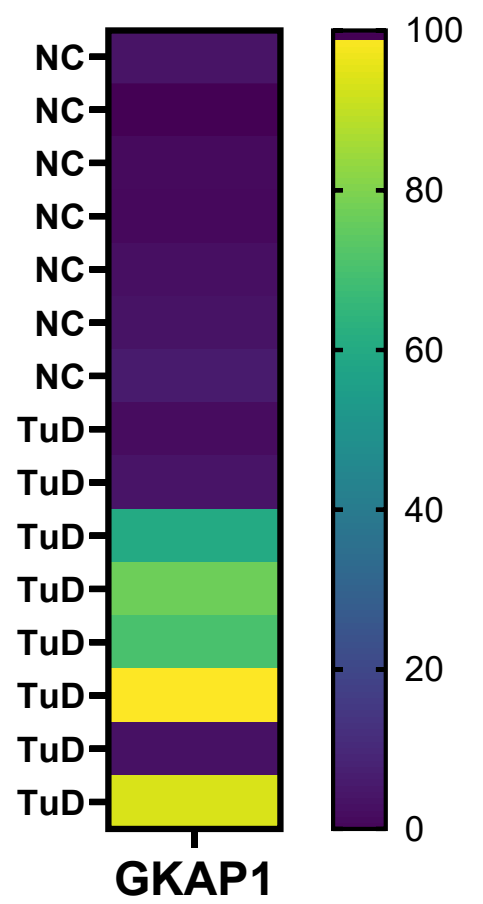

G

Gene (Symbol)	microRNAs binding 5‘UTR	microRNAs binding CDS	microRNAs binding 3‘UTR
Gkap1	*miR-679*	*miR-329, miR-679, miR-666, miR-543, miR-667, miR-376c, miR-300, miR-381,* ***miR-382***, ***miR-134***, *miR-668, miR-453, miR-496a,* ***miR-541***.	***miR-379***, *miR-411, miR-329, miR-666*

Supplemental Figure 14.

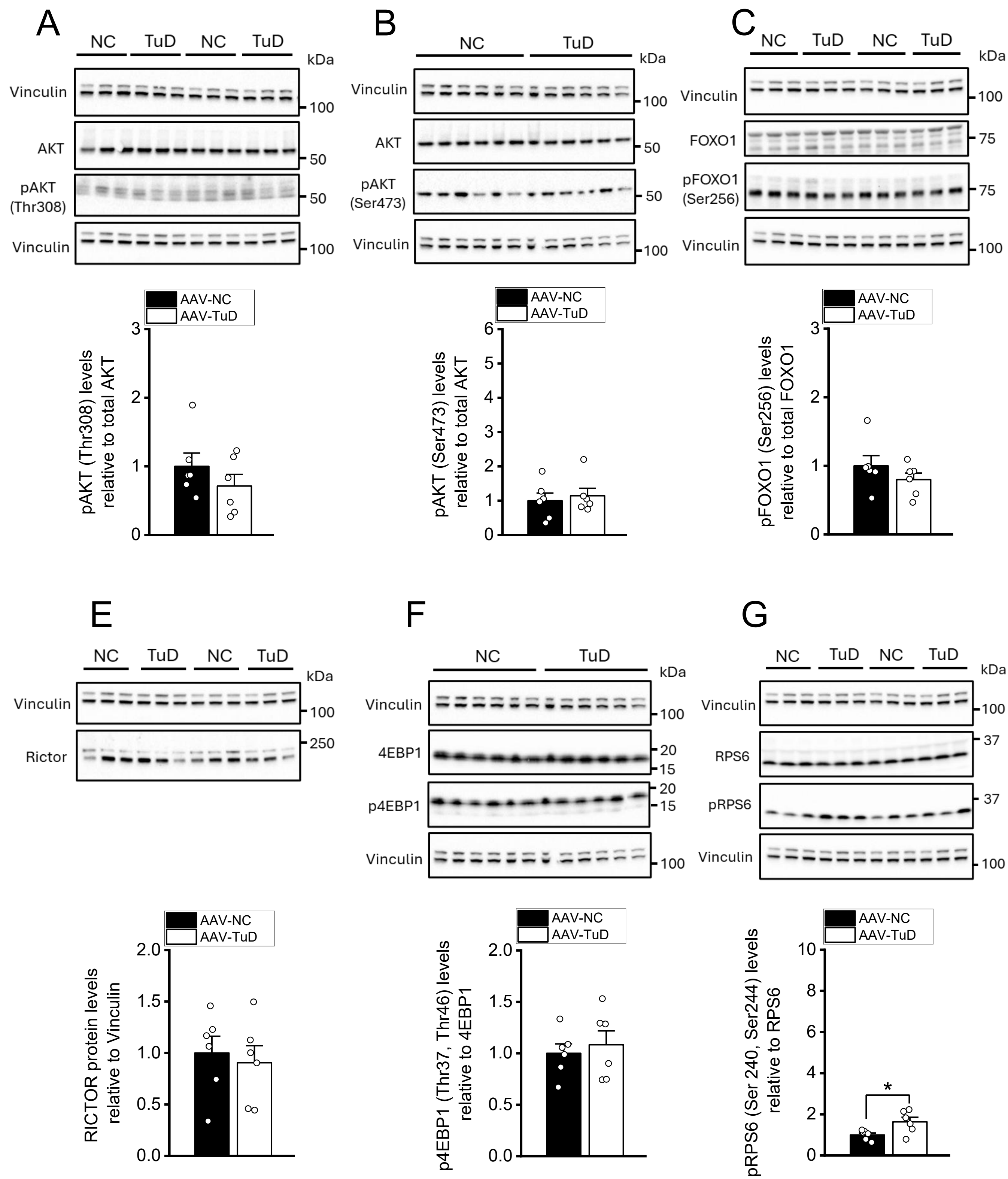

Supplemental Figure 15.

A

Metabolite	Fold Change	FDR
3-Aminoisobutyric acid	2.72	0.004
L-Hydroxyproline	2.88	0.01
B-Alanine	1.66	0.012
Glutaric acid	2.68	0.012
L-Threonine	2.02	0.013
L-Serine	3.35	0.015
2-Hydroxyglutaric acid	1.79	0.0019
3-Hydroxybutyric acid	1.61	0.03
L-Isoleucine	1.54	0.034
2-Aminobutanoic acid	1.82	0.04
L-Leucine	1.46	0.042
L-Valine	1.42	0.044
L-Proline	1.55	0.045
L-Methionine	1.97	0.047
2-Hydroxybutiric acid	1.87	0.05

B

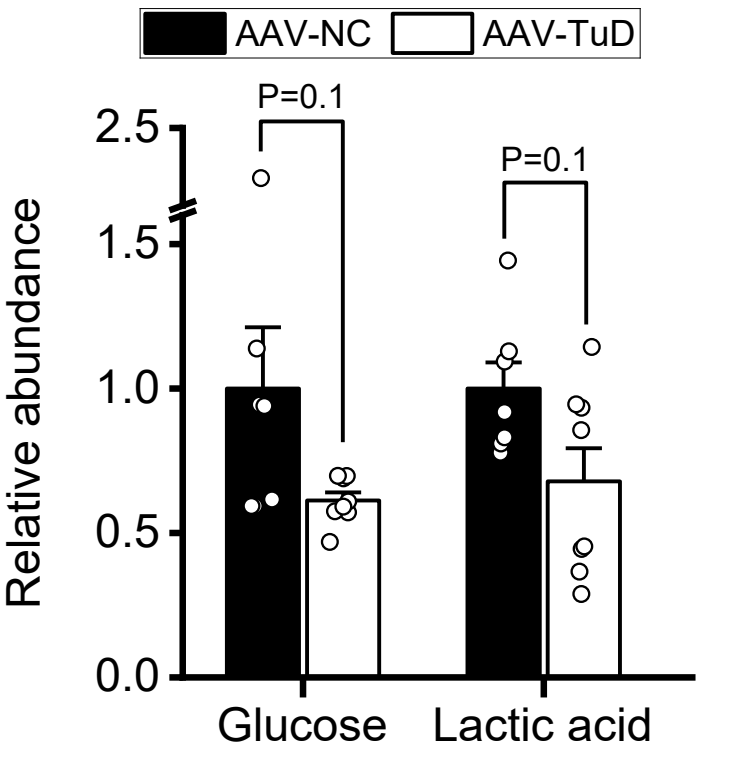

Supplemental Figure 16.

A

Protein	Abundance ratio
Chpt1	8.02
Sco2	5.87
Cox7a1	5.08
Kyat3	3.62
Aldh3a2	3.06
Mrpl15	2.91
Nif3l1	2.29
Suclg2	2.26
Apex1	1.88

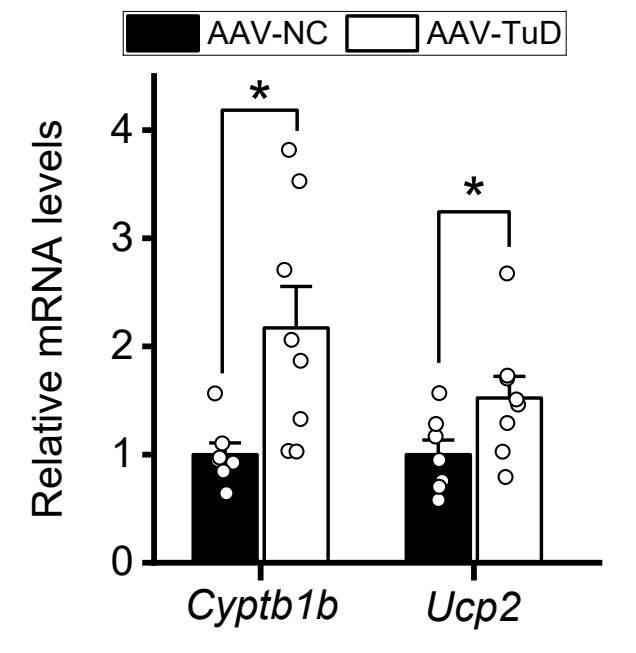

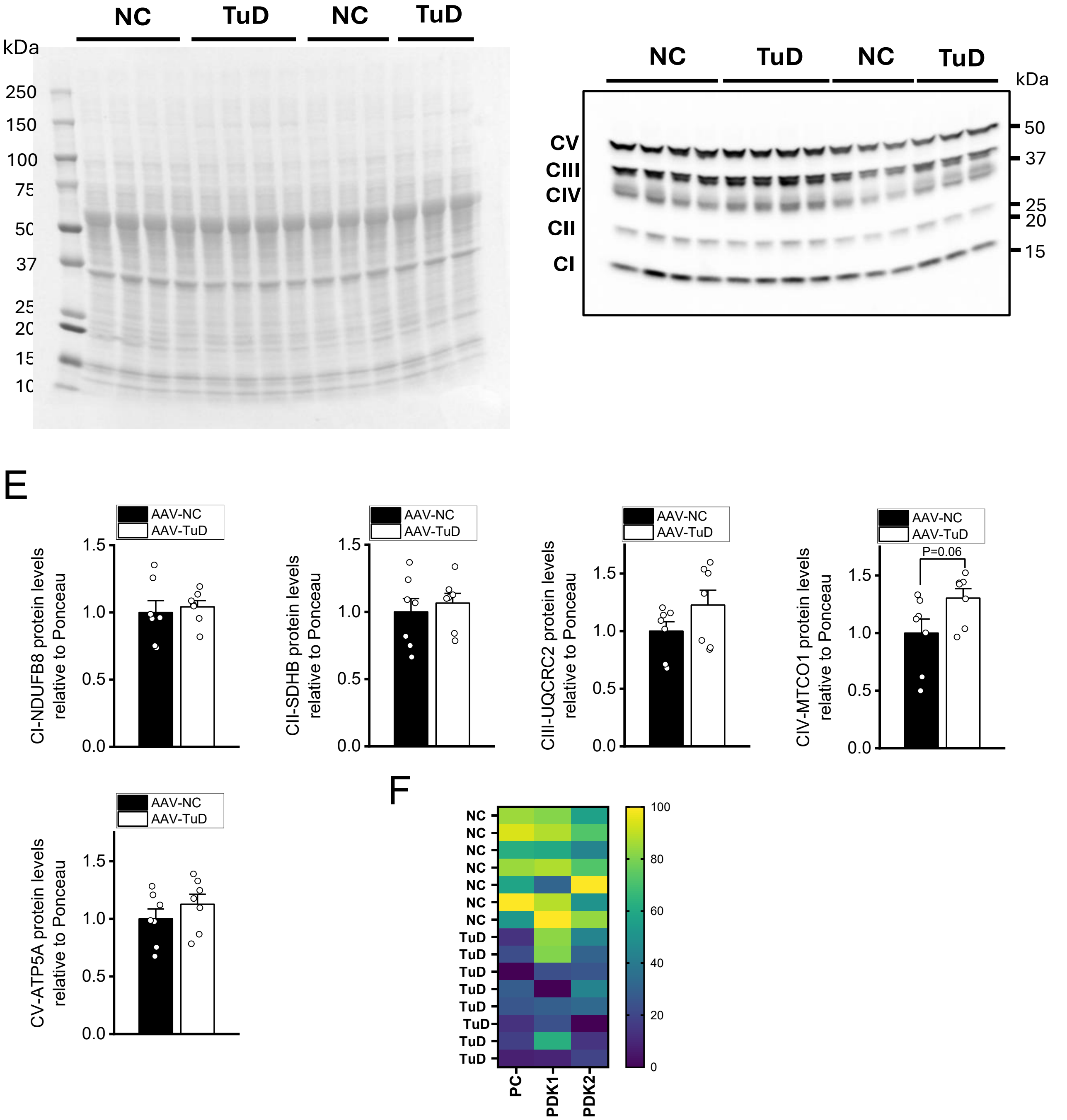

Supplemental Figure 17.

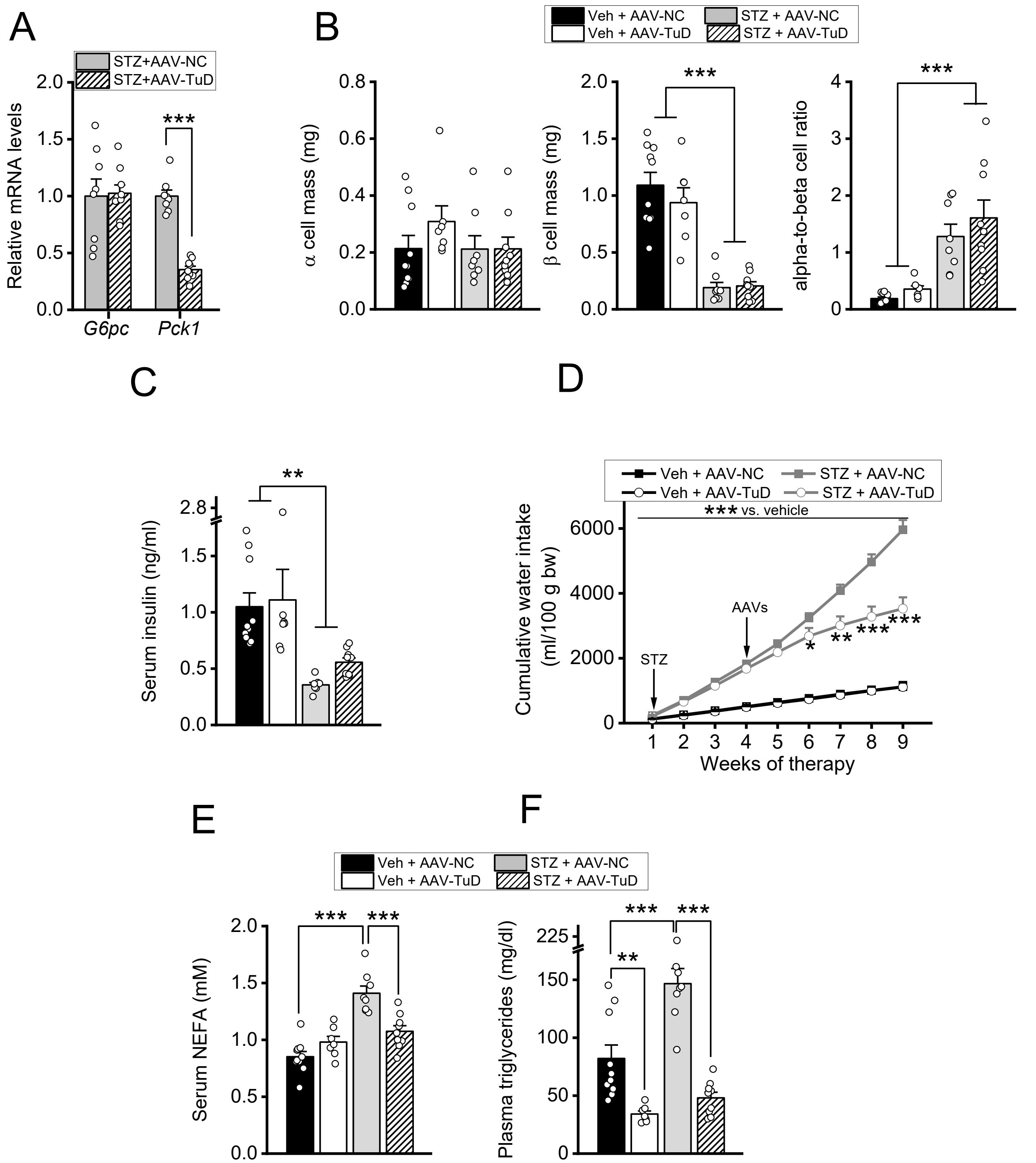

Supplemental Figure 18.

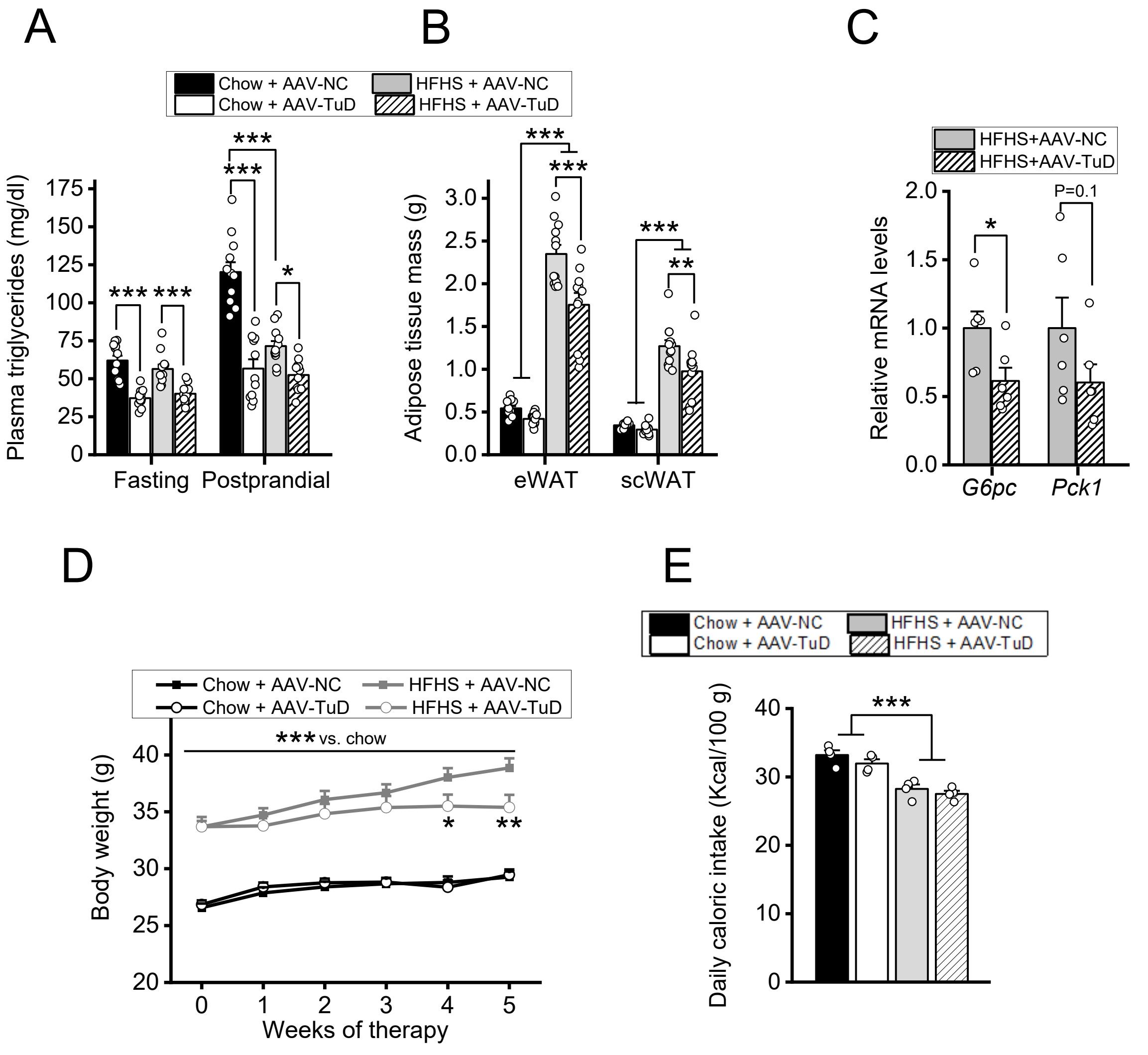

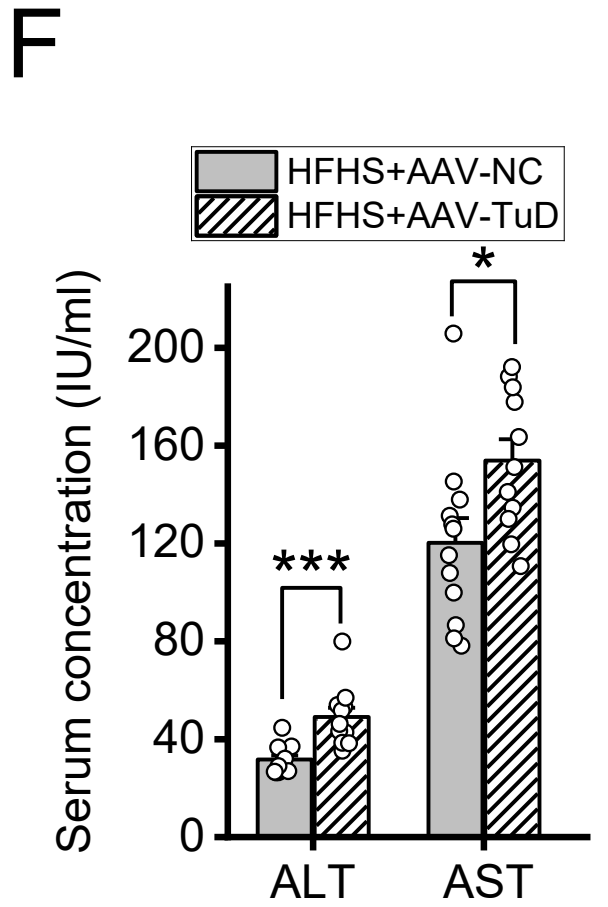

G

Group	AAV-NC	AAV-TUD
Total number of animals	12	11
Animals with 0 foci per 200X FOV (inflammation score = 0)	12	9
Animals with 1-2 foci per 200X FOV (inflammation score = 1)	0	2
Animals with 2-4 foci per 200X FOV (inflammation score = 2)	0	0
Animals with >4 foci per 200X FOV (inflammation score = 3)	0	0

SUPPLEMENTARY TABLE 1: Anthropometric characteristics and serum metabolic parameters of the participants in human study 1.

SUPPLEMENTARY TABLE 2: Oligonucleotide pairs used for the construction of TuD and miRNA expressing vectors.

SUPPLEMENTARY TABLE 3: Sequence and nucleotide modifications of the antagomirs used in the study.

SUPPLEMENTARY TABLE 4: Taqman expression assays used for qRT-PCR.

SUPPLEMENTARY TABLE 5: Primers used for qRT-PCR.

SUPPLEMENTARY TABLE 6: Antibodies used for immunoblots and immunostaining.

SUPPLEMENTARY TABLE 1

Parameters	Lean subjects	Subjects with obesity	Significance
Age (years old)	55±3.8	44.1±3.4	ns
Sex distribution (males/females)	3/2	0/11	NA
Body weight (kg)	69.0±5.1	132.2±3.6	P<0.001
BMI (kg/m^2)	23.7±0.5	47.6±1.6	P<0.001
Fasting plasma glucose (mM)	5.2±0.1	4.8±0.2	ns
Fasting plasma insulin (mU/L)	1.9±0.3	9.6±1.6	P=0.004
HOMA-IR	0.43±0.06	2.06±0.35	P=0.005
HbA1c (%)	5.23±0.06	5.27±0.09	ns
Serum triglycerides (mM)	0.89±0.06	1.68±0.13	P<0.001

SUPPLEMENTARY TABLE 2

Oligo strand	Sequence
OE_miR-NC top	5'TGCTGGAAATGTACTGCGCGTGGAGACGTTTTGGCCACTGACTGACGTCTCCACG CAGTACATTT
OE_miR-NC bottom	5'CCTGAAATGTACTGCGTGGAGACGTCAGTCAGTGGCCAAAACGTCTCCACGCGCA GTACATTTCC
OE_miR-541 top	5'TGCTGGCCAAAATCAGAGAAGGGATTCTGATGTTGGTCACACTCCAAGAGTTTTAA AATGAGTGGCGAACACAGAATCCATACTCTGCTTATGGC
OE_miR-541 bottom	5'CCTGGCCATAAGCAGAGTATGGATTCTGTGTTCGCCACTCATTTTAAAACTCTTGG AGTGTGACCAACATCAGAATCCCTTCTCTGATTTTGGCC
OE_miR-379 top	5'TGCTGAGAGATGGTAGACTATGGAACGTAGGCGTTATGTTTTTGACCTATGTAACA TGGTCCACTAACTCT
OE_miR-379 bottom	5'CCTGAGAGTTAGTGGACCATGTTACATAGGTCAAAAACATAACGCCTACGTTCCAT AGTCTACCATCTCTC
TuD-NC top	5'ATATATGTCGACGACGGCGCTAGGATCATCAACGCCGATATTTCCCTATTCCAACT CACCAAGTATTCTGGTCACAGAATACAACGCCGATATTTCCCTATTCCAACTCACCAA GATGATCCTAGCGCCGTCAGATCTATATAT
TuD-NC bottom	5'ATATATAGATCTGACGGCGCTAGGATCATCTTGGTGAGTTGGAATAGGGAAATATC GGCGTTGTATTCTGTGACCAGAATACTTGGTGAGTTGGAATAGGGAAATATCGGCGT TGATGATCCTAGCGCCGTCGTCGACATATAT
TuD antimiR-379 top	5'ATATATGTCGACGACGGCGCTAGGATCATCAACCCTACGTTCCAATCTTAGTCTAC CACAAGTATTCTGGTCACAGAATACAACCCTACGTTCCAATCTTAGTCTACCACAAGA TGATCCTAGCGCCGTCAGATCTATATAT
TuD antimiR-379 bottom	5'ATATATAGATCTGACGGCGCTAGGATCATCTTGTGGTAGACTAAGATTGGAACGTA GGGTTGTATTCTGTGACCAGAATACTTGTGGTAGACTAAGATTGGAACGTAGGGTTG ATGATCCTAGCGCCGTCGTCGACATATAT
TuD antimiR-541 top	5'ATATATGTCGACGACGGCGCTAGGATCATCAACAGTGTGACCAACATCATCTAGAA TCCCTTCAAGTATTCTGGTCACAGAATACAACAGTGTGACCAACATCATCTAGAATCC CTTCAAGATGATCCTAGCGCCGTCAGATCTATATAT
TuD antimiR-541 bottom	5'ATATATAGATCTGACGGCGCTAGGATCATCTTGAAGGGATTCTAGATGATGTTGGT CACACTGTTGTATTCTGTGACCAGAATACTTGAAGGGATTCTAGATGATGTTGGTCAC ACTGTTGATGATCCTAGCGCCGTCGTCGACATATAT
TuD antimiR-541/379 top	5'ATATATGTCGACGACGGCGCTAGGATCATCAACAGTGTGACCAACATCATCTAGAA TCCCTTCAAGTATTCTGGTCACAGAATACAACCCTACGTTCCAATCTTAGTCTACCAC AAGATGATCCTAGCGCCGTCAGATCTATATAT
TuD antimiR-541/379 bottom	5'ATATATAGATCTGACGGCGCTAGGATCATCTTGTGGTAGACTAAGATTGGAACGTA GGGTTGTATTCTGTGACCAGAATACTTGAAGGGATTCTAGATGATGTTGGTCACACT GTTGATGATCCTAGCGCCGTCGTCGACATATAT
AAV-NC top	5'ATATATGTCGACCAGTGGATCCTGGAGGCTTGCTGAAGGCTGTATGCTGAAATGTA CTGCGCGTGGAGACGTTTTGGCCACTGACTGACGTCTCCACGCAGTACATTTCAGG ACACAAGGCCTGTTACTAGCACTCACATGGAACAAATGGCCCAGATCTATATAT
AAV-NC bottom	5'ATATATAGATCTGGGCCATTTGTTCCATGTGAGTGCTAGTAACAGGCCTTGTGTCC TGAAATGTACTGCGTGGAGACGTCAGTCAGTGGCCAAAACGTCTCCACGCGCAGTA CATTTCAGCATACAGCCTTCAGCAAGCCTCCAGGATCCACTGGTCGACATATAT

SUPPLEMENTARY TABLE 3

**Antisense oligonucleotide**	**Sequence**
GalNAc-NC	5’(GalNAc)3(NHC6)csCbsasusAbsususGbscsgsCbsgsusAbsusasGbsuscsGbdCdAcsCbs asusAbsususGbscsgsCbsgsusAbsusasGbsuscsGb
GalNAc-AntimiR	5´(GalNAc)3(NHC6)usGbsascsCbsasasCbsasusCbsasgsAbsasusCbscscsTbdCdAcsCbs usasCbsgsusTbscscsAbsusasGbsu

Sequence code:

a, c, g, u: 2'-O-methyl residues.

Ab, Cb, Gb, Tb: LNA-residues.

s: phosphorothioate.

(NHC6): C6- amino linker.

(GalNAc)3: triantennary GalNAc cluster.

dCdA: DNA-linker, target site for cleavage

SUPPLEMENTARY TABLE 4

Assay name	miRBase ID	Assay ID
hsa-miR-337-5p	hsa-miR-337-5p	002156
hsa-miR-127	hsa-miR-127-3p	000452
mmu-miR-379	mmu-miR-379-5p	001138
hsa-miR-382	hsa-miR-382-5p	000572
mmu-miR-134	hsa-miR-134-5p	001186
*hsa-miR-541**	hsa-miR-541-5p	002200
mmu-miR-541	mmu-miR-541-5p	002562
hsa-miR-409-5p	hsa-miR-409-5p	002331
hsa-miR-29a	hsa-miR-29a-3p	002112
hsa-miR-122	hsa-miR-122-5p	002245
snoRNA202	664268	001232
RNU48	568908	001006
U6 snRNA	715680	001973
RNU58B	664242	001206
Tbp	NA	Mm01277042_m1
Mirg	NA	Mm01335848_m1
Lipa	NA	Mm00498820_m1
Fgf21	NA	Mm00840165_g1
Dlk1	NA	Mm00494477_m1

SUPPLEMENTARY TABLE 5

Gene ID	Forward primer	Reverse primer
Tbp	5‘CTACCGTGAATCTTGGCTGTAAAC	5‘AATCAACGCAGTTGTCCGTGGC
Lpl	5‘GCGTAGCAGGAAGTCTGACCAA	5‘AGCGTCATCAGGAGAAAGGCGA
Cd36	5‘AATTAGAACCGGGCCACGTA	5‘CGCCAACTCCCAGGTACAA
Atf3	5‘TTTGCTAACCTGACACCCTTTG	5‘AGAGGACATCCGATGGCAGA
lncMGC (5‘region)	5’ATTTTTCTGAGTTAGTGTGGCCTTCATCTG	5’TCAGGAACCATGGAACGGTGTTGACCCCTAG
lncMGC (central)	5’TTTGAGGAAGAGCCAGTGTC	5’GCCAAGCCAGAGAAATTTGC
lncMGC (3‘region)	5’CCTTCCTGGATCTCTCGCTT	5’GTGGGAGTTGAAACATGGGT
Igf1	5‘CTCTTCTGCTTGCTAAATCTCAC	5‘GCCAGGTAGAAGAGGTGTGAAG
Cpypt1b	5‘TCTTCTTCCGACAAACCCTGA	5‘GAGACGGACACAGATAGCCC
Ucp2	5‘ATGGTTGGTTTCAAGGCCACA	5‘CGGTATCCAGAGGGAAAGTGAT
*EGFP**	5‘GAGCGCACCATCTTCTTCAAG	5‘TGTCGCCCTCGAACTTCAC

* For the detection of EGFP, the aforementioned primers were combined with the following probe: 5’ACGACGGCAACTACA

SUPPLEMENTARY TABLE 6

Antigen	Company	Catalog number
AKT	Cell Signaling	9272
Phospho-AKT(Thr308)	Cell Signaling	4056
Phospho-AKT(Ser473)	Cell Signaling	9271
FOXO1	Santa Cruz Biotechnology	sc-11350
Phospho-FOXO1(Ser256)	Cell Signaling	9461
mTOR	Cell Signaling	2983
Phospho-mTOR(Ser2448)	Cell Signaling	5536
4E-BP1	Cell Signaling	9452
Phospho-4E-BP1(Thr37/46)	Cell Signaling	2855
S6 Ribosomal Protein	Cell Signaling	2217
Phospho-S6RP(Ser240/244)	Cell Signaling	5364
Vinculin	Abcam	ab-129002
Rictor	Cell Signaling	21145
OxPhos (antibody cocktail)	Thermo Fischer Scientific	45-8099
PLIN-2	Progen	GP40
Goat anti-Guinea Pig igG-Alexa Fluor 555 conjugate	Thermo Fischer Scientific	A-21435
TOMM20	Abcam	ab-186735
Prohibitin	Abcam	ab-28172
Goat anti-Rabbit IgG-HRP conjugate	Bio-Rad	1721019
Goat anti-Rabbit IgG-Alexa Fluor 555 conjugate	Thermo Fischer Scientific	A-21428
