# Supplementary material for: Combinatorial targeting of the miR-379/miR-410 cluster normalizes glucose and lipid homeostasis in male models of diabetes and obesity
Source: Nat Commun. 2026 Sep 21;17:10014. doi: 10.1038/s41467-026-77808-2 (PMC13594115; doi:10.1038/s41467-026-77808-2)
Supplement: Supplementary file 2 [file 41467_2026_77808_MOESM2_ESM.pdf]

nature portfolio

Corresponding author(s): Stephan Herzig

Last updated by author(s): Jul 30, 2026

Reporting Summary

Nature Portfolio wishes to improve the reproducibility of the work that we publish. This form provides structure for consistency and transparency in reporting. For further information on Nature Portfolio policies, see our Editorial Policies and the Editorial Policy Checklist.

Statistics

For all statistical analyses, confirm that the following items are present in the figure legend, table legend, main text, or Methods section.

n/a	Confirmed	
☐	☒	The exact sample size (*n*) for each experimental group/condition, given as a discrete number and unit of measurement
☐	☒	A statement on whether measurements were taken from distinct samples or whether the same sample was measured repeatedly
☐	☒	The statistical test(s) used AND whether they are one- or two-sided<br>*Only common tests should be described solely by name; describe more complex techniques in the Methods section.*
☐	☒	A description of all covariates tested
☐	☒	A description of any assumptions or corrections, such as tests of normality and adjustment for multiple comparisons
☐	☒	A full description of the statistical parameters including central tendency (e.g. means) or other basic estimates (e.g. regression coefficient) AND variation (e.g. standard deviation) or associated estimates of uncertainty (e.g. confidence intervals)
☐	☒	For null hypothesis testing, the test statistic (e.g. *F*, *t*, *r*) with confidence intervals, effect sizes, degrees of freedom and *P* value noted<br>*Give P values as exact values whenever suitable.*
☒	☐	For Bayesian analysis, information on the choice of priors and Markov chain Monte Carlo settings
☒	☐	For hierarchical and complex designs, identification of the appropriate level for tests and full reporting of outcomes
☐	☒	Estimates of effect sizes (e.g. Cohen's *d*, Pearson's *r*), indicating how they were calculated

Our web collection on statistics for biologists contains articles on many of the points above.

Software and code

Policy information about availability of computer code

Data collection	Collection of semiquantitative real-time PCR data was conducted in an Applied Biosystems® QuantStudio™ 7 Flex Real-Time PCR System, with QuantStudio software. Imaging of immunoblots was performed on a ChemiDoc imaging system using Image Lab software.
Data analysis	Calculations of distance correlations were performed using the energy package in R, version 4.4.2. Nonlinear curve fitting calculation was performed using OriginPro 2024 software. ANCOVA/generalized linear model using lean body mass as covariate was calculated by CalR2 to compare data on food intake, energy expenditure, oxygen consumption and carbon dioxide production collected by the TSE's PhenoMaster system. Comparison of different experimental groups either by Student's t test, one-way or two-way ANOVA, as appropriate was performed with GraphPad Prism 9. Proteomics raw data was analyzed using the Proteome Discoverer software 2.4. Gene set enrichment analysis was conducted using the GSEA software developed by the UC San Diego and the Broad Institute. The ChromaTof v5.50 software was used for metabolimics data processing. The resulting signal intensities (peak area) were transformed and analyzed using Perseus software. The reads of the transcriptomic analysis were trimmed by BBDuk and aligned to mouse genome GRCm38 using STAR 2.5.3a. Gene read quantification was performed using Rsubread. Differential expression analysis was performed with DESeq2 (1.38.3).Downstream enriched pathway analyses were run utilizing ShinyGO 0.85.1 software. Potential targets of miRNAs were predicted using miRWalk. lConfocal microscopy image analyses were performed using Fiji (ImageJ, v.2.0.0-rc-69/1.51w).

For manuscripts utilizing custom algorithms or software that are central to the research but not yet described in published literature, software must be made available to editors and reviewers. We strongly encourage code deposition in a community repository (e.g. GitHub). See the Nature Portfolio guidelines for submitting code & software for further information.

Data

Policy information about availability of data

All manuscripts must include a data availability statement. This statement should provide the following information, where applicable:

- Accession codes, unique identifiers, or web links for publicly available datasets
- A description of any restrictions on data availability
- For clinical datasets or third party data, please ensure that the statement adheres to our policy

All data generated or analyzed during this study are included in this manuscript (and its supplementary information and Source Data files). The mass spectrometry proteomics data have been deposited to the ProteomeXchange Consortium via the PRIDE partner repository with the dataset identifier PXD044478 (www.ebi.ac.uk/pride/archive/projects/PXD044478). The complete transcriptomic datasets were deposited to the National Center for Biotechnology Information's Gene Expression Omnibus Database and are accessible through GEO Series accession number GSE304566 (www.ncbi.nlm.nih.gov/geo/query/acc.cgi?acc=GSE304566). Metabolomics data are available at Mendeley data (doi: 10.17632/76zzw9gd4b.1). Source data are provided as Source Data files.

Research involving human participants, their data, or biological material

Policy information about studies with human participants or human data. See also policy information about sex, gender (identity/presentation), and sexual orientation and race, ethnicity and racism.

Reporting on sex and gender	Sex of the study participants was self reported. The manuscript includes two different studies involving human biological material. In a first study including a small number of participants, 5 lean and 11 with obesity, sex was not considered as an experimental variable given the small number of male participants (only 3). However, liver samples from a larger cohort (human study 2 including 24 lean subjects, 14 male and 10 female; and 42 subjects with obesity, 13 male and 29 female) were then studied to specifically evaluate the effects of sex in the hepatic expression of the miRNAs of interest.
Reporting on race, ethnicity, or other socially relevant groupings	Ethnicity of the subjects was not considered as an experimental variable.
Population characteristics	The anthropometric characteristics and metabolic profile of the subjects belonging to human study 1are shown in Supplementary table 1 and corresponding source data file. For human study 2, the information on sex, age and BMI is described in the materials and methods section and it is also contained in the source data file for supplemental figure 1.
Recruitment	For human study 1: Liver samples from a total of 22 individuals (5 self-reported as male and 17 self-reported as female) were obtained from the Leipzig Obesity Biobank. Five of the subjects had a BMI below 25 kg/m2 and were considered as controls, while the other 17 presented BMI ranging between 39-55 kg/m2. Out of the obese participants, 6 presented overt type 2 diabetes and were taking antidiabetic medications, these subjects were excluded from the analysis due to the confounder effects that the drugs would have on the metabolic parameters we intended to correlate with the expression of the miRNAs (e.g. triglycerides and HOMA-IR). The liver biopsies were taken when the subjects presented in the clinic to undergo elective cholecystectomy, explorative laparotomy, or gastric sleeve resection.<br>For human study 2 (evaluation of sex-dependent effects in the hepatic expression of the miRNAs), liver samples from non-diabetic lean subjects were provided by the Tissue Bank of the National Center for Tumor Diseases (NCT) in Heidelberg; while liver samples from non-diabetic obese subjects were obtained from the Leipzig Obesity Biobank.
Ethics oversight	Samples obtained from NCT were provided in accordance with the with the regulations of the tissue bank and the approval of the ethics committee of Heidelberg University. All protocols from study 1 and samples of study 2 obtained from the Leipzig Obesity Biobank were approved by the Ethics Committee of the University of Leipzig and performed in accordance to the declaration of Helsinki.

Note that full information on the approval of the study protocol must also be provided in the manuscript.

Field-specific reporting

Please select the one below that is the best fit for your research. If you are not sure, read the appropriate sections before making your selection.

☒ Life sciences ☐ Behavioural & social sciences ☐ Ecological, evolutionary & environmental sciences

For a reference copy of the document with all sections, see nature.com/documents/nr-reporting-summary-flat.pdf

Life sciences study design

All studies must disclose on these points even when the disclosure is negative.

Sample size	Considering the average levels and variation of certain parameters of interest (including glucose, triglyceride and cholesterol levels, as well as HOMA-IR values) from previous studies in the lab, on-line sample size calculators were applied to calculate the minimum sample size required to obtain a 15% difference in the parameters of interest between two independent groups. A significance of 0.05 and a power of 0.8 were

	considered by default. Once the first studies yielded significant results, the sample size of following studies was determined based on the new knowledge. The power of the sample size chosen for our studies is supported by the finding of consistent significant effects.
Data exclusions	Data from 6 subjects were excluded from human study 1, as they presented overt type 2 diabetes and were taking antidiabetic medications that would impact metabolic parameters we intended to correlate with the expression of the miRNAs (e.g. triglycerides and HOMA-IR). No mice were excluded from the animal studies.
Replication	Luciferase experiments in HEK293A cells were repeated a total of 4 times, similar effects were observed every time, as depicted in the corresponding supplemental figure representing the average measurements of each independent experiment. Hepatic-specific repression of the miR-379/miR-410 cluster was induced in several studies. Robust metabolic effects in glycemia, circulating lipids, glucose clearance and insulin sensitivity were consistently observed in all of them. Importantly, this manipulation was tested across different metabolic conditions, including male and female animals on regular chow, as well as diet-induced obese animals (prediabetic) and mice treated with STZ (diabetic). Overall, demonstrating the consistency of our findings.
Randomization	Human subjects were allocated in two groups based on their BMI. Those individuals with a BMI below 27 kg/m2 were considered controls,while the obese group included individuals with a BMI ranging between 30-55 kg/m2. For animal studies, body weight and body composition of the animals were measured prior to the study to ensure that mice with comparable body weight and composition were assigned to the different groups, to avoid a potential impact of those parameters on the metabolic characterization of the animals.
Blinding	Researchers were generally blinded to group allocation in animal studies except for the first author, who was responsible to assign the animals to their respective treatment group. In human studies, researchers performing the determinations were blinded to group allocation.

Reporting for specific materials, systems and methods

We require information from authors about some types of materials, experimental systems and methods used in many studies. Here, indicate whether each material, system or method listed is relevant to your study. If you are not sure if a list item applies to your research, read the appropriate section before selecting a response.

Materials & experimental systems

n/a	Involved in the study
☐	☒ Antibodies
☐	☒ Eukaryotic cell lines
☒	☐ Palaeontology and archaeology
☐	☒ Animals and other organisms
☒	☐ Clinical data
☒	☐ Dual use research of concern
☒	☐ Plants

Methods

n/a	Involved in the study
☒	☐ ChIP-seq
☒	☐ Flow cytometry
☒	☐ MRI-based neuroimaging

Antibodies

Antibodies used	AKT, Cell Signaling, catalog# 9272 (1:1000 dilution).<br>Phospho-AKT(Thr308), Cell Signaling, catalog# 4056 (1:1000 dilution).<br>Phospho-AKT(Ser473), Cell Signaling, catalog# 9271 (1:1000 dilution).<br>FOXO1, Santa Cruz Biotechnology, catalog# sc-11350 (1:1000 dilution).<br>Phospho-FOXO1(Ser256), Cell Signaling , catalog# 9461 (1:1000 dilution).<br>mTOR, Cell Signaling, catalog# 2983 (1:1000 dilution).<br>Phospho-mTOR(Ser2448), Cell Signaling, catalog# 5536 (1:1000 dilution).<br>4E-BP1, Cell Signaling, catalog# 9452 (1:1000 dilution).<br>Phospho-4E-BP1(Thr37/46), Cell Signaling , catalog# 2855 (1:1000 dilution).<br>S6 Ribosomal Protein, Cell Signaling, catalog# 2217 (1:1000 dilution).<br>Phospho-S6RP(Ser240/244), Cell Signaling, catalog# 5364 (1:1000 dilution).<br>Vinculin, Abcam, catalog# ab-129002 (1:10000 dilution).<br>Rictor, Cell Signaling, catalog# 21145 (1:1000 dilution).<br>OxPhos (antibody cocktail) ,Thermo Fischer Scientific, catalog# 45-8099 (1:1000 dilution).<br>PLIN-2, Progen, catalog# GP40 (1:200 dilution).<br>Goat anti-Guinea Pig igG Alexa Fluor 555 conjugate, Thermo Fischer Scientific, catalog# A-21435 (1:1000)<br>TOMM20, Abcam, catalog# ab-186735 (1:1000 for western-blot applications, 1:100 for immunohistochemistry applications).<br>Prohibitin, Abcam, catalog# ab-28172 (1:200 dilution).<br>Goat anti-Rabbit IgG HRP conjugate, Bio-Rad, catalog# 1721019 (1:10000 dilution).<br>Goat anti-Rabbit IgG Alexa Fluor 555 conjugate, Thermo Fischer Scientific, catalog# A-21428 (1:1000 dilution).
Validation	AKT, Cell Signaling, catalog# 9272- validation provided on the manufacturer's website. Knockdown of AKT with siRNA in CHO cells yields a very faint band at the 60 KDa mark.<br>Phospho-AKT(Thr308), Cell Signaling, catalog# 4056- Internally validated, insulin treatment significantly enhances the expected band at the 60 KDa mark in hepatocytes and liver tissue from mice.<br>Phospho-AKT(Ser473), Cell Signaling, catalog# 9271- Internally validated, insulin treatment significantly enhances the expected band at the 60 KDa mark in hepatocytes and liver tissue from mice.<br>FOXO1, Santa Cruz Biotechnology, catalog# sc-11350 - Internally validated, band intensity vanishes at the 75 KDa mark in liver tissue

from FOXO1 KO mice.
Phospho-FOXO1(Ser256), Cell Signaling , catalog# 9461- Internally validated, band intensity vanishes at the 75 KDa mark in liver tissue from FOXO1 KO mice. In addition, insulin treatment significantly enhances the expected band also in hepatocytes and liver tissue from mice.
mTOR, Cell Signaling, catalog# 2983 - validation provided on the manufacturer's website. HeLa cells transfected with a siRNA against mTOR show a much fainter band above the 200 KDa mark than cells transfected with siRNA control.
Phospho-mTOR(Ser2448), Cell Signaling, catalog# 5536 - validation provided on the manufacturer's website. NIH/3T3 cells incubated with insulin show a much stronger band above the 200 KDa mark than untreated cells. When the cells are incubated with insulin and a phosphatase, the band is no longer visible. Also internally validated as insulin treatment enhances the expected band above the 200 KDa mark in primary hepatocytes and liver tissue from mice.
4E-BP1, Cell Signaling, catalog# 9452- validation provided on the manufacturer's website. HeLa cells with a targeted mutation in the gene encoding 4E-BP1 show change in 4E-BP1 molecular weight.
Phospho-4E-BP1(Thr37/46), Cell Signaling , catalog# 2855- internally validated, insulin treatment enhances the expected band between the 15 and 20 KDa marks in hepatocytes and liver tissue from mice.
S6 Ribosomal Protein, Cell Signaling, catalog# 2217- Antibody used in more than 2500 studies as reported on the manufacturer's website, it yields a distinct band below the 37 kDa mark, as expected.
Phospho-S6RP(Ser240/244), Cell Signaling, catalog# 5364- internally validated, insulin treatment enhances the expected band below the 37 KDa mark in hepatocytes and liver tissue from mice.
Vinculin, Abcam, catalog# ab-129002- Antibody consistently and satisfactorily used as loading control for liver tissue samples in our laboratory, as a reference please see https://doi.org/10.1016/j.cmet.2022.09.022. It gives a reliable band above the 100 kDa mark that do not vary in response to different treatment conditions as can be appreciated in the present study.
Rictor, Cell Signaling, catalog# 21145- internally validated, knockdown of Rictor with siRNA severely diminishes the expected band below the 250 KDa mark in hepatocytes and liver tissue from mice.
OxPhos (antibody cocktail) ,Thermo Fischer Scientific, catalog# 45-8099- internally validated, abundance of proteins from the mitochondrial complexes is much higher in lysates from hepatic mitochondrial extracts than from whole liver lysates in mice. Also validated in PMID: 38367663, that shows marked reduction in the abundance of proteins from mitochondrial complexes of brown adipose tissue in UCP1-KO mice as compared to control in response to cold exposure.
PLIN-2, Progen, catalog# GP40- internally validated, the intensity of the staining is directly proportional to the lipid content in hepatocyes in culture.
TOMM20, Abcam, catalog# ab-186735- validations provided on the manufacturer's website. Intensitiy of the expected band at the 15 KDa marker is much stronger in mitochondrial than in cytoplasmic fraction of HeLa cells.
Prohibitin, Abcam, catalog# ab-28172- this antibody has previously used by our group as a marker of mitochondrial mass, please see https://doi.org/10.1038/s42255-019-0124-x.

Eukaryotic cell lines

Policy information about cell lines and Sex and Gender in Research

Cell line source(s)	HEK293A cells (genetically female) were obtained from Life Technologies (catalog number R705-07).
Authentication	The cell lines used were not authenticated.
Mycoplasma contamination	The cells were not tested for mycoplasma contamination.
Commonly misidentified lines (See ICLAC register)	No misidentified lines were used in the study.

Animals and other research organisms

Policy information about studies involving animals; ARRIVE guidelines recommended for reporting animal research, and Sex and Gender in Research

Laboratory animals	Young male and female C57BL/6J mice (6 weeks of age) were obtained from Charles River Laboratories (Sulzfeld, Germany). Mice were acclimated to our animal housing facility for four weeks before taking part in the experiments, unless stated otherwise. The animals were housed 2-3 mice per cage and maintained with free access to tap water and standard chow (D12102C; Research diets, New Brunswick, NJ) under a 12:12-h light-dark cycle with lights on at 0600 and at controlled room temperature (20–22°C) and humidity conditions (50±15%). Cages were provided with bedding and nesting material, hiding structures and chewing implements to enhance environmental enrichment.<br>As a general rule, samples were collected at ZT2 as a representation of a postprandial state, while food was removed at ZT3 and then samples collected at ZT9, as a representation of a fasting state.
Wild animals	No wild animals were used in the study.
Reporting on sex	Female mice were used in only one study designed to evaluate the sex-dependent effects of hepatocyte-specific repression of the miR-379/miR-410 cluster in animals fed regular chow, for the rest of the studies only male mice were used.
Field-collected samples	No field-collected samples were used in this study.
Ethics oversight	All experimental procedures were carried out in accordance with European Union guidelines regarding the use of animals for

Ethics oversight	experimental purposes (Council Directive CEE 86/609) and were approved by the Government of Upper Bavaria (Regierungspräsidium Oberbayern; ROB-55.2-2532.Vet_02-15-164, ROB-55.2-2532.Vet_02-16-117, ROB-55.2-2532.Vet_02-21-66, ROB-55.2-2532.Vet_02-17-49).

Note that full information on the approval of the study protocol must also be provided in the manuscript.

Plants

Seed stocks	*Report on the source of all seed stocks or other plant material used. If applicable, state the seed stock centre and catalogue number. If plant specimens were collected from the field, describe the collection location, date and sampling procedures.*
Novel plant genotypes	*Describe the methods by which all novel plant genotypes were produced. This includes those generated by transgenic approaches, gene editing, chemical/radiation-based mutagenesis and hybridization. For transgenic lines, describe the transformation method, the number of independent lines analyzed and the generation upon which experiments were performed. For gene-edited lines, describe the editor used, the endogenous sequence targeted for editing, the targeting guide RNA sequence (if applicable) and how the editor was applied.*
Authentication	*Describe any authentication procedures for each seed stock used or novel genotype generated. Describe any experiments used to assess the effect of a mutation and, where applicable, how potential secondary effects (e.g. second site T-DNA insertions, mosiacism, off-target gene editing) were examined.*
