# Supplementary material for: Combinatorial targeting of the miR-379/miR-410 cluster normalizes glucose and lipid homeostasis in male models of diabetes and obesity
Source: Nat Commun. 2026 Sep 21;17:10014. doi: 10.1038/s41467-026-77808-2 (PMC13594115; doi:10.1038/s41467-026-77808-2)
Supplement: Supplementary file 3 [file 41467_2026_77808_MOESM3_ESM.pdf]

nature portfolio

Peer Review File

Combinatorial targeting of the miR-379/miR-410 cluster normalizes glucose and lipid homeostasis in male models of diabetes and obesity

Corresponding Author: Professor Stephan Herzig

This file contains all reviewer reports in order by version, followed by all author rebuttals in order by version.

Version 0:

Reviewer comments:

Reviewer #2

(Remarks to the Author)
For the authors,

In their study, Manuel Gil-Lozano et al. demonstrated that the miR-379/miR-410 cluster is elevated in the liver of people with obesity and diabetic mice. They used TuD inhibitors targeting the entire cluster to effectively improve lipid and glucose metabolism. By expressing decoy sequences to suppress miRNAs in the liver, the authors achieved significant reductions in circulating levels of triglycerides, total cholesterol, and LDL cholesterol. Moreover, they observed a decrease in basal glycemia along with improved glucose tolerance and insulin sensitivity. The authors argued that decoy therapy represents an innovative approach for addressing the pathophysiology of diabetes and obesity.

As a therapeutic approach, the novelty of using miRNA decoys to suppress diabetes and other metabolic abnormalities is worth revisiting. The authors describe it as “unprecedented,” citing as reference 43 the paper “J. Xie et al. Nature Methods 9(4), 403-409 (2012),” which was published 11 years ago and deals with miR-122. This study shares numerous similarities with the one described in the manuscript submitted by the authors, as both involved miRNAs known to some extent for their association with metabolic regulation.

This report is noteworthy because it indicates no significant side effects, including liver damage, and discusses the potential of miR-379 as a therapeutic target. However, the paper requires a more in-depth assessment of previous literature and detailed explanation of the underlying mechanism of the treatment effects as well as a comprehensive discussion to instill confidence in the authors’ treatment. Addressing these points will significantly strengthen the manuscript.

Major points
1. In Figure 1, the authors demonstrated the relationship among BMI, triglycerides, HOMA-IR, and miR-379 and miR-541 in humans. The relationship between miR-379 and BMI appeared to be significant, but modest. Additionally, miR-541 showed almost no correlation with triglycerides or HOMA-IR. Why did the authors group miR-379 and miR-541 together solely on the basis of their proximity to each other on the genome? It is not clear from the authors’ data whether the proximity of these two miRNAs has any meaningful effect; for example, anything of significance indicating that they share regulatory processes.

2. The authors reported an increase in Dlk1 expression in the liver under metabolic stress. However, it is worth noting that within the same locus, genes encoding molecules such as Gtl2, Rlt1, and Dlo3 exist, even though they may have different paternal or maternal origins. The miR-127/miR-136 and snoRNA miR-379/ miR-410 clusters encompass various families, and their regulation appears to involve relatively uniform upstream control mechanisms. Nevertheless, because individual correlations in expression or transcriptional control relationships with neighboring molecules have not been demonstrated, it remains unclear why only these two miRNAs are increased, given the lack of evidence in different individuals.

3. Specifically, is there anything we can consider regarding the regulation of miR-379/miR-541 expression? For example, are there any binding factors upstream of miR-379/miR-541 in databases such as ChIP-Atlas?

4. The authors demonstrated miR-379/miR-541 expression in the liver and suggested "hepatocyte-specific" intervention in the treatment sections. However, they did not provide evidence that hepatocytes are the primary cells expressing miR-379/miR-541 in liver tissues. Additionally, it is essential to determine whether there are age-related differences in liver miR-379/miR-541 expression between young and old mice. Furthermore, how does the liver expression of miR-379/miR-541 differ between fasting and refeeding conditions? Answering these questions would significantly enhance the informativeness of their study.

5. In Figure 1C and D, the authors observed an increase in miR-379/miR-541 due to steroids. Because steroid hormones vary diurnally, the authors need to investigate whether the expression of miR-379/miR-541 varies diurnally. Another approach is to compare the liver tissue expression of miR-379/miR-541 at different circadian time points.

6. The authors showed that miR-379/miR-541 levels are elevated in human patients with obesity, glucocorticoid-treated mice, high-fat diet-fed mice, and STZ-challenged mice. However, the authors did not collate the various miRNAs in the miR-379/miR-410 cluster in each figure. According to a previous report (M Kato et al., Nature Communications 7, 12864 (2016)), other miRNAs appear to be regulated by lnc-MGC, in addition to the ones presented by the authors. To better illustrate this, the authors should include a figure showing the genome structure of the miR-379/miR-410 cluster, including the relationship between lnc-MGC, the miRNAs that comprise the miR-379/miR-410 cluster, and Dlk1, similar to Figure 1c in the Kato et al. article. Accordingly, the authors should collate and show the miRNAs that make up the miR-379/miR-410 cluster, in addition to lnc-MGC and miR-379/miR-541, as shown in Figures 1 and 2 of the Kato et al. paper.

7. The authors convincingly demonstrated through several figures that their proposed decoy therapy indeed has cholesterol- and blood glucose-lowering effects in animals fed a normal diet. They also demonstrated the impact of decoy therapy on signal transduction within liver cells and mitochondria. Furthermore, the study showed the effectiveness of decoy therapy in improving metabolism not only in an obesity-induced hyperglycemia model but also in a type 1 diabetes model. However, the underlying mechanism of how suppressing multiple miRNAs with decoy therapy exerts therapeutic effects is not elucidated by the authors' data, with the study remaining at the level of observation. This is a significant weakness of this paper, because throughout the history of diabetes treatment, insulin therapy has essentially been the only treatment with a remarkable effect in ameliorating both type 1 and type 2 diabetes (often obesity-induced hyperglycemia). This is due to the fact that although both conditions share the same disease name, their onset mechanisms are quite different. If the authors claim that decoy therapy has significant effects on both types of diabetes, a thorough explanation of how this is possible is inevitably expected. Additionally, there is a lack of sufficient data on the impact of creating and investigating decoys individually for each miRNA rather than targeting multiple miRNAs simultaneously.

8. Each miRNA should have potential targets predicted on the basis of its sequence, and it should be possible to predict how it directly interacts with specific molecules. The authors should perform the analysis. Although they mentioned improvements in both glucose and lipid metabolism, it is important to consider that these processes may be independently regulated by miRNAs. Beyond these two miRNAs, it should be possible to demonstrate how the miRNAs within this cluster are distributed and target different sites. In particular, looking at the TuD results (Figure 2A-B), the effects appear to involve multiple miRNAs; therefore, it would be more informative to identify the specific targets of each miRNA.

9. The inhibition of miRNA function using antisense oligonucleotides or TuD was achieved by targeting both miR-541 and miR-379. Although this approach may have significant implications for demonstrating the ultimate improvement effect, it remains unclear whether there are molecularly independent targets or whether the treatment quantitatively amplifies the impact on certain targets.

10. The authors showed that the inhibition of miR-379/miR-541 enhanced the uptake of lipoproteins in primary hepatocytes (Figure 3). Similarly, a previous report showed that the miR-379/miR-544 cluster is resistant to high-fat diet-induced steatosis (C. Cao et al., Frontiers in Cell and Developmental Biology 9, 720900 (2021)). Although the authors of this manuscript found no changes in ALT/AST and liver TG in regular chow diet-fed mice, they should evaluate whether miR-379/miR-541 inhibition induces liver inflammation/fibrosis, steatosis (hepatic TG accumulation), and increased plasma ALT/AST in STZ-challenged mice and high-fat/high-sucrose diet-fed mice.

11. In Figure 4, the authors proposed increased FGF21 secretion from the liver as a possible mechanism for improving insulin resistance in muscle and adipose tissues. How do miR-379 and miR-541 increase hepatic FGF21 expression? Is it possible that miR-379 and miR-541 directly regulate FGF21 expression? A recent study showed that the inhibition of SLC25A47 in mitochondria led to decreased mitochondrial oxygen consumption, increased mitochondrial stress responses, and increased FGF21 expression in the liver (N. Bresciani et al., Journal of Hepatology 77, 1071-1082 (2022)). The authors should assess the effect of miR-379/miR-541 knockdown on mitochondrial oxygen consumption. The manuscript may be strengthened by including studies evaluating whether FGF21 inhibition (e.g., using antisense oligonucleotides) negates the protective effect of miR-379/miR-541 inhibition on insulin sensitivity.

12. Using western blot analysis, the authors identified the pathway leading to Akt phosphorylation and mTOR activation, presenting it as a liver-specific mechanism. However, it is important to consider that activation of the insulin signaling pathway, including IRS-PI3K, can be viewed as an aspect of improving systemic insulin resistance. As indicators of insulin resistance, activation of downstream pathways, including Akt, is expected not only in the liver but also in the muscle and adipose tissue. Therefore, the authors should provide evidence for this in their study. Consequently, it cannot be conclusively proven here that miRNAs interact directly with Akt and mTOR.

13. The authors proposed that decreased amino acid catabolism is a mechanism for reduced gluconeogenesis. Were the

glutamine/glutamate and methyl malonyl-CoA levels increase in the experiment shown in Figure 5D? Is it possible that these metabolites do not enter the TCA cycle, followed by gluconeogenesis?

14. Supplementary Figure 8C shows an increase in GKAP1 expression, which activates insulin signaling. Have the authors also investigated whether miRNAs in the miR-379/miR-410 cluster target GKAP1?

Minor points
1. In the Introduction section, the authors express the genomic position using the term "3' end." However, is it not more common to indicate whether it is on the telomeric or centromeric side?

2. The following is described in the Introduction section: "We also describe a novel approach to repress the entire miR-379/miR-410 cluster (the largest congregate of miRNAs in mammals) specifically in hepatocytes, by an AAV-delivered tough decoy (TuD), targeting distinct regions of the primary transcript of the cluster." Is it correct to describe the suppression of the entire cluster as a completely new approach? If this is completely new, then there is no need to add a reference, of course. However, if a previous report does exist, they should cite the paper and mention the advantages and disadvantages it pointed out.

3. It is wonderful that the authors have evaluated the proteome, but why did they not add RNA-Seq data as additional information on the effects of whole-cluster inhibition? Because miRNAs usually play a role in transcriptional interference, it may be worthwhile to examine their impact on overall gene expression.

4. In genomic analyses of human populations, such as GWAS, does the locus containing the targeted miRNA show strong associations with metabolic disorders, such as diabetes and hyperlipidemia?

5. Endoplasmic reticulum stress-CHOP has been shown to induce the expression of lnc-MGC (M Kato et al., Nature Communications 7, 12864 (2016)). Did ER stress increase in the authors' model in which miR-379/miR-541 was elevated?

6. The authors should provide more information in Figures 1 and 2, such as tissues (liver or adipose), treatment duration (4 weeks or three months), and species (humans or mice), for a better understanding of each figure.

7. In Figure 2, the authors showed reduced expression of lnc-MGC in the AAV-TuD miR-379/miR-541 group. Are there any previous reports on lnc-MGC knockout mice?

8. It is possible that I may be misinterpreting the data, but I do not believe it can be conclusively stated from Figure 2B that the effect of rAAV lasted for months. The figure does not appear to show a temporal alignment with the improvement in triglyceride levels for up to 5 weeks. If the authors want to demonstrate long-lasting effects, they should provide detailed evidence of the improvements in these phenotypes. Otherwise, there may be no need for a long-term focus in this paper.

9. In Figure 4I-J, TuD increased glucose uptake in the skeletal muscle and BAT. The increase in Fgf21 expression was notable but not very large. Please add additional discussions on the proteome or RNA-Seq data from the TuD-treated/control liver tissue (if any) and the decrease in adipose tissue mass (if any).

10. In these figures, the authors used both AAV-TuD and AAV-TuD 541/379 descriptions. Are there differences between the two? If not, the authors should standardize the descriptions to avoid confusion.

11. The authors should include a dot plot in the figures.

Reviewer #3

(Remarks to the Author)
Summary:
In "Combinatorial targeting of the miR-379/miR-410 cluster normalizes glucose and lipid homeostasis in diabetes and obesity," Gil-Lozano and colleagues demonstrate that microRNAs from the miR-379/miR-410 cluster, known to be important in pre-natal development, are also important for post-natal liver metabolism. Specifically, the authors identify upregulation of miR-379 and miR-541 in humans with obese BMIs. The authors design and test both an LNA-based antisense oligonucleotide and TuD inhibitor targeting miR-379 and miR-541 to elucidate the biological impacts on hepatic miR-379/miR-410 and subsequent metabolic endpoints, finding that the reduction of these microRNAs improved glucose homeostasis. Ultimately, the authors test the functional consequences of the TuD in vivo, targeting hepatic tissue in STZ and diet-induced obese male mice and presenting impressive data in Figure 7 that suggests the miR-541 and miR-379 TuDs ability to counteract metabolic hallmarks in mouse models of both Type 1 diabetes and diet-induced obesity. Collectively, the data presented demonstrate a novel decoy mediated approach to alter common metabolic endpoints associated with type 1 diabetes and diet-induced obesity.
While the data are intriguing, there are several aspects of this study that would benefit from further investigation and/or clarification, as described below:

Major Points:
- While single cell sequencing seems beyond the scope of the studies, it is unclear from the results whether the increase in

microRNAs arise from an increase in expression in one cell type (e.g. hepatocytes) or increased relative abundance of other cell types in the liver. Is tissue available for IHC studies?
- The amount of total RNA for microRNA studies, 10 ng, is a very low amount, and the microRNAs in question typically have very low expression in the adult liver. Since everything is normalized to a control value of “1”, it is hard to know the absolute amount of these microRNAs and whether the fold-change is meaningful, especially given the low number of human samples that were evaluated. Validation through another mean (e.g. northern blot) would be preferable.
- The data reporting and experimental design considerations are not in alignment with the Nature Portfolio guidelines on sex. For the human data in Supplementary Table 1, the data regarding the sex of human samples is omitted. This information should be included, as sex as a biological factor is important, especially in the obesity and diabetes literature. Furthermore, only male mice were used in the in vivo portion of the work. While the findings presented are impressive, the investigation of sex as a biological factor in these experiments is important prior to publication, or the title and resulting manuscript should be revised to clearly reflect that this work was only completed in male mice, as the current language in the manuscript is indicative of the data being applicable to both males and females, and thus, very misleading.
- Line 152: A 6h fast is mentioned, as well as “increased basal glycemia in morning fed animals.” Does morning refer to lights on? Throughout the manuscript is it not abundantly clear what time of day the mice were fasted, expect for the data illustrated in Figure 3A. As rodents are nocturnal and consume most of their calories during the night (and have the greatest physical activity), this information is vital and must be explicitly stated in the methods for each experiment. Additionally, it is not clear if chewing and/or running enrichments were present in the home cage environment.
- Line 214: The authors claim no obvious toxic effects were observed, but only body weight and water intake data were assessed. A more thorough toxicology panel is needed to support this claim.
- Figure 6B and C: Is there a way to label hepatocytes in these samples? The data from Figure 6A is hepatocyte specific, while these figures examine general liver tissue. Alternatively, would it be possible to complete these stains in cultured hepatocytes for consistency?
- It is not clear why diabetic patients (Type 1 and 2) are not included in the experiments used to generate Figure 1. In addition to a DIO group that receives treatment after the phenotypic change in the final in vivo experiments, this information would better inform the STZ data presented in Figure 7 (ie are miR-379 and miR-541 dysregulated in Type 1 and II diabetes, and if so, what are the implications for this in a mouse model?) This data would significantly strengthen the pre-clinical TuD experiments presented in Figure 7.
- MEG3 is implicated in obesity and Type 2 diabetes. Therefore, the authors should verify that the TuD does not target MEG3 expression as completed in the LNA-based antisense oligonucleotide experiments (Supplemental Figure 2G).
- The authors should include individual values in the bar graphs to show within group variability throughout the paper. This data presentation is necessary to inform the conclusions drawn.

Minor Points:
- Lines 58-61 could use additional clarity. Specifically, this section would benefit from the explicit mention of the miRNAs known to be important for insulin signaling in the liver.
- Line 65: It would be better to refer to this cluster as being expressed in eutherian mammals (i.e. with a placenta).
- Line 72: Please refer to the official HUGO gene nomenclature name MEG8 rather than lncMGC.
- Line 90: Please define VLDL in its first utilization.
- Line 113: The human nomenclature with all-capital letters (DLK1-DIO3) would be preferable here since the locus is being studied in human samples
- Line 117: Please define HOMA in its first utilization.
- Line 120: Please stay consistent- use either percentage or fold change to describe differences in miR-379 and miR-541 levels.
- Supplemental Figure 1 E & G: It is not clear why scWAT and skeletal muscle tissue samples were not collected for the corticosterone experiments, but were collected in the dexamethasone experiments to measure relative abundance of miR-379 and miR-541 levels. These tissue types should be presented in the corticosterone experiments. Additionally, the figure legend should clearly specify the experiments completed in mice versus humans.
- Supplemental Figure 1 D-G: Tissue abbreviations should be defined in the figure legend.
- Lines 124-136: The authors claim strong association between miR-541 and corresponding insulin, cortisol, and leptin levels. However, there is no linear regression presented to back this claim.
- Line 144-45: Please explicitly state the name of the liver-specific promoter and which rAAV vector capsid is used.
- Lines 144-145: When describing the viral construct, the specific AAV serotype and promoter should be mentioned here. The information is present in the methods, but should be made clear when initially described.
- Supplemental Figure 2B: It is not clear why Mirg expression levels were not evaluated in the epidydimal while adipose tissue, as this data is meant to reinforce the findings in Supplemental Figure 2A.
- Lines 164-165: As the dose response data for lncMGC is shown despite no effect, it would be advantageous for the authors to also include the dose response data for miR-379 and miR-541 levels.
- Line 163: “…without affecting other non-coding RNAs from the cluster.” The data presented only includes Mirg, to make this statement the other miRNA transcripts should be presented.
- Supplemental Figure 2G: Ascending values would be more useful here- 1 mg/kg looks like there might be a trend toward an effect, or great variability. Individual values should be graphed to address this point.
- Line 180: Clarify that the TuD tests in vitro were completed using plasmid constructs, and that the TuD was packaged in an AAV8 for in vivo work later described.
- General: italics for in vitro and in vivo.
- The sentences on lines 189-191 and 209-213 are confusing. Consider editing for clarity.
- Line 192: This sentence is misleading, as many factors could have contributed to lack of lncRNA knockdown including bioavailability and target binding engagement, not just nuclear localization.
- Line 208: Protein was examined for RICTOR and mRNA examined for Atf3. It would be advantageous to also include

protein data for ATF3 and transcript expression data for Rictor for consistency.
- Supplemental Figure 4 H and I: Ladders with molecular weights should be shown on the western blot images.
- Figure 2A and 2B should be labeled as 4 weeks and months, respectively.
- Line 216: Supplementary Figure 5B, no change in body mass observed over 13 weeks, however, it looks like there is a start of a divergence (TuD mice weight less). Would be interesting to see this with HFD.
- Line 222: The Phenomaster system data should be shown, even if no significant changes were observed. Were the mice single housed in this system? The methods mention that the mice were group housed in home cages, however, there are known behavioral changes when mice are single housed versus group housed.
- Supplementary Figure 6 C and D: could use additional labels of 5 weeks and months
- Line 247: Does 6h fasting reduce blood glucose concentrations at the month mark? This would be a better comparison for the data mentioned in Figure 4D, which examines pyruvate tolerance up to months. Additionally, the text states that "The decrease in blood glucose concentrations after a short period of fasting (6 h) was maintained for 5-6 weeks (Fig.4B)," yet Figure 4B only shows data up to 5 weeks.
- Figure 5G: Missing label for the bar graph groups.
- Data in Figure 7 are interesting. Not in the scope of this paper, but it would be interesting to see 1) the mitochondrial experiments shown in Figure 6 completed on the in vivo STZ and DIO tissues generated in Figure 7 to elucidate a potential mechanism, and 2) small RNA sequencing and bulk RNA sequencing of the liver tissues of these mice to assess potential adaptions in response to the TuD inhibitor.
- Line 411: This sentence is misleading because only a few microRNAs were tested, not the whole cluster and not all other tissues were evaluated. Would suggest removing it.
- Line 428 is also hyperbolic in nature.
- Line 532: Clarify the ns for each luciferase assay experiment ("at least 4 times" is vague).
- Methods section should include a section about the Phenomaster experiments.
- STZ mice were assessed for 9 weeks, DIO mice were assessed for 5 weeks. Why were these time courses not consistent?
- One or two tailed t-tests in statistics section? The statistical methods section could use additional detail and clarification.
- Scale bars should be provided on images in figures 3D, 3E, 6B and 6C.
- Protein molecular weights should be provided on figures 5A-C and E-G.
- More details should be provided on the gene expression assay section in the methods.

Version 1:

Reviewer comments:

Reviewer #2

(Remarks to the Author)
I would like to express my appreciation for the revised manuscript and to thank the authors for the extensive additional work, including the FGF21 knockout (KO) experiments, RNA seq, proteomics, and the revisions to the explanatory text. However, I still have a few concerns.
The authors report mild but significant increases in hepatic transaminases and fat accumulation following TuD treatment in the diet induced obesity (DIO) model. While they attribute discrepancies with previous reports to differences in the KO model, this explanation seems insufficient. The manuscript would be strengthened by additional discussion to more fully address this point.
In addition, some sentences remain rather long. I kindly recommend making certain descriptions more concise, while elaborating a little more carefully on the key arguments where appropriate.

Reviewer #3

(Remarks to the Author)
Gil-Lozano et al. completed additional work to address considerable comments from both reviewers in the manuscript titled "Combinatorial targeting 1 of the miR-379/miR-410 cluster normalizes glucose and lipid homeostasis in male models of diabetes and obesity." The authors clarified points that previously lacked detail in the manuscript, such as animal experiment design and potential toxicity of the TuD by completing additional imaging for liver inflammation in treated and control mice. In response to questions regarding sex as a biological factor, in the revised manuscript the authors included data in female mice for the TuD chow studies and clarified sample sex in the clinical dataset. Additionally, it was clarified that only cells with distinctive hepatocyte morphology were considered in these analyses and the authors completed studies to parse apart the role of glucocorticoids on the hepatocyte fraction relative to the non-parenchymal cell fraction. These efforts improved general data consistency throughout the manuscript.

Major points:
1. The premise of this work relies upon the assertion that miR-379 and miR-541 are upregulated in the liver of clinical population members with an obese phenotype. During the initial review, information regarding the sex of the clinical study participants was requested, as this information was previously not included in the first draft of the manuscript. Sex as a biological factor is important (SAGER guidelines) and is known to influence energy homeostasis, body weight, and miRNA expression (Mauvais-Jarvis 2015, Link et al. 2017, Sharma & Eghbali 2014). In the revisions, the authors did provide

information on human donor sample sex in Supplemental Table 1. This data revealed that non-obese males and 2 non-obese females were included in the control group, and 0 obese males and 11 obese females were included in the obese group- both sexes were used for controls, but only females were used for obese cases in the analysis. As mentioned, sex is known to play a critical role in miRNA expression and key sex differences exist in obesity. There is no discussion of this study design issue in the text and the subsequent analyses (Figure 1) are not balanced if only females are represented in the obese cohort. This represents a significant study design limitation. The authors could address this issue by including obese male cases in the data analysis, or by restricting the analysis to only female participants and clearly stating in the manuscript text and all associated figured that this data only applies to females ("obese human subjects" as currently stated in the current text would need to be changed to "obese female human subjects," for example).

2. The in vivo portion of this study sought to determine if a tough decoy (TuD) against miR-379 and miR-541 could impact metabolic phenotypes in mice. The authors did replicate the initial study to determine TuD efficacy in chow fed male and female mice, which provided new information to the study, including the observation of a significant difference in plasma cholesterol response to TuD therapy in female mice relative to male mice. The authors did not replicate the models of diabetes or prediabetic diet-induced obesity studies in female mice, and revised the study's title accordingly. In the methods section (Line 640), it is stated that "Young male and female C57BL/6J mice (6 weeks of age) were obtained from Charles River Laboratories (Sulzfeld, Germany)." The authors need to clarify that both sexes were only obtained for the chow experiments, and it needs to be made explicitly clear in the manuscript text and figures/figure legends if only male mice were utilized for the given experiment. Ideally, the diabetes experiments should be replicated in female mice. If this is not feasible, the authors should provide additional discussion of this serious limitation of the studies in the manuscript.

Version 2:

Reviewer comments:

Reviewer #2

(Remarks to the Author)
I would like to commend the authors for their substantial and rigorous revision of the manuscript. The additional experiments and analyses, including the Fgf21 knockout studies, transcriptomic and proteomic analyses, validation of liver-specific signaling pathways, and mitochondrial functional assays, have significantly strengthened the mechanistic depth and overall rigor of the study. In my view, most of the previous major scientific concerns have now been satisfactorily addressed.
The authors' response to the concerns regarding sex as a biological factor is also appropriate and transparent. The inclusion of a larger validation cohort comprising both male and female human subjects supports the conclusion that hepatic upregulation of miR-379/miR-541 is associated with obesity rather than being primarily sex-dependent. The authors also clearly acknowledge the limitation that the rodent disease models of diabetes and diet-induced obesity were examined only in males.
One remaining issue concerns the framing of safety. In the DIO model, TuD treatment is associated with increased hepatic steatosis and mild but significant elevations in circulating ALT and AST. The additional discussion explaining these findings in relation to hepatic lipid flux and differences between the hepatocyte-specific TuD model and previous constitutive knockout models is helpful and biologically plausible. However, the current safety framing still appears somewhat too strong. In particular, the abstract states that the treatment produced metabolic benefits "with no discernable side effects," and the conclusion refers to an "apparent lack of toxicity." These statements are not fully consistent with the DIO safety data.
I therefore recommend that the authors revise the abstract, summary/conclusion, and relevant discussion to explicitly acknowledge the mild hepatic safety signal observed in the DIO model. The manuscript should make clear that no overt toxicity was observed under chow-fed conditions, but that increased hepatic fat accumulation and transaminase elevations were detected under diet-induced obesity. The potential implications for translation to obese, insulin-resistant, or fatty liver disease contexts should also be discussed more carefully.
In addition, there appear to be inconsistencies in the supplementary figure numbering. The STZ-related supplementary data are cited in the main text as Supplementary Fig. 16, whereas the supplementary material appears to present these data as Supplementary Fig. 17. Similarly, the DIO-related data are cited as Supplementary Fig. 17, while the supplementary material appears to present them as Supplementary Fig. 18. These cross-references should be corrected before publication.

Reviewer #3

(Remarks to the Author)
I appreciate the additional work the authors completed to ensure sex differences were accounted for in the human portion of this manuscript. The new insights into miR-379 and miR-541 in both male and female obese and non-obese study participants strengthens the manuscript.

I still have considerable concerns about female mice not being included in the in vivo models of diet-induced obesity and STZ-induced diabetic models. I acknowledge that the authors provided additional clarifications within the manuscript text and discussion regarding the limitations of not studying sex differences in these models. However, I strongly disagree with the notion that "we are confident that improvements in glucose and lipid homeostasis are also expected in female models of obesity and diabetes in response to such manipulation." To be confident in this outcome, the work must be completed, especially since there are 1) profound metabolic differences in the STZ-induced and diet-induced obesity mouse models compared to mice fed standard rodent chow and 2) marked differences in metabolism between the sexes. For publication, I would strongly recommend that these experiments be completed, as sex as a biological variable is of preclinical

significance.

Open Access This Peer Review File is licensed under a Creative Commons Attribution 4.0 International License, which permits use, sharing, adaptation, distribution and reproduction in any medium or format, as long as you give appropriate credit to the original author(s) and the source, provide a link to the Creative Commons license, and indicate if changes were made.
In cases where reviewers are anonymous, credit should be given to 'Anonymous Referee' and the source.
The images or other third party material in this Peer Review File are included in the article's Creative Commons license, unless indicated otherwise in a credit line to the material. If material is not included in the article's Creative Commons license and your intended use is not permitted by statutory regulation or exceeds the permitted use, you will need to obtain permission directly from the copyright holder.
To view a copy of this license, visit https://creativecommons.org/licenses/by/4.0/

REVIEWER COMMENTS

Reviewer #2 (Remarks to the Author):

We appreciate the reviewer's most helpful comments.

For the authors,

In their study, Manuel Gil-Lozano et al. demonstrated that the miR-379/miR-410 cluster is elevated in the liver of people with obesity and diabetic mice. They used TuD inhibitors targeting the entire cluster to effectively improve lipid and glucose metabolism. By expressing decoy sequences to suppress miRNAs in the liver, the authors achieved significant reductions in circulating levels of triglycerides, total cholesterol, and LDL cholesterol. Moreover, they observed a decrease in basal glycemia along with improved glucose tolerance and insulin sensitivity. The authors argued that decoy therapy represents an innovative approach for addressing the pathophysiology of diabetes and obesity.

As a therapeutic approach, the novelty of using miRNA decoys to suppress diabetes and other metabolic abnormalities is worth revisiting. The authors describe it as "unprecedented," citing as reference 43 the paper "J. Xie et al. Nature Methods 9(4), 403-409 (2012)," which was published 11 years ago and deals with miR-122. This study shares numerous similarities with the one described in the manuscript submitted by the authors, as both involved miRNAs known to some extent for their association with metabolic regulation.

We are aware of the excellent and prolific work by the group of Guangping Gao in the field of gene therapy, including the pioneer study of Xie and colleagues on the use of tough decoys for the inhibition of microRNAs *in vivo*. We must emphasize that we do not intend to claim to be the first group to utilize the tough decoy technology *in vivo* and, accordingly, as the reviewer pointed out, we cite the manuscript by Xie and colleagues (reference #50 in our list) when we state that "AAV-delivered TuDs have been shown to promote long-term inhibition of miRNA function (*in vivo*)". As matter of fact, a growing number of studies have employed this technology for the inhibition of microRNAs in living animals. However, we respectfully disagree with the reviewer's opinion that the similarities with Xie's study subtracts novelty from our own work. While the work by Xie and colleagues is an excellent proof of concept study that validates the utilization of tough decoys for studies on microRNA function, the main focus of the study is to demonstrate the superior efficiency of TuD as inhibitors of miRNA activity with respect to alternative technologies, first *in vitro* and then validating these effects *in vivo.* Although the authors reproduce with the TuD inhibitors the effects promoted by antisense oligonucleotides directed against miR-122 on circulating cholesterol levels, their study is not intended to explore the biologic effects of any of the miRNA they target (*miR-122* and *let-7*). In contrast, in our study we use a TuD inhibitor as a valuable tool to investigate the metabolic implications of combined inhibition of two miRNAs simultaneously (with the same TuD inhibitor). Moreover, we report not only inhibition of the two target microRNAs in response to the TuD therapy, but also repression of other related miRNAs belonging to the same cluster, thus dramatically enhancing the metabolic effects promoted by antisense oligonucleotides directed against our two target miRNAs. As a result, multiple metabolic parameters are affected by our approach, and the reported metabolic improvements in glucose and lipid homeostasis might prove beneficial for the treatment of metabolic disorders such as obesity and diabetes. Accordingly, we demonstrate vast metabolic improvements in experimental models of prediabetic obesity and type I diabetes to back up this claim. Finally, our approach is highly liver specific, which clearly separates our work from previous functional studies involving

constitutive KO models of the same cluster of miRNAs. In essence, that is why we consider our approach unprecedented, because we demonstrate that liver specific targeting of a large cluster of microRNAs results in the improvement of multiple metabolic parameters, not only in healthy animals (supporting a role for the cluster of microRNAs in the regulation of hepatic metabolic homeostasis) but also in experimental models of obesity and diabetes.

We have now modified the last sentence of the abstract to emphasize that what we consider an unprecedented approach is not the use of TuD inhibitors *per se*, but rather the hepatocyte-specific combinatorial inhibition of a large miRNA cluster**.**

This report is noteworthy because it indicates no significant side effects, including liver damage, and discusses the potential of miR-379 as a therapeutic target. However, the paper requires a more in-depth assessment of previous literature and detailed explanation of the underlying mechanism of the treatment effects as well as a comprehensive discussion to instill confidence in the authors' treatment. Addressing these points will significantly strengthen the manuscript.

Major points

1. In Figure 1, the authors demonstrated the relationship among BMI, triglycerides, HOMA-IR, and miR-379 and miR-541 in humans. The relationship between miR-379 and BMI appeared to be significant, but modest. Additionally, miR-541 showed almost no correlation with triglycerides or HOMA-IR. Why did the authors group miR-379 and miR-541 together solely on the basis of their proximity to each other on the genome? It is not clear from the authors' data whether the proximity of these two miRNAs has any

meaningful effect; for example, anything of significance indicating that they share regulatory processes.

As mentioned throughout the text, previous reports of our group showed a role for hepatic *miR-379* in the regulation of circulating triglyceride levels. With this precedent, our goal in the present study was to identify other microRNAs from the DLK1-DIO3 locus with a potential role in liver metabolic homeostasis, more specifically in the regulation of hepatic insulin sensitivity and/or hepatic glucose output. With this aim, we investigated the levels of expression of different microRNAs from the DLK1-DIO3 locus in the livers of healthy volunteers and prediabetic obese patients with underlying insulin resistance. A large fraction of the microRNAs studied were found to be significantly upregulated in the livers of obese subjects (Supplemental Figure 1B), and of all the transcripts examined, *miR-379* and *miR-541* showed the most robust upregulation (Figure 1A and 1B). Importantly, a similar outcome was found in our studies with experimental models of hepatic insulin resistance, where, again, *miR-379* and *miR-541* came out as the two microRNAs with the most sensitive response to the different metabolic insults (as shown in new Figure 2). Given its strong regulation in humans and mice, we next investigated the role of miR-541 in liver metabolism by overexpressing this microRNA in a liver specific manner. The resulting increase in HOMA-IR, as well as circulating levels of insulin and glucose supported a role of miR-541 in the regulation of hepatic glucose homeostasis. These findings plus the previous established role of miR-379 in the regulation of lipoprotein uptake by the liver, lead us to believe that targeting both miRNAs simultaneously might induce effects on both lipid and glucose homeostasis. That is the main reason we initially focused on targeting these two microRNAs simultaneously. We have modified the text in order to make our intentions clearer for the readership.

The reviewer makes a great point inquiring about the regulatory processes of our microRNAs of interest. Previous studies have reported coregulation of the different components of the *miR-379/miR-410* cluster in response to various stimuli (please see our response to major point #3 for

a more detailed explanation), suggesting that they are expressed from the maternal chromosome as a long primary transcript that then gives yield to the different microRNAs. In kidney cells, this long primary transcript has been identified as lncMGC. Whether lncMGC is the only host transcript from which the different microRNAs are processed remains to be clarified, but some new reports showing expression of different microRNAs from the cluster in the absence of *lncMGC* (as discussed in more detail in our response to minor point #7) support that other host transcripts apart from *lncMGC* could be involved. One of these alternative primary transcripts might be Mirg, which is another lncRNA hosting many (although not all) of the miRNAs from the cluster and whose expression is also detected in the absence of lncMGC. In the revised version of our manuscript, we have added new data showing upregulation of different microRNAs of the cluster along with the host transcripts, *lncMGC* and *Mirg,* in response to glucocorticoid stimulation, supporting the hypothesis of coregulation of the cluster (please see our response to major point #2 below). The finding that *miR-379* and *miR-541* appear to be more sensitive to the different stimuli may coincide with a longer half-life of these transcripts or, perhaps, they are more actively processed from the primary transcript. We acknowledge that in the first version of our manuscript the regulation of the cluster was not covered in detail, we have now rectified this by adding a whole paragraph in the discussion dealing with this aspect. Finally, it is also likely that the regulation of the cluster may differ across species, as it is known that in humans the microRNAs from the cluster overlap with the sequence of two other lncRNAs, MEG8 and MEG9.

Regarding the reviewer's comment about the apparent lack of correlation between hepatic *miR-541* expression and circulating triglyceride levels and HOMA-IR values, respectfully, we must disagree. Although it is true that the pairwise associations between hepatic expression of *miR-541* and other variables, such as circulating levels of triglycerides and HOMA-IR values, do not seem to be linear, a closer examination of the data suggests that there is indeed an association. We have now studied this association using distance correlation, a measure that can detect both

linear and non-linear dependencies, which is more suitable for our data than the commonly used Pearson correlation and linear regression. A more detailed description of the analysis can be found in the statistical section of the methods. The distance correlation (r_d) and corresponding p-value for each pairwise association can be found in the legend of the corresponding graph. As an example, the distance correlation between *miR-541* levels and triglycerides is 0.76 (P=0.001), while the distance correlation between *miR-541* and HOMA-IR values is 0.61 (P=0.017). Furthermore, distance correlation is not restricted to analyzing pairs of single variables; it can also capture joint associations between groups of variables. Accordingly, the combined association of *miRNA-379* and *miRNA-541* with triglycerides and HOMA-IR yields a distance correlation of 0.687 (P= 0.008). Overall, this supports our claims of a strong association between the levels of expression of the microRNAs and the metabolic parameters considered.

2. The authors reported an increase in Dlk1 expression in the liver under metabolic stress. However, it is worth noting that within the same locus, genes encoding molecules such as Gtl2, Rlt1, and Dlo3 exist, even though they may have different paternal or maternal origins. The miR-127/miR-136 and snoRNA miR-379/ miR-410 clusters encompass various families, and their regulation appears to involve relatively uniform upstream control mechanisms. Nevertheless, because individual correlations in expression or transcriptional control relationships with neighboring molecules have not been demonstrated, it remains unclear why only these two miRNAs are increased, given the lack of evidence in different individuals.

Again, we would like to emphasize that not only *miR-379* and *miR-541* levels were increased, but also the levels of expression of other components of the *miR-379/miR-410* cluster were significantly elevated in the livers of obese subjects as well as of our experimental models of insulin resistance. As discussed above, and considering that all the microRNAs from the cluster are believed to derive from common long primary transcripts (at this point it is not possible to

determine whether there is only one or more), the observation that *miR-379* and *miR-541* appear to be more sensitive than other microRNAs from the cluster might be explained by differences in half-life or disparities on how these two microRNAs are processed from the common host transcripts. To provide a better understanding on how this cluster is regulated in the liver, we have now studied the response of different maternal non-coding RNAs to glucocorticoid stimulation, a factor known to regulate the *miR-379/miR-410* cluster at the transcriptional level (data shown in novel Figure 2A-D). According to the hypothesis of coregulation of the entire cluster, we observed upregulation of not only *miR-379* and *miR-541* to glucocorticoid stimulation, but also of other microRNAs from the cluster (*miR-382*, *miR-409*) as well as the host transcripts *lncMGC* and *Mirg*. Most interestingly, the expression of other lncRNA from the Dlk1-Dio3 locus upstream the *miR-379/miR-410* cluster (*Meg3*, also known as *Glt2*, and *Rian*) was either not affected or reduced by glucocorticoid stimulation, thereby demonstrating that the regulation of the *miR-379/miR-410* cluster is independent from the regulation of the non-coding RNAs in the proximal end of the locus. Supporting this, the expression of *miR-127,* a representative member of the *miR-127/miR-136* cluster also located on the proximal end of the locus, upstream of the *miR-379/miR-410* cluster, was also not affected by glucocorticoid treatment. Moreover, in contrast to our novel findings showing comparable basal expression of the microRNAs from the *miR-379/miR-410* cluster in hepatocytes and non-parenchymal cells from the liver, we observed that the expression of *miR-127* was markedly higher in non-parenchymal cells, indicating again major differences in the regulation of the two major clusters of microRNAs within the Dlk1-Dio3 locus (novel Supplementary Figure 2G and 2H). The results and the discussion sections have been modified in the light of these new findings.

3. Specifically, is there anything we can consider regarding the regulation of miR-379/miR-541 expression? For example, are there any binding factors upstream of miR-379/miR-541 in databases such as ChIP-Atlas?

Several binding sites for different transcription factors have been identified upstream the *miR-379/miR-410* cluster, including MEF2A, TGFB1, CHOP and glucocorticoid receptor. MEF2A was demonstrated to regulate the expression of this cluster in both neurons and skeletal muscle (please see references #37 and 45); TGFB1 and CHOP were found to regulate expression in kidney cells (please see reference #14); while our group has reported recruitment of the glucocorticoid receptor to binding sites upstream the *miR-379* sequence in hepatocytes (please see reference #21). Moreover, we have also previously demonstrated upregulation of *miR-379* in response to dexamethasone stimulation both in whole liver *in vivo* and in hepatocytes *in vitro*, an effect blunted by pretreatment with a glucocorticoid receptor antagonist and absent in animals unable to dimerize the receptor (reference #21). In addition, we have previously shown severe downregulation of *miR-379* hepatic levels in animals defective for the glucocorticoid receptor in a liver-specific manner (reference #21). Overall, these observations indicate that the *miR-379/miR-410* cluster represents a direct target of the glucocorticoid receptor in the liver. We have now complemented these findings by reporting upregulation of not only *miR-379*, but of other components of the *miR-379/miR-410* cluster (*miR-541*, *miR-382*, *miR-409*, as well as the host transcripts *lncMGC* and *Mirg*) in the liver of animals treated with dexamethasone for 48 hours or corticosterone for 4 wks (novel Figure 2A-D) and in primary hepatocytes in culture incubated with dexamethasone (new Supplementary Figure 6C and 6D). Moreover, novel studies performed by us (as discussed in our response to major point #4 below) also show that only the hepatocytes, and not the non-parenchymal cells of the liver, are sensitive to glucocorticoid stimulation (new Supplementary Figure 2G). Thus, demonstrating that the upregulation found in the whole liver is hepatocyte driven. We have now added a new paragraph in the discussion compiling this information regarding the regulation of the *miR-379/miR-410* cluster.

4. The authors demonstrated miR-379/miR-541 expression in the liver and suggested "hepatocyte-specific" intervention in the treatment sections. However, they did not provide

evidence that hepatocytes are the primary cells expressing miR-379/miR-541 in liver tissues. Additionally, it is essential to determine whether there are age-related differences in liver miR-379/miR-541 expression between young and old mice. Furthermore, how does the liver expression of miR-379/miR-541 differ between fasting and refeeding conditions? Answering these questions would significantly enhance the informativeness of their study.

Our claim on the hepatocyte-specific nature of our intervention relies on previous data showing that the LP1 promoter (the one controlling the expression of our TuD) drives expression solely in hepatocytes and not in non-parenchymal cells of the liver (please see reference #65). Moreover, in our present study, we report comparable knockdown of our non-coding RNAs of interest in response to the TuD in both whole liver tissue (new Figure 3A and 3B) and hepatocytes in culture (new Supplementary Figure 6C and 6D). Thus, supporting that the hepatocyte-specific knockdown is sufficient to explain the effects in whole liver tissue. However, we agree with the reviewer that our initial version of the manuscript lacked information on the expression of the *miR-379/miR-410* cluster in other cell types of the liver. We have now run liver fractionation studies and observed that the expression of microRNAs from the *miR-379/miR-410* cluster is comparable between hepatocytes and non-parenchymal cells. The study of cell specific markers allows us to discard any potential contamination of the hepatocyte fraction with non-parenchymal cells (new Supplementary Figure 2F). Most importantly, we also observed that treatment with glucocorticoids only increases the expression in the hepatocyte fraction, while the non-parenchymal cell fraction is insensitive to this stimulation (new Supplementary Figure 2G). This demonstrates that, at least in the models of glucocorticoid exposure, the upregulation of the microRNAs from our cluster of interest is, certainly, hepatocyte specific.

Previous studies show that the levels of expression of the non-coding RNAs from *the miR-379/miR-410* cluster decline after birth. We recognize these findings when we state in the discussion:

“Herein, we demonstrate that miRNAs from the *miR-379/miR-410* cluster are robustly upregulated in the liver of different experimental models associated with hepatic insulin resistance and increased hepatic glucose output, including animals on glucocorticoid therapy, treated with streptozotocin, or fed a NASH-inducing diet. Thus, demonstrating that, although the profile of expression of these miRNAs readily declines after birth, positive regulation also occurs in adulthood.”

In addition, we have now studied the levels of expression of these microRNAs in the liver of animals at different times across their life cycle: Postnatal day one (P1), at three months of age (representing the age of the animals routinely used in our studies), and at eight months of age (representing late adulthood). As expected, we observed extremely high levels of the microRNAs in neonatal animals that then rapidly decline with age. Interestingly enough, the basal expression at 3 months of age is comparable to the basal expression at 8 months of age, indicating that the expression of these non-coding RNAs stabilizes after the striking decline observed shortly after birth (results presented in novel Supplementary Figure 3D). In any case, this highly anticipated observation, of rapid decline in expression after birth, does not change our conclusions that the expression levels of these microRNAs are still responsive to metabolic insults in young adulthood, and that the expression can still be manipulated and further reduced by our interventions. If anything, these findings emphasized that experimental manipulation of these microRNAs is still possible in late adulthood.

Finally, we have also collected data on the levels of expression of our microRNAs of interest over a 24 h period (please see our response to the next comment below). Although we found a circadian variation in the levels of expression of the host transcript *Mirg* (Supplementary Figure 3B), we detected no significant differences in the levels of expression of *miR-379* or *miR-541* across the day (Supplementary Figure 3C), indicating that these microRNAs are not regulated by the feeding cycle. Accordingly, we also failed to detect differences in their hepatic levels of

expression between short-term (6 h) fasting and postprandial conditions (data not shown in the manuscript).

5. In Figure 1C and D, the authors observed an increase in miR-379/miR-541 due to steroids. Because steroid hormones vary diurnally, the authors need to investigate whether the expression of miR-379/miR-541 varies diurnally. Another approach is to compare the liver tissue expression of miR-379/miR-541 at different circadian time points.

The reviewer is right that we observed consistent upregulation of the *miR-379/miR-410* cluster in response to glucocorticoids both *in vivo* and *in vitro*. Nevertheless, we would like to point out that the *in vivo* effects were reported in response to prolonged exposure to pharmacological doses of glucocorticoids (both corticosterone and dexamethasone), which does not necessarily mean that the cluster will be equally responsive to physiological oscillations in corticosterone levels. However, in order to investigate whether the expression of the different components of the *miR-379/miR-410* cluster is regulated in a circadian manner, we have now run a novel circadian study collecting liver samples every 4 hours over a period of 24 hours. In this study (data presented on Supplementary Figure 3A-C), we found the expected circadian oscillations of two canonical clock genes used as positive controls, *Bmal1* and *Per2*. Interestingly, we also observed that levels of expression of the host transcript *Mirg* nicely match a 24 h period oscillation, resembling the daily fluctuations in corticosterone, with peak values at the end of the light period and trough values at the end of the dark period. In contrast, the daily expression of the mature microRNAs *miR-379* and *miR-541* failed to adjust to a 24 h period, maintaining constant levels of expression throughout the day. These findings are in agreement with previous reports on *miR-122*, showing daily oscillations of the precursor of the microRNA but not of the mature microRNA, likely because of the much longer half-life of the mature form (please see reference #22). This experiment also demonstrates that the levels of expression of the mature microRNAs do not vary with the feeding cycle, as commented above.

6. The authors showed that miR-379/miR-541 levels are elevated in human patients with obesity, glucocorticoid-treated mice, high-fat diet-fed mice, and STZ-challenged mice. However, the authors did not collate the various miRNAs in the miR-379/miR-410 cluster in each figure. According to a previous report (M Kato et al., Nature Communications 7, 12864 (2016)), other miRNAs appear to be regulated by lnc-MGC, in addition to the ones presented by the authors. To better illustrate this, the authors should include a figure showing the genome structure of the miR-379/miR-410 cluster, including the relationship between lnc-MGC, the miRNAs that comprise the miR-379/miR-410 cluster, and Dlk1, similar to Figure 1c in the Kato et al. article. Accordingly, the authors should collate and show the miRNAs that make up the miR-379/miR-410 cluster, in addition to lnc-MGC and miR-379/miR-541, as shown in Figures 1 and 2 of the Kato et al. paper.

According to the reviewer's suggestion, we have now included an illustration of the *Dlk1-Dio3* locus in new Figure 2E of the revised version of our manuscript. In this illustration, the genes expressed by the paternal and maternal chromosome are represented. We also show a magnified scheme of the *miR-379/miR-410* cluster including all the microRNAs comprising the cluster as well as their relative location. In addition, we highlight the sequences that our TuD recognizes within the cluster, *miR-379* and *miR-541* which are located on the proximal and distal ends of the cluster, respectively; this way, the readers can have a better visualization of what our inhibitor is targeting. Finally, we also represent the host transcripts currently identified in the cluster, *Mirg*, *B830012L14Rik* and *lncMGC*, as well as all the microRNAs contained in their sequence.

As it can be appreciated in the illustration, the number of microRNAs comprising the *miR-379/miR-410* cluster is ≈40. When we study the regulation of the cluster by glucocorticoids (new Figure 2A-D) or the repressive effects of the TuD therapy on the cluster (Figure 3A and 3B), we do not quantify the levels of expression of every single microRNA, but just a representative number of them. We use Taqman technology to measure the expression of the microRNAs as

opposed to the SybrGreen technology utilized in Kato's study. Taqman technology is more reliable and sensitive than SybrGreen technology, but it is also more expensive. Thus, studying the expression of every single component of the cluster with this technology would increase the cost of the study immensely. We feel that showing effects on a representative number of the microRNAs, as well as the host transcripts *lncMGC* and *Mirg*, is sufficient to give an impression on the general response of the cluster.

7. The authors convincingly demonstrated through several figures that their proposed decoy therapy indeed has cholesterol- and blood glucose-lowering effects in animals fed a normal diet. They also demonstrated the impact of decoy therapy on signal transduction within liver cells and mitochondria. Furthermore, the study showed the effectiveness of decoy therapy in improving metabolism not only in an obesity-induced hyperglycemia model but also in a type 1 diabetes model. However, the underlying mechanism of how suppressing multiple miRNAs with decoy therapy exerts therapeutic effects is not elucidated by the authors' data, with the study remaining at the level of observation. This is a significant weakness of this paper, because throughout the history of diabetes treatment, insulin therapy has essentially been the only treatment with a remarkable effect in ameliorating both type 1 and type 2 diabetes (often obesity-induced hyperglycemia). This is due to the fact that although both conditions share the same disease name, their onset mechanisms are quite different. If the authors claim that decoy therapy has significant effects on both types of diabetes, a thorough explanation of how this is possible is inevitably expected. Additionally, there is a lack of sufficient data on the impact of creating and investigating decoys individually for each miRNA rather than targeting multiple miRNAs simultaneously.

Although it is true that type 1 and type 2 diabetes are two distinct clinical entities, the first one characterized by deficient secretion of insulin while the second one by hyperinsulinemia-driven insulin resistance (although it can also progress to deficient secretion of insulin in the long-term), it is also true that both entities share a common feature, inability of the body (for one reason or another) to maintain glycemia within the normal range, eventually leading to severe complications, some of them life-threatening. In this regard, reduction of glucose levels by insulin-independent mechanisms, like decreasing hepatic glucose output with metformin, or enhancing renal clearance of glucose with SGLT2 inhibitors in patients with type 2 diabetes, as well as by following a carbohydrate-restrictive diet in patients with type 1 diabetes before insulin was available as treatment, has proven to be successful in both diseases. One of the major metabolic effects in response to the silencing of the *miR-379/miR-410* cluster in the liver is diminished hepatic glucose output. We have now added novel data showing that the TuD therapy reduces the expression of key gluconeogenic enzymes (*Pck1* and *G6pc*) in both diet-induced animals (as a model of hyperinsulinemic prediabetes obesity, novel Supplementary Figure18C) and STZ-treated mice (as a model of type 1 diabetes, novel Supplementary Figure 17A), likely due to the inhibition of FOXO1, a master regulator of the gluconeogenic program; although the derepression of Atf3, a target of the miRNAs from the cluster which competes with CREB to bind to same elements could also play a role. Reduction of glycemia by restraining hepatic glucose production is an approach that can benefit patients with either type 1 or type 2 diabetes. Moreover, improved insulin sensitivity in both liver and extrahepatic tissues is another major metabolic effect induced by the hepatic-specific repression of the cluster. Novel studies conducted by us demonstrate that this effect is partially mediated by FGF21 (as discussed in our response to major comment #11 below). It is worth noting at this point that, although widely regarded as a model of type 1 diabetes, STZ-treated animals are still able to maintain a certain level of insulin secretion by the pancreas (as shown in new Supplementary Figure 17C) and that they develop severe insulin resistance due to the persistent high levels of glycemia; improvement of insulin sensitivity greatly ameliorates

glycemic control in this model. In this regard, long-term insulin therapy is associated with the development of insulin resistance in patients with type 1 diabetes, improvement of insulin sensitivity and reduction of hepatic glucose output would likely have an impact on these patients that might be able to reduce their insulin dose and thus avoid some of the unwanted secondary effects derived from the long-term usage of high doses of insulin. We have now added a paragraph in the discussion dealing with the mechanisms underlying the effects of hepatocyte-specific silencing of the *miR-379/miR-410* cluster in glucose homeostasis, and how these effects are beneficial for both the diet-induced obese animals and the STZ-treated mice.

Finally, although we recognize that we failed to provide data on the effects of TuDs directed against one single miRNA, throughout the text we discuss that specific targeting of miR-379 promotes effects on triglyceride levels without affecting glucose homeostasis, and that the very specific targeting of miR-379 and miR-541 with our ASOS, reduces triglyceride levels and enhances insulin tolerance without affecting basal glycemia, glucose clearance and cholesterol levels. Only when we achieve a general repression of the cluster by targeting the host transcripts lncMGC and Mirg with our TuD the totality of metabolic effects is manifested. As a result, it can be predicted that the use of alternative TuDs also able to target these two host transcripts simultaneously would promote similar metabolic effects.

8. Each miRNA should have potential targets predicted on the basis of its sequence, and it should be possible to predict how it directly interacts with specific molecules. The authors should perform the analysis. Although they mentioned improvements in both glucose and lipid metabolism, it is important to consider that these processes may be independently regulated by miRNAs. Beyond these two miRNAs, it should be possible to demonstrate how the miRNAs within this cluster are distributed and target different sites. In particular, looking at the TuD results (Figure 2A-B), the effects appear to involve multiple

miRNAs; therefore, it would be more informative to identify the specific targets of each miRNA.

According to the suggestions of the reviewer, potential targets of each microRNA (not only of miR-379 and miR-541, but also of the other microRNAs from the cluster we have direct evidence to be downregulated by our TuD inhibitor, i.e miR-382, miR-134 and miR-409) were predicted by miRNA target prediction algorithms (http://mirwalk.umm.uni-heidelberg.de/). We followed a rather conservative approach and only genes predicted to be targeted by the same microRNA at multiple sites were considered as potential targets. Then, enriched pathway analyses of each gene list were run utilizing the KEGG pathway database (https://bioinformatics.sdstate.edu/go/). The 20 most highly enriched pathways for each microRNA are shown in novel Supplemental Figure 11. Of note, although there are some unique pathways for each microRNA that are not shared with the other microRNAs examined, some entries related to enhanced activity of PI3K/mTOR signaling such as "PI3K-AKT signaling pathway", "EGFR tyrosine kinase inhibitor resistance", "Insulin signaling", "Focal adhesion", "Regulation of actin cytoskeleton", "Ras signaling" and "Rap1 signaling" are among the most repeated entries across the different microRNAs; overall supporting a role for these microRNAs in the regulation of PI3K/mTOR signaling.

9. The inhibition of miRNA function using antisense oligonucleotides or TuD was achieved by targeting both miR-541 and miR-379. Although this approach may have significant implications for demonstrating the ultimate improvement effect, it remains unclear whether there are molecularly independent targets or whether the treatment quantitatively amplifies the impact on certain targets.

As discussed above, although each microRNA may have specific targets of its own, it is also true that, for the most part, they seem to target genes with related function (regulation of PI3K/mTOR signaling, for example). Thus, although the inhibition of each independent microRNA may have

relatively modest effects by itself, the inhibition of a large number of microRNAs sharing similar targets (as induced by our TuD inhibitor) will, more than likely, amplify these effects by synergism. That probably is the main reason why the metabolic effects promoted by the TuD therapy are way more potent than the effects triggered by our GalNAc-conjugated antimiRs, which, very specifically, inhibit the two target microRNAs without affecting the expression of other components of the cluster. In addition, recent reports have attributed important effects of its own to the host transcript lncMGC (please see reference #51), so it cannot be discounted that the specific inhibition of this transcript may also contribute to the metabolic effects promoted by the TuD. Having said that, we have previously demonstrated that specific inhibition of miR-379 by antisense oligonucleotides is sufficient to affect the regulation of lipoprotein uptake by the liver and reduce circulating triglyceride levels (please see refence #21), indicating that targeting independent microRNAs from this cluster can also result in distinct metabolic effects. Nevertheless, no effects on glucose homeostasis were ever reported in response to such manipulation. Herein, we now demonstrate that combined inhibition of miR-379 and miR-541 a GalNAc-conjugated antimiR is able to promote effects on lipids, by reducing circulating triglycerides as expected, and on glucose homeostasis, by enhancing the action on glucose levels of an exogenous bolus of insulin. Whether the effects on glucose are miR-541 specific or result from the synergism of miR-379 and miR-541 inhibition remains to be clarified, it is also true that our data demonstrate that overexpression of miR-541 is sufficient to promote insulin resistance, an effect not previously reported in response to miR-379 overexpression (reference #21). The effects on glucose metabolism are certainly amplified by the downregulation of several microRNAs from the cluster, as observed in response to the TuD therapy.

10. The authors showed that the inhibition of miR-379/miR-541 enhanced the uptake of lipoproteins in primary hepatocytes (Figure 3). Similarly, a previous report showed that the

miR-379/miR-544 cluster is resistant to high-fat diet-induced steatosis (C. Cao et al., Frontiers in Cell and Developmental Biology 9, 720900 (2021)). Although the authors of this manuscript found no changes in ALT/AST and liver TG in regular chow diet-fed mice, they should evaluate whether miR-379/miR-541 inhibition induces liver inflammation/fibrosis, steatosis (hepatic TG accumulation), and increased plasma ALT/AST in STZ-challenged mice and high-fat/high-sucrose diet-fed mice.

Despite the enhanced lipoprotein uptake by the liver of animals treated with our TuD, we observed no increased in hepatic lipid deposition in animals fed regular chow (new Supplementary Figure 9A and 9B). This might be explained by concomitant elevation in fatty acid oxidation, as indicated by our new data on mitochondrial oxidation (novel Figure 7E and 7F). As per reviewer request, we have now examined hepatic lipid deposition in animals on a high-fat, high-sucrose diet and found that, in this context, hepatic triglyceride accumulation is slightly elevated (results also shown in new Supplementary Figure 9A and 9B); suggesting that the enhanced mitochondrial activity is not sufficient to overcome the increased flux of fatty acids to the liver in this model. According to the higher degree of steatosis found in this model, circulating levels of ALT and AST are also mildly albeit significantly elevated (new Supplementary Figure 18F). Nevertheless, we also have now examined liver inflammation by studying the number of inflammatory foci per 200x field of view in liver slices (please see reference #28). Importantly, we found no obvious signs of inflammation neither in regular chow-fed animals (new Supplementary Figure 7G) nor in DIO animals with hepatic-specific repression of the *miR-379/miR-410* cluster (new Supplementary Figure 18G), supporting the lack of major burden to the liver. Unfortunately, we lack the samples to study the same parameters in STZ-treated mice.

The apparent discrepancy between our novel findings and those reported by Cao C et al in 2021 (cited in our manuscript as reference #20) might be explained by the fact that the KO model studied in the previous report is constitutive and, therefore, affects also the adipose tissue, which

has been demonstrated to be highly sensitive to *miR-379/miR-410* cluster inhibition by notably reducing adipose tissue expansion. Thus, the reduction in liver steatosis reported in this study might very well be secondary to the decreased fatty acid influx coming from the adipose tissue.

11. In Figure 4, the authors proposed increased FGF21 secretion from the liver as a possible mechanism for improving insulin resistance in muscle and adipose tissues. How do miR-379 and miR-541 increase hepatic FGF21 expression? Is it possible that miR-379 and miR-541 directly regulate FGF21 expression? A recent study showed that the inhibition of SLC25A47 in mitochondria led to decreased mitochondrial oxygen consumption, increased mitochondrial stress responses, and increased FGF21 expression in the liver (N. Bresciani et al., Journal of Hepatology 77, 1071-1082 (2022)). The authors should assess the effect of miR-379/miR-541 knockdown on mitochondrial oxygen consumption. The manuscript may be strengthened by including studies evaluating whether FGF21 inhibition (e.g., using antisense oligonucleotides) negates the protective effect of miR-379/miR-541 inhibition on insulin sensitivity.

Following the reviewer's suggestion, we have now assessed mitochondrial oxygen consumption in mitochondrial extracts from livers with repression of the *miR-379/miR-410* cluster. In agreement with our previous observations on enhanced mitochondrial connectivity, expression of the TuD inhibitor was associated with a remarkable elevation in oxygen consumption rate in conditions of either basal respiration, ADP-induced respiration or maximal respiration in response to FCCP incubation (novel Figure 7E and 7F), supporting elevated mitochondrial activity. The results are normalized to mitochondrial protein content, therefore precluding any confounder effects of the enhanced mitochondrial mass (novel Supplementary Figure 16C-E).

On another note, we thank the reviewer for providing the reference of the excellent study by Bresciani and colleagues. However, we do not believe that the same stimuli that promote FGF21 release in the report by Bresciani could apply in our own study. On one hand, the elevation in

FGFG21 levels described in Bresciani's work is remarkably robust, while here in our study we report a 2- to 3-fold increment in FGF21 circulating levels in response to the TuD therapy, a potentiation that falls within the physiological range and it is comparable to the increase in FGF21 levels observed in fasting conditions as compared to the postprandial state. On the other hand, and most importantly, mitochondrial activity is not impaired in our study (as reported by Bresciani) but robustly enhanced, as discussed above. *In silico* analysis performed by us did not predict *Fgf21* to represent a direct target of the microRNAs form the *miR-379/miR-410* cluster, so we hypothesize that the elevation in FGF21 levels reported in our study might be explained by alternative reasons, such enhanced PPAR signaling in the liver of animals carrying the TuD inhibitor. Accordingly, herein we report dramatic upregulation of downstream effectors of PPAR signaling such as *Cd36* and *Lpl* (both at mRNA and protein level). Also supporting this, the newly run RNA-seq analysis shows marked upregulation of other targets of PPAR activation such as *Slc27a1*.

Finally, we have now conducted a novel study to investigate the role of FGF21 in the metabolic effects promoted by the liver-specific repression of the *miR-379/miR-410* cluster. In this study, rAAVs carrying either the TuD inhibitor or a negative control sequence were delivered to Fgf21 KO mice. The results of this novel experiment are shown in novel Figure 5K-M and novel Supplementary Figure 10O-Q as well as described in the results section as follows:

"……..an observation that could be partially explained by the upregulation (Supplementary Fig.10M) and increased circulating levels (Fig.5J) of FGF21, a hepatokine known to modulate glucose uptake by peripheral tissues[30,31]. To test this hypothesis, rAAVs expressing either the TuD inhibitor or a negative control sequence were delivered to Fgf21 KO mice[32]. As anticipated, the 2.5-fold increment in FGF21 circulating levels elicited by the TuD therapy in WT animals was abolished in Fgf21 KO mice (Supplementary Fig.10N). Despite the lowering effects on circulating lipids induced by the TuD were not affected by the lack of FGF21 (Supplementary Fig.10O), the

decrease in fasting glucose, as well as the reduction in glucose excursions following a PTT promoted by the TuD therapy were significantly attenuated in the absence of FGF21 (Fig.5K-L). Moreover, although the lack of FGF21 did not compromise the improvements in glucose clearance induced by the TuD inhibitor (Supplementary Fig.10P), insulin sensitivity was significantly attenuated in the Fgf21KO animals, as demonstrated by reductions in surrogate measurements such as the insulin sensitivity index (ISI; Fig.5M) and the HOMA-IR (Supplementary Fig.10Q). Collectively, our findings demonstrate that the *miR-379/miR-410* cluster plays a major role in the regulation of lipoprotein uptake by the liver, as well as in the regulation of hepatic glucose output and hepatic insulin sensitivity in young adult mice, effects that have an impact in whole-body energy homeostasis. Although not instrumental, the elevation in FGF21 levels partially contributes to the effects on glucose homeostasis."

12. Using western blot analysis, the authors identified the pathway leading to Akt phosphorylation and mTOR activation, presenting it as a liver-specific mechanism. However, it is important to consider that activation of the insulin signaling pathway, including IRS-PI3K, can be viewed as an aspect of improving systemic insulin resistance. As indicators of insulin resistance, activation of downstream pathways, including Akt, is expected not only in the liver but also in the muscle and adipose tissue. Therefore, the authors should provide evidence for this in their study. Consequently, it cannot be conclusively proven here that miRNAs interact directly with Akt and mTOR.

We agree with the reviewer that enhanced PI3K/mTOR signaling can be secondary to the general improvement in insulin sensitivity. As a matter of fact, our data shows that hepatic-specific repression of the *miR-379/miR-410* cluster improves insulin sensitivity, not only locally, in the liver, but also in extrahepatic tissues, such as the skeletal muscle and the brown adipose tissue, as demonstrated by our euglycemic-hyperinsulinemic clamp studies. Whether the improvement in insulin sensitivity in such tissues is mediated by factors released by the liver, in response to the

manipulation of the cluster of microRNAs, remains to be clarified; but new studies included in the revised version of our manuscript and discussed above, point towards a partial role of FGF21 on these effects. Most interestingly, previous reports cited in our manuscript have demonstrated direct interaction of the microRNAs from the *miR-379*/*miR-410* cluster with different components of the PI3K/mTOR signaling pathway and have shown PI3K/mTOR hyperactivation in response to the repression of the cluster in hematopoietic stem cells, suggesting direct regulation of the pathway by the microRNAs (please see reference #26). Accordingly, enriched pathway analysis of the predicted target genes for different microRNAs of the cluster (including *miR-379* and *miR-541*) performed by us (novel Supplemental Figure 11) has identified the PI3K/AKT/mTOR signaling pathways as one of the top target pathways of these microRNAs. Moreover, novel RNA-seq data included now in the manuscript identifies the PI3K/mTOR signaling pathway as one of the top upregulated pathways in the liver of animals with repression of the *miR-379/miR-410* cluster (new Supplemental Figure 12D). Overall, supporting a role for the *miR-379*/*miR-410* cluster in the regulation of PI3K/mTOR signaling also in hepatocytes. In addition, we have now examined PI3K/mTOR signaling activation in both liver and skeletal muscle samples from the same animals under basal conditions. As already shown in the original version of our manuscript, phosphorylation of AKT, FOXO1, ribosomal protein S6 and 4EBP1 was significantly augmented in the liver samples of animals carrying the TuD sequence (new Figure 6 in the revised version of our manuscript). In contrast, no increased phosphorylation of AKT, FOXO1 or 4EBP1 was found in the skeletal muscle samples from the same animals, and, although the phosphorylation of ribosomal protein S6 was mildly enhanced in skeletal muscle (by 1.7-fold), this effect was notably less robust that the one observed in the liver (data included in novel Supplemental Figure 14). Moreover, a critical component of mTOR signaling, which is also a well established target of miRNAs from the miR-379/miR-410 cluster and it is required for the full activation of AKT, RICTOR, was robustly upregulated in response to the repression of the cluster promoted by the TuD therapy in the liver (data shown in new Supplemental Figure 6E and 6F), but was not affected

in the skeletal muscle of the same animals (novel Supplemental Figure 14E). Overall, demonstrating that the enhanced PI3K/mTOR signaling observed in the liver of animals with hepatic-specific repression of the *miR-379*/*miR-410* cluster is likely not secondary to the whole-body improvement in insulin sensitivity, but most likely a consequence of direct regulation of the pathway by the cluster of microRNAs.

13. The authors proposed that decreased amino acid catabolism is a mechanism for reduced gluconeogenesis. Were the glutamine/glutamate and methyl malonyl-CoA levels increase in the experiment shown in Figure 5D? Is it possible that these metabolites do not enter the TCA cycle, followed by gluconeogenesis?

As described in the methods section, sequential methoximation and silylation reactions were performed before the metabolomic analysis, this is known as chemically derivatization and it is done to enhance volatility, gas chromatography resolvability and mass spectrometry ionization. Unfortunately, glutamine and glutamate can cyclize during the derivatization reaction to form pyroglutamic acid which is an artefact. Therefore, the values for glutamate and glutamine obtained by the proteomic analysis are not reliable and their intracellular concentrations were not considered. Moreover, methylmalonyl-coA was, unfortunately, not detected in the analysis, so we cannot hypothesize on its potential role in the metabolic effects described by us.

14. Supplementary Figure 8C shows an increase in GKAP1 expression, which activates insulin signaling. Have the authors also investigated whether miRNAs in the miR-379/miR-410 cluster target GKAP1?

In silico analysis predicted *Gkap1* to represent a target for a number of microRNAs from the *miR-379*/*miR-410* cluster, including *miR-379*, *miR-382*, *miR-134* and *miR-541*, four of the microRNAs which were confirmed to be downregulated in response to the TuD therapy. The complete list of

microRNAs from the cluster predicted to interact with *Gkap1* is shown in novel Supplementary Figure 13F.

Minor points

1. In the Introduction section, the authors express the genomic position using the term “3' end.” However, is it not more common to indicate whether it is on the telomeric or centromeric side?

We agree with the reviewer that using “3’ end” terminology when referring to the relative position of a sequence of genomic (and therefore double-stranded) DNA might be confusing for the readership. We now express the relative position of the *miR-379/miR-410* cluster in the locus as distal (closer to the telomere).

2. The following is described in the Introduction section: “We also describe a novel approach to repress the entire miR-379/miR-410 cluster (the largest congregate of miRNAs in mammals) specifically in hepatocytes, by an AAV-delivered tough decoy (TuD), targeting distinct regions of the primary transcript of the cluster.” Is it correct to describe the suppression of the entire cluster as a completely new approach? If this is completely new, then there is no need to add a reference, of course. However, if a previous report does exist, they should cite the paper and mention the advantages and disadvantages it pointed out.

The reviewer is correct that this is not the first time that repression of the *miR-379/miR-410* cluster is attempted, there are previous reports on animals carrying total or partial deletions of the *miR-379/miR-410* cluster and we have dedicated an extensive part of the introduction section in our

manuscript to present and discuss these studies (please see lines 75-95). We do not pretend to claim that this is the first time that repression of the cluster is achieved. We do claim, however, that we have designed a novel approach to repress this cluster in a liver-specific manner and, therefore, managed to avoid significant confounder effects in other metabolic tissues such as adipose tissue and skeletal muscle, as effects of this cluster on adipogenesis and muscle hypertrophy have been reported. To our knowledge, this statement is true. It is also not the first time that the host transcript *lncMGC* has been targeted, as the group of Kato o Natarajan managed to target this lncRNA in the kidney of mice employing GapmeRs directed against the transcription start site of *lncMGC* (reference #14). Although this work was already cited in the original version of our manuscript, the targeting of *lncMGC* by GapmeRs is now explicitly mentioned when discussing the outcome of the antisense oligonucleotides tested by us. Nevertheless, it must also be noted that, although *lncMGC* targeting was successful in this study, the resulting downregulation of the microRNAs from the cluster was modest as compared to the 90% reduction obtained by our approach in the liver. Accordingly, we feel that talking about frank repression of the cluster when referring to the previous work by Kato and colleagues would be an overstatement.

3. It is wonderful that the authors have evaluated the proteome, but why did they not add RNA-Seq data as additional information on the effects of whole-cluster inhibition? Because miRNAs usually play a role in transcriptional interference, it may be worthwhile to examine their impact on overall gene expression.

Our rationale to conduct proteomic analysis over transcriptomics was that microRNAs can repress translation of the target mRNAs without inducing degradation of these targets. As a result, effects on the protein levels of the targets may not be reflected by parallel changes at the mRNA level. Having said that, we concur with the reviewer that RNA-seq analysis is more sensitive than

proteomics and can detect a much higher number of transcripts, while some of the corresponding proteins might be missed in the proteomic analysis. We have now conducted bulk RNA-seq in liver samples from animals expressing the TuD or a negative control sequence to complement our findings on the proteome. The results of the analysis are shown in novel Supplemental Figure 12. Important findings are the identification of *Lpl* and *Cd36* (predicted to represent direct targets of a large number of microRNAs from our cluster of interest) as some of the top upregulated transcripts in response to TuD therapy, confirming our previous observations by qPCR analysis; the identification by enrichment analysis of the PI3K-AKT signaling pathway as one of the top upregulated pathways in response to TuD therapy, thereby supporting our previous findings on enhanced activity of PI3K/mTOR signaling in the liver; as well as the identification of several pathways predicted to be commonly targeted by numerous microRNAs from the cluster (please see our response to major point # 8 above) as some of the top upregulated pathways in the liver of animals on TuD therapy, including not only PI3K-AKT signaling pathway, but also focal adhesion and regulation of actin cytoskeleton.

4. In genomic analyses of human populations, such as GWAS, does the locus containing the targeted miRNA show strong associations with metabolic disorders, such as diabetes and hyperlipidemia?

We acknowledge that, apart from the link between the cluster and NAFLD, the original version of the manuscript lacked information on the relation between the *miR-379/miR-410* cluster and pathological conditions in humans. We have now corrected that by introducing in the discussion the Temple and Kagami-Ogata syndromes, imprinting disorders of human DLK1-DIO3 locus linked to endocrinopathies; as well as the results of genome-wide analyses revealing enhanced expression of the non-coding RNAs from the cluster in atherosclerotic plaques.

5. Endoplasmic reticulum stress-CHOP has been shown to induce the expression of lnc-MGC (M Kato et al., Nature Communications 7, 12864 (2016)). Did ER stress increase in the authors' model in which miR-379/miR-541 was elevated?

As pointed out by the reviewer, Kato and colleagues demonstrated regulation of *lncMGC* by TGF-β1 and CHOP in mouse mesangial cells. They also showed increased mRNA levels of CHOP in glomeruli of STZ-treated mice. With this precedent, one may argue that the consistent upregulation of the *miR-379/miR-410* cluster that we observed in the different models of hepatic insulin resistance, could be secondary to elevated ER stress in the liver of those animals. However, we have studied the mRNA levels of two canonical markers of ER stress (CHOP and *Atf4*) and found no elevation of such markers, thus precluding the implication of ER stress in the effects described by us (these novel findings are shown in new Supplemental Figure 3E-G).

6. The authors should provide more information in Figures 1 and 2, such as tissues (liver or adipose), treatment duration (4 weeks or three months), and species (humans or mice), for a better understanding of each figure.

We fully agree with the reviewer on the need to clearly identify and separate the experiments completed in humans and mice. Accordingly, new Figure 1 and Supplementary Figure 1 exclusively show data from human samples now, while the other main body and supplemental figures in the manuscript show data from mouse samples.

Also, as suggested by both reviewers, treatment duration has been properly labeled in new Figure 3A and 3B, as well as in new Supplementary Figure 6C and 6D.

Finally, the panels showing absence of effects on *miR-379*, *Mirg* and *miR-541* levels in white adipose tissue in response to the TuD therapy have been now removed and replaced by novel

Figure 3C and 3D, and novel Supplementary Figure 6B; showing absence of effects in levels of expression of these non-coding RNAs in extrahepatic tissues (white and brown adipose tissue, heart, skeletal muscle and hypothalamus) following TuD therapy. Thus, reinforcing the high tissue specificity of our approach.

7. In Figure 2, the authors showed reduced expression of lnc-MGC in the AAV-TuD miR-379/miR-541 group. Are there any previous reports on lnc-MGC knockout mice?

As already discussed above, there are several previous reports on animals carrying total or partial deletions of the *miR-379/miR-410* cluster and we have dedicated an extensive part of the introduction section in our manuscript to present and discuss these studies (please see lines 75-95). There is also a relatively novel study from the group of Natarajan and Kato presenting yet another KO model related to this cluster of microRNAs (please see refence #51). In this report, the authors generate a *lncMGC* KO model by introducing a deletion at the transcription start site of this lncRNA. As a result, the expression of l*ncMGC* is blunted. Remarkably, expression of other components of the cluster such as *miR-379* and *Mirg* was not severely reduced, suggesting that *lncMGC* may not be the only primary transcript from where the microRNAs of the cluster derive. Interestingly though, in this study the authors describe specific effects of *lncMGC* which are independent from the effects of the miRNAs. More specifically, the authors demonstrate interaction of *lncMGC* with a number of proteins, including nucleosome remodelling factors, ultimately enabling the regulation of gene expression by *lncMGG* through the induction of chromatin relaxation. These findings support our hypothesis that targeting the host transcripts of miRNAs from the *miR-379/miR-410* cluster (*lncMGC* and *Mirg*), promotes more robust metabolic effects than using specific antisense oligonucleotides against individual microRNAs. Not only because several microRNAs derived from these lncRNAs are affected, but also because the primary transcripts themselves can play important biological roles. Accordingly, our results are now discussed in the light of these new findings.

In a more recent study, published while we were conducting the revision of our manuscript, the same group examined the effects of STZ-induced diabetes in their *lncMGC* KO model (please see reference #54). As previously reported for other constitutive KO models of the *miR-379/miR-410* cluster, the authors describe effects on glucose homeostasis, namely their *lncMGC* KO mice are resistant to the development of STZ-induced diabetes. As discussed above, expression of *Mirg* and miRNAs from the cluster is not severely reduced in this model, which might explain why the phenotype is not as dramatic as the one of previous KO models and the animals can survive up to adulthood. The authors identify the pancreatic islets as the primary beneficiaries of *lncMGC* knockdown, as the *lncMGC* KO mice were able to preserve the structure of pancreatic islets and their ability to secrete insulin in response to the STZ treatment. In contrast, we report no effects on pancreatic islets in our STZ-treated mice expressing the hepatic-specific TuD, as demonstrated by comparable circulating levels of insulin between STZ-treated animals receiving the AAV-NC and the AAV-TuD therapy (as shown in new Supplementary Figure 17C). Moreover, we have now included novel data on histological analysis of the pancreas of our STZ-treated animals, showing no effects of the TuD therapy on α-cells and β-cells population, neither on the alpha-to-beta cell ratio; supporting the liver-specific effects of our manipulation (new Supplementary Figure 17B).

8. It is possible that I may be misinterpreting the data, but I do not believe it can be conclusively stated from Figure 2B that the effect of rAAV lasted for 3 months. The figure does not appear to show a temporal alignment with the improvement in triglyceride levels for up to 5 weeks. If the authors want to demonstrate long-lasting effects, they should provide detailed evidence of the improvements in these phenotypes. Otherwise, there may be no need for a long-term focus in this paper.

With respect, when we claim that the effect of the TuD therapy lasted for 3 months, we mean that the repression of the *miR-379/miR-410* was still obvious and very remarkable 3 months after rAAV administration, as it can be appreciated in new Figure 3B (former Figure 2B). It must be noted that our in vivo experiments are regularly 5 weeks long and that is why most of the metabolic data that we show range within this time window. Some of the novel studies that we run for this revision were extended for 2 extra weeks for a total of 7 weeks. At this time point, significant effects of the TuD therapy on fasting glucose levels were still present (please see new Fig.5B). Moreover, in the original long-term (3 months) study that we ran, we observed reduced glucose excursions following a pyruvate tolerance test (new Figure 5D) as late as 3 months after rAAV administration in the animals with hepatic-specific repression of the *miR-379/miR-410* cluster. Overall, supporting a long-lasting effect of the TuD therapy on hepatic glucose output.

9. In Figure 4I-J, TuD increased glucose uptake in the skeletal muscle and BAT. The increase in Fgf21 expression was notable but not very large. Please add additional discussions on the proteome or RNA-Seq data from the TuD-treated/control liver tissue (if any) and the decrease in adipose tissue mass (if any).

As already pointed out in our response to major comment #11, we recognize that the effect on FGF21 circulating levels reported in our study is not comparable to the robust response described in the study authored by Bresciani et al. However, our effect on FGF21 levels is very persistent, as it is maintained for, at least, 3 months after rAAV administration, and physiologically meaningful, as the 2- to 3-fold increment in the circulating levels of FGF21 reported by us is comparable to the increase in FGF21 levels in fasting conditions as compared to the postprandial state. Supporting this, our novel study in FGF21KO mice demonstrates that preventing the FGF21 response promoted by the silencing of the cluster in the liver, attenuates some of the effects on glucose metabolism.

Following the reviewer's suggestion, we have now discussed more extensively the data revealed by the proteomic and the novel RNA-seq analysis. Thus, we have maintained our discussion of the inhibition of the amino acid metabolism pathways and their potential role on the supply of substrates for gluconeogenesis, as well as the role of GKAP1 as an activator of insulin signaling. We have now also discussed the findings on protein levels of some critical regulators of mitochondrial pyruvate metabolism, indicating that oxidation of pyruvate is favored over its use as gluconeogenic substrate. Regarding our RNA-seq findings, we have now extensively discussed the role of miRNAs from the cluster as regulators of PI3K-mTOR signaling.

Finally, we have now added a sentence in the discussion dealing with the reduced adipose tissue depots found in obese animals, as follows:

"…..although energy expenditure was not altered in mice fed regular chow, a potential effect in obese mice, which would explain the decrease adipose tissue mass found in these animals, remains to be clarified".

10. In these figures, the authors used both AAV-TuD and AAV-TuD 541/379 descriptions. Are there differences between the two? If not, the authors should standardize the descriptions to avoid confusion.

Both AAV-TuD and AAV-TuD 541/379 refer to the same treatment, we apologize for the confusion. We have now amended that inconsistency and utilized the same terminology in all the legends.

11. The authors should include a dot plot in the figures.

We concur, and we have now represented all the individual values in the histograms.

Reviewer #3 (Remarks to the Author):

We thank the reviewer for the insightful comments.

Summary:

In "Combinatorial targeting of the miR-379/miR-410 cluster normalizes glucose and lipid homeostasis in diabetes and obesity," Gil-Lozano and colleagues demonstrate that microRNAs from the miR-379/miR-410 cluster, known to be important in pre-natal development, are also important for post-natal liver metabolism. Specifically, the authors identify upregulation of miR-379 and miR-541 in humans with obese BMIs. The authors design and test both an LNA-based antisense oligonucleotide and TuD inhibitor targeting miR-379 and miR-541 to elucidate the biological impacts on hepatic miR-379/miR-410 and subsequent metabolic endpoints, finding that the reduction of these microRNAs improved glucose homeostasis. Ultimately, the authors test the functional consequences of the TuD in vivo, targeting hepatic tissue in STZ and diet-induced obese male mice and presenting impressive data in Figure 7 that suggests the miR-541 and miR-379 TuDs ability to counteract metabolic hallmarks in mouse models of both Type 1 diabetes and diet-induced obesity. Collectively, the data presented demonstrate a novel decoy mediated approach to alter common metabolic endpoints associated with type 1 diabetes and diet-induced obesity.

While the data are intriguing, there are several aspects of this study that would benefit from further investigation and/or clarification, as described below:

Major Points:

- While single cell sequencing seems beyond the scope of the studies, it is unclear from the results whether the increase in microRNAs arise from an increase in expression in

one cell type (e.g. hepatocytes) or increased relative abundance of other cell types in the liver. Is tissue available for IHC studies?

We have now run liver fractionation studies and observed that the basal expression of microRNAs from the *miR-379/miR-410* cluster is comparable between hepatocytes and non-parenchymal cells. Most importantly, we also observed that treatment with glucocorticoids only increases the expression of this cluster in the hepatocyte fraction, while the non-parenchymal cell fraction is insensitive to the stimulation (data shown in novel Supplementary Figure 2E-G) This demonstrates that, at least in the models of glucocorticoid exposure, the upregulation of the microRNAs from our cluster of interest is hepatocyte driven. Supporting this, we also reported upregulation of different components of the cluster in primary hepatocyte cultures incubated with dexamethasone (data shown in new Supplementary Figure 6C-D). Unfortunately, we have no data to support that the upregulation of microRNAs from this cluster in human samples or other experimental models, such as STZ-treated mice, is also hepatocyte-specific.

- The amount of total RNA for microRNA studies, 10 ng, is a very low amount, and the microRNAs in question typically have very low expression in the adult liver. Since everything is normalized to a control value of “1”, it is hard to know the absolute amount of these microRNAs and whether the fold-change is meaningful, especially given the low number of human samples that were evaluated. Validation through another mean (e.g. northern blot) would be preferable.

The reviewer is right that the input of total RNA for our microRNA gene assays was 10 ng. According to the manufacturer’s instructions, the Taqman MicroRNA Reverse Transcription Kit, which is the kit we used for the reverse transcription of the miRNAs in our samples, requires between 1 and 10 ng of total RNA as starting material; so we are positive that we amount of RNA we used was adequate. It is also true that the levels of expression of our miRNAs of interest in

adult mice are relatively low as compared to newborn animals. This can now be appreciated in novel Supplementary Figure 3D, which is now included in the manuscript as per request of reviewer #1. Nevertheless, whether decrease in miRNA expression is also a characteristic of adult human livers remains to be clarified.

As pointed out by the reviewer, in former Figure 1A and 1B, the relative levels of expression of the microRNAs were normalized to the levels of expression of the control group (BMI<30) to facilitate the visualization of the difference between the two groups being compared. However, in the new version of the manuscript we now represent the expression of the microRNAs relative to the internal control, without further normalization, to facilitate the comparison between the two microRNAs in question.

We respectfully disagree with the reviewer's take that quantification with northern blot would be preferable. Although we do not question the validity of northern blot, we firmly believe that the far superior sensitivity of qRT-PCR makes this technique a better option for the quantification of miRNA expression levels, particularly when studying transcripts with low copy numbers. We would like to emphasize that Taqman technology was used for the quantification of miRNAs in our study. This technology involves the use of specific probes for the target sequence (similarly to northern blot) and it is characterized by far superior sensitivity and specificity as compared to alternative qRT-PCR technologies. In any case, the human samples were no longer available to us, so it was not possible to run northern blots.

- The data reporting and experimental design considerations are not in alignment with the Nature Portfolio guidelines on sex. For the human data in Supplementary Table 1, the data regarding the sex of human samples is omitted. This information should be included, as sex as a biological factor is important, especially in the obesity and diabetes literature.

Furthermore, only male mice were used in the in vivo portion of the work. While the findings presented are impressive, the investigation of sex as a biological factor in these experiments is important prior to publication, or the title and resulting manuscript should be revised to clearly reflect that this work was only completed in male mice, as the current language in the manuscript is indicative of the data being applicable to both males and females, and thus, very misleading.

We completely agree with the reviewer that sex is an important biological factor that is often overlooked. The information regarding the sex of the donors of the human sample is now included in Supplemental Table 1. As is often the case for studies involving human volunteers, the majority of the donors are females, who constitute approximately 80% of the volunteers. Given the discrepancy in sex between our human data and our *in vivo* experiments with mice, and considering the relevance of sex for the regulation of glucose and lipid homeostasis, we decided to design and perform a novel *in vivo* study comparing the effects of TuD therapy in male and female mice. The most important results from that study are now shown in the new figures 4 and 5. Thus, new figures 4C and 4D show, respectively, the effect of the TuD in the circulating levels of triglycerides, and total cholesterol and LDL cholesterol concentrations in both males and females. The new panels in figure 5 compare the effects of the TuD in fasting glucose (Fig.5B), as well as the glucose profile in response to a glucose (Fig.5E), insulin (Fig.5F), as well as pyruvate (Supplementary Figure 10E) tolerance tests, between males and females. These new panels replace former ones showing effects exclusively in male mice; in addition, the methods, results and discussion sections were modified accordingly in the context of these new findings. Overall, the new data confirm the sharp metabolic differences between male and female mice, with female mice showing reduced circulating levels of triglycerides, LDL cholesterol and fasting glycaemia, as well as improved glucose clearance and reduced glucose excursions in response to pyruvate and insulin tolerance test as compared to males. Most importantly, it also shows that

the metabolic changes induced by the TuD therapy, previously reported exclusively in males, are also observed in female mice, indicating that the effects of suppressing the *miR-379/miR-410* cluster in the liver are not sex-dependent. Irrespective of these novel findings, since all of the animals we used as models of diabetes and prediabetic diet-induced obesity were exclusively males, we decided to change the title of our manuscript to:

"Combinatorial targeting of the *miR-379/miR-410* cluster normalizes glucose and lipid homeostasis in male models of diabetes and obesity".

- Line 152: A 6h fast is mentioned, as well as "increased basal glycemia in morning fed animals." Does morning refer to lights on? Throughout the manuscript is it not abundantly clear what time of day the mice were fasted, expect for the data illustrated in Figure 3A. As rodents are nocturnal and consume most of their calories during the night (and have the greatest physical activity), this information is vital and must be explicitly stated in the methods for each experiment. Additionally, it is not clear if chewing and/or running enrichments were present in the home cage environment.

We agree with the reviewer that a detailed description of the timing of our studies was missing in the original version of the manuscript. We have now rectified that by adding the following statement in the methods section:

"As a general rule, samples were collected at ZT2 as a representation of a postprandial state, while food was removed at ZT3 and then samples collected at ZT9, as a representation of a fasting state."

In addition, we have now also specified that metabolic tests were "consistently run at ZT9".

Regarding environmental enrichment, as now stated in the methods section: “Cages were provided with bedding and nesting material, hiding structures and chewing implements to enhance environmental enrichment”. No spinning wheels were provided.

- Line 214: The authors claim no obvious toxic effects were observed, but only body weight and water intake data were assessed. A more thorough toxicology panel is needed to support this claim.

As shown in former Supplemental Figure 5 (current Supplemental Figure 7), no significant changes in body weight, water and also **food intake** were observed between the animals receiving the TuD therapy and the control group over a 3-month period. Furthermore, it also shows comparable circulating levels of various bona fide markers of liver and kidney damage, thus indicating that no major harm was induced in these tissues. To complement these findings, we have now included in new Supplemental Figure 8 the hourly profile of food and locomotor activity over a 4-day period. Again, showing no major differences between the two groups. As one could argue that obvious toxic effects in the liver would likely be reflected in changes in the animal behaviour (mostly food intake and activity) or in the circulating levels of liver enzymes, we are confident on our claim that no obvious toxic effects were observed. Moreover, we also studied now liver inflammation by counting the number of inflammatory foci per 200x field of view (please see reference #28), and no signs of obvious inflammation were found neither in the animals carrying the TuD sequence nor in the negative control animals (results shown in novel Supplemental Figure 7G). We hope the reviewer will find our new data sufficiently convincing to align with our claim that no obvious toxic effects were observed in our mice fed regular chow. We acknowledge, however, that, although no evident signs of inflammation were detected in the HFHS-fed animals (results shown in novel Supplemental Figure 18G), steatosis seemed to be slightly, albeit significantly, elevated in the liver of diet-induced obese animals treated with the TuD

(as shown in novel Supplementary Figure 9A). According to the mild lipid accumulation, circulating levels of ALT and AST were also slightly augmented (as shown in novel Supplementary Figure 18F) and thus, it cannot be discounted that the steatotic phenotype of DIO obese animals could worsen in the long-term in animals with hepatic-specific suppression of the *miR-379/miR-410 cluster.*

- Figure 6B and C: Is there a way to label hepatocytes in these samples? The data from Figure 6A is hepatocyte specific, while these figures examine general liver tissue. Alternatively, would it be possible to complete these stains in cultured hepatocytes for consistency?

Although it is true that the staining shown in former Figure 6A (current Figure 7A) was performed on hepatocytes in culture, while the staining shown in former Figures 6B and 6C (current Figures 7B and 7C) was run on liver tissue slices; it must be noted that the quantification was performed by a pathologist with extensive experience in the identification of hepatocytes. Moreover, although former Figure 6B (current Figure 7B) shows images with relatively low magnification that were selected for publication to better display the striking difference between the two treatments being compared; images with higher magnification, which allow the distinctive identification of the different cell types, were used for the quantification. In essence, we can ensure that only cells with distinctive hepatocyte morphology were considered in the analysis; we clarify this now in the methods section as follows:

“Only cells distinctively identified as hepatocytes were considered in the analysis”.

Finally, we recognize that the identification of the cells presented in the images of former Figure 6C (current Figure 7C) as hepatocytes is more challenging. Hoping to facilitate their identification,

we have now added an extra image by merging the phalloidin and Tom20 staining. In this new merged image, the characteristic polygonal shape of hepatocytes can be more easily appreciated.

- It is not clear why diabetic patients (Type 1 and 2) are not included in the experiments used to generate Figure 1. In addition to a DIO group that receives treatment after the phenotypic change in the final in vivo experiments, this information would better inform the STZ data presented in Figure 7 (ie are miR-379 and miR-541 dysregulated in Type 1 and II diabetes, and if so, what are the implications for this in a mouse model?) This data would significantly strengthen the pre-clinical TuD experiments presented in Figure 7.

We had access to a limited number of liver biopsies (4 females and 2 males) from patients with overt type 2 diabetes and comparable age range to our healthy volunteers and non-diabetic obese subjects. The levels of expression of our microRNAs of interest were also determined in these samples, obtaining a profile of expression very similar to the one found in the obese non-diabetic subjects. Thus, the levels of expression of miR-379 relative to RNU58B were 0.94±0.04, 1.11±0.02, and 1.14±0.02; in healthy, non-diabetic obese, and type 2 diabetic subjects, respectively ($P<0.001$ in control vs. non-diabetic obese and control vs. diabetic, as per one-way ANOVA). In addition, the levels of expression of miR-541 relative to RNU58B were 0.53±0.22, 1.31±0.02, and 1.30±0.03; in healthy, non-diabetic obese and type 2 diabetic subjects, respectively ($P<0.001$ in control vs. non-diabetic obese and control vs. diabetic, as per one-way ANOVA). For obvious ethical reasons, the patients with type 2 diabetes were taking antidiabetic medications (metformin and GLP1-derivative drugs) as well as statins. Because our goal was to identify microRNAs implicated in the regulation of insulin signaling and glucose homeostasis, we intended to find microRNAs whose levels of expression could be associated with indicators of impaired glycemic control and elevated insulin resistance, like HOMA-IR and triglyceride levels. We decided to leave the group of diabetic subjects out of the study because of the confounder

effects that the drugs they were taking would have on these metabolic parameters, this has now been clarified in the methods. Moreover, it cannot be discounted that the medications per se could also affect the normal pattern of expression of the microRNAs. However, for an indication on how diabetes could impact the profile of expression of our microRNAs of interest, the reviewer can refer to new Figure 2F, which shows the expression of *miR-379*, *miR-541, miR-409, lncMGC* and *Mirg* in the liver of STZ-treated animals as compared to control.

- MEG3 is implicated in obesity and Type 2 diabetes. Therefore, the authors should verify that the TuD does not target MEG3 expression as completed in the LNA-based antisense oligonucleotide experiments (Supplemental Figure 2G).

With respect, former Supplemental Figure 2G (current Supplemental Figure 4I) shows the effects of LNA-based antisense oligonucleotide experiments on *lncMGC* levels, not on *Meg3* levels. *LncMGC* is a lncRNA from the mouse genome proposed by certain authors (please see reference #14) as the host transcript of the different miRNAs from the *miR-379/miR-410* cluster. We have certainly evaluated the effects of the TuD therapy on the levels of expression of *lncMGC*, as shown in current Figure 3A and 3B. As a matter of fact, *lncMGC* is severely downregulated in response to the TuD therapy, while it remains unaffected by the treatment with the antisense oligonucleotides. This observation is what lead us to hypothesize that the TuD might not only be targeting the mature forms of *miR-379* and *miR-541* but also their corresponding sequences within the hosts transcripts. This would explain why the TuD therapy induces downregulation not only of *miR-379* and *miR-541* (similarly to the antisense oligonucleotides) but also of the host transcripts lncMGC and *Mirg,* as well as of other representative microRNAs from the same cluster hosted by these lncRNAs, such as *miR-382*, *miR-134* and *miR-409* (as shown in current Figure 3A and 3B), as opposed to the antisense oligonucleotides. On the other hand, the *miR-379/miR-410* cluster is located more than 150 kilobases downstream the *Meg3* sequence, and we have

now collected data showing that the *miR-379/miR-410* cluster and *Meg3* are independently regulated in the liver (Figure 2B and 2D in the revised version of the manuscript).

- The authors should include individual values in the bar graphs to show within group variability throughout the paper. This data presentation is necessary to inform the conclusions drawn.

We concur, and we have now represented all the individual values in the histograms.

Minor Points:

- Lines 58-61 could use additional clarity. Specifically, this section would benefit from the explicit mention of the miRNAs known to be important for insulin signaling in the liver.

As requested by the reviewer, the miRNAs in question have now been explicitly mentioned. We have modified the sentence as follows:

"Another milestone was the identification of different miRNAs involved in the regulation of insulin signaling in the liver, including miR-103, miR-107, miR-802 and miR-143". [6-8]

- Line 65: It would be better to refer to this cluster as being expressed in eutherian mammals (i.e. with a placenta).

We concur and have now added that extra piece of information.

- Line 72: Please refer to the official HUGO gene nomenclature name MEG8 rather than lncMGC.

We are aware that certain databases (including NCBI and HGNC, as the reviewer pointed out) present lncMGC as an alternative name to MEG8, however, we believe that information is misleading. MEG8 is a lncRNA in the human genome which would be equivalent to *Rian* in the mouse genome. Although it indeed hosts the sequence of a small number of microRNAs from the *miR-379/miR-410* cluster, the majority of microRNAs from this cluster in the human genome are hosted in the sequence of MEG9 (another lncRNA). LncMGC is a different lncRNA (a third one) proposed by certain authors (please see reference #14) as an alternative transcript hosting the entire set of microRNAs from the *miR-379/miR-410* cluster. Although in theory, this lncRNA would partially overlap with the distal end of MEG8 in the human genome, it does not perfectly match the sequence of MEG8 as they are two independent transcripts. As a matter of fact, MEG8 also hosts the microRNAs from the *miR-127/miR-136* cluster in humans, which are located several kb upstream from the proposed transcription starting site for lncMGC, making MEG8 a much longer transcript than lncMGC. Furthermore, lncMGC also heavily overlaps with MEG9 in the human genome. Thus, although the existence of lncMGC in the mouse genome seems likely and we were indeed able to determine lncMGC levels in our mouse samples, the existence of lncMGC in the human genome is more controversial at this point, as it is not entirely possible to decide whether the few studies that have examined lncMGC expression levels in humans were actually measuring the levels of expression of MEG8 and/or MEG9.

- Line 90: Please define VLDL in its first utilization.

VLDL is now defined in the text.

- Line 113: The human nomenclature with all-capital letters (DLK1-DIO3) would be preferable here since the locus is being studied in human samples

We agree and have now changed the writing accordingly when we refer to the human locus.

- Line 117: Please define HOMA in its first utilization.

HOMA has now been defined in the text. We also specified now throughout the text that with HOMA we mean HOMA-IR.

- Line 120: Please stay consistent- use either percentage or fold change to describe differences in miR-379 and miR-541 levels.

As a rule, we use % to describe increments lower than 100% and fold change for elevations of 100% or more. However, acknowledging that this can be confusing, we now express all the increments as fold change difference.

- Supplemental Figure 1 E & G: It is not clear why scWAT and skeletal muscle tissue samples were not collected for the corticosterone experiments, but were collected in the dexamethasone experiments to measure relative abundance of miR-379 and miR-541 levels. These tissue types should be presented in the corticosterone experiments. Additionally, the figure legend should clearly specify the experiments completed in mice versus humans.

As indicated in the methods section, this experiment was conducted by our colleagues from the University of Toronto. Unfortunately, they did not collect either subcutaneous white adipose tissue or skeletal muscle samples. As a matter of fact, only liver, epididymal fat and brown adipose tissue samples are available and they all have been already studied here. In any case, we believe that the data from those three tissues is sufficient to support our point that the upregulation of the *miR-379/miR-410* cluster promoted by long-term glucocorticoid therapy in the liver is not a common feature across metabolic tissues, as it was not observed in the epididymal fat or in brown adipose tissue.

On another note, we fully agree with the reviewer on the need to clearly identify and separate the experiments completed in humans and mice. Accordingly, new Figure 1 and Supplementary

Figure 1 exclusively show data from human samples now, while the other main body and supplemental figures in the manuscript show data from mouse samples.

- Supplemental Figure 1 D-G: Tissue abbreviations should be defined in the figure legend.

The abbreviations for the different tissues are now defined in the figure legend as follows:

eWAT=epididymal adipose tissue; **scWAT**=subcutaneous (inguinal) adipose tissue; **BAT**=brown adipose tissue; **SM**=skeletal muscle.

Please note that former Supplemental Figure 1D-G panels are current Supplemental Figure 2A-D panels.

- Lines 124-136: The authors claim strong association between miR-541 and corresponding insulin, cortisol, and leptin levels. However, there is no linear regression presented to back this claim.

We appreciate the reviewer's suggestion regarding the use of linear regression to assess the associations. In the case of *miR-379*, linear correlations indeed provided a reasonable fit to the observed pairwise associations; for example, with plasma concentrations of triglycerides. However, for *miR-541*, the relationships with other variables did not appear to be well described by linear models. Accordingly, in the original version of the manuscript, we preferred to remain cautious and not to apply linear correlation analysis in such pairwise associations. However, we still claimed there was strong association between those parameters because that was our general impression after close examination of the data. In the revised version of the manuscript, Pearson correlations have been replaced by distance correlation analysis for all the pairwise associations. In essence, the distance correlation test detects both linear and non-linear

dependencies, and thus, fits better our data. A more detailed description of the analysis can be found in the statistical section of the methods. The distance correlation (r_d) and the corresponding p-value for each pairwise association can be found in the legend of the corresponding graph. For instance, the distance correlation between *miR-541* levels and triglycerides is 0.76 (P=0.001), while the distance correlation between *miR-541* and HOMA-IR values is 0.61 (P=0.017). Regarding the pairwise associations between *miR-541* levels of expression and the circulating concentrations of hormones, we found distance correlations of 0.64 between *miR-541* and insulin levels (P=0.013) and of 0.78 between *miR-541* and leptin levels (P=0.002). Since most correlations are not linear, the functions are not represented in the graphs. Most interestingly, distance correlation is not restricted to analyzing pairs of single variables; it can also capture joint associations between groups of variables. As an example, the joint association of *miRNA-379* and *miRNA-541* with triglycerides and HOMA-IR yields a distance correlation (r_d) of 0.687 (P=0.008). Overall, this supports our claims of a strong association between the levels of expression of the microRNAs and the metabolic parameters considered.

- Line 144-45: Please explicitly state the name of the liver-specific promoter and which rAAV vector capsid is used.

Although that information was already contained in the methods section, it is now also mentioned when the rAAVs are first introduced for clarity. The text now reads as follows:

"To this end, the pre-miRNA sequence was cloned and delivered into the animals within a recombinant AAV (rAAV) vector (serotype 8). Tissue specificity was ensured with the use of a highly liver-specific promoter known as LP1 promoter[23] (detailed in the methods section)."

- Lines 144-145: When describing the viral construct, the specific AAV serotype and

promoter should be mentioned here. The information is present in the methods, but should be made clear when initially described.

Please see our response to the previous comment.

- Supplemental Figure 2B: It is not clear why Mirg expression levels were not evaluated in the epidydimal while adipose tissue, as this data is meant to reinforce the findings in Supplemental Figure 2A.

As requested by the reviewer, the levels of expression of *Mirg* have now been determined and represented in new Supplemental Figure 4B. As expected, no differences in *Mirg* expression were detected in the epididymal adipose tissue of mice overexpressing *miR-541* in the liver, as compared to control.

- Lines 164-165: As the dose response data for lncMGC is shown despite no effect, it would be advantageous for the authors to also include the dose response data for miR-379 and miR-541 levels.

We felt it was important to show that, despite scaling up the dose, the antisense oligonucleotides did not affect the levels of expression of the host transcript. On the contrary, since the most effective downregulation of the target microRNAs was observed with the 1 mg/kg dose and, accordingly, that was dose tested to evaluate the metabolic effects, we assumed it would be sufficient to show the effects of this particular dose on the levels of expression of the targets. Nevertheless, following the reviewer's suggestion, we have now included the dose-response effect of the antisense oligonucleotides on *miR-379* (new Supplemental Figure 4F) and *miR-541* (new Supplemental Figure 4G) levels, so now the readers can appreciate that increasing the dose of the antisense oligonucleotides does not result in a stronger downregulation of the targets.

- Line 163: "…without affecting other non-coding RNAs from the cluster." The data presented only includes Mirg, to make this statement the other miRNA transcripts should be presented.

Together with the levels of expression of *Mirg,* we have now studied the levels of expression of *miR-382*, *miR-134* and *miR-409* as three representative microRNAs from the *miR-379/miR-410* cluster that are downregulated in response to the TuD therapy but (as shown in new Supplemental Figure 4H) are not affected by the LNA-based antisense oligonucleotides.

- Supplemental Figure 2G: Ascending values would be more useful here- 1 mg/kg looks like there might be a trend toward an effect, or great variability. Individual values should be graphed to address this point.

As per reviewer request, we have now represented the three different doses tested in ascending values (new Supplemental Figure 4 I). Individual dots for every single histogram are also now represented in the revised version of the manuscript, as requested by both reviewers. When comparing the levels of expression of *lncMGC* between the negative control group and the group treated with the antisense oligonucleotides at the 1 mg/kg dose, the Student's t-test yields a p-value of 0.4, precluding any statistical trend towards an effect.

- Line 180: Clarify that the TuD tests in vitro were completed using plasmid constructs, and that the TuD was packaged in an AAV8 for in vivo work later described.

Although the use of plasmid constructs for our *in vitro* studies and the use of rAAVs as delivery vehicles for the *in vivo* studies was already discussed in the methods section, as per reviewer's

request we have now included that information also when the studies are presented for the first time in the results section. Thus, we specify now that:

"The activity of the TuD was first tested *in vitro*, in Hek293A cells transfected with a TuD-coding plasmid under control of the cytomegalovirus promoter".

While following that sentence, we express that:

"The TuD was then delivered into mice within a rAAV8 under the control of a highly liver-specific promoter (LP1)."

- General: italics for in vitro and in vivo.

Agreed, and modified accordingly.

- The sentences on lines 189-191 and 209-213 are confusing. Consider editing for clarity.

We apologize for the confusion; we have now rephrased both sentences with the hope to improve their readability.

"Major advantages of this approach include the avoidance of repeated injections and the toxicity of the nucleotide modifications present in ASOS[25], improved tissue specificity (Supplementary Fig.6A), and increased availability of the inhibitor due to sustained endogenous production. Moreover, given rAAVs vectors are expressed in the nucleus, the potential interaction of the TuD with host transcripts might affect the processing of other miRNAs from the cluster with related functions, yielding a more robust phenotype."

"Importantly, the expression of *Dlk1*, was upregulated in the liver but not in epididymal fat and skeletal muscle following miRNA repression (Supplementary Fig.6J), supporting tissue-specificity of the effects."

- Line 192: This sentence is misleading, as many factors could have contributed to lack of lncRNA knockdown including bioavailability and target binding engagement, not just nuclear localization.

We completely agree with the reviewer that the increased availability of the TuD inhibitor given its sustained endogenous production may have contributed to the more robust metabolic effects driven by the TuD; accordingly, we have now emphasized this in the text as shown above. Moreover, other factors, as the improved ability of TuDs to promote degradation of their target sequences as compared to ASOS could have also played a role, and this has also now been highlighted in the discussion. However, it is also true that ASOS have been proven to not affect the nuclear precursors of miRNAs in hepatocytes, and the fact that they fail to localize in the nuclei of hepatocytes when administered *in vivo* probably offers an explanation. We have now included this information in the discussion, with appropriate references, as it may not be known to a broader audience. In contrast, the sustained intracellular levels of the TuD inhibitors tend to accumulate in the nucleus, which likely favor their interaction with the nuclear precursors of miRNAs, like *lncMGC* and *Mirg* in our particular case. Overall, although we have modified the sentence in question, we opted to maintain our claim that the nuclear localization of the TuD is, more than likely, playing a role on its ability to interact with the corresponding sequences of *miR-379* and *miR-541* within the host transcripts.

- Line 208: Protein was examined for RICTOR and mRNA examined for Atf3. It would be advantageous to also include protein data for ATF3 and transcript expression data for Rictor for consistency.

It must be noted that microRNAs can act by repressing translation of the target mRNAs without inducing degradation of these targets. For this reason, we prefer to show protein levels to

demonstrate derepression of the targets and that is why proteomics were preferred over transcriptomics in the original version of our manuscript. Having said that, our lab has no previous experience with the determination of ATF3 protein levels and, to this point, we lack a reliable antibody for this. We have tested some commercially available antibodies without obtaining clear and reliable immunoblots for any of them. Furthermore, even though we detected up to 10-fold upregulation of *Atf3* mRNA levels in response to repression of the *miR-379/miR-410* cluster, none of the antibodies tested seemed to show any kind of change in ATF3 protein levels, thus reinforcing our lack of confidence in those antibodies. Nevertheless, because the original study demonstrating an effect of microRNAs from the *miR-379/miR-410* cluster in the regulation of ATF3 (please see reference #14) did show mRNA levels and no protein levels, we elected to also show effects on *Atf3* mRNA levels in our study.

- Supplemental Figure 4 H and I: Ladders with molecular weights should be shown on the western blot images.

We concur and have now added the molecular weight marks to all the immunoblot images throughout the manuscript.

- Figure 2A and 2B should be labeled as 4 weeks and 3 months, respectively.

As suggested by the reviewer, new Figure 3A (former 2A) has now been labeled as 4 weeks and new Figure 3 B (former 2B) as 3 months.

- Line 216: Supplementary Figure 5B, no change in body mass observed over 13 weeks, however, it looks like there is a start of a divergence (TuD mice weight less). Would be interesting to see this with HFD.

Respectfully, former Supplementary Figure 5B (current Supplementary Figure 7B) shows data on cumulative food intake and not body weight, which is shown in Supplementary Figure 7A. It can be appreciated that body weight remains comparable throughout the 13 weeks of the study. However, following the reviewer's recommendation, we have now presented the data on body weight from the diet-induced obesity study in new Supplementary Figure 18D. As now shown, no changes in body weight were detected for the chow-fed animals in response to the TuD therapy, consistent with the data shown in Supplementary Figure 7A. However, DIO animals receiving the TuD therapy displayed significantly reduced body weight as compared to NC, in agreement with the reduced adipose depot mass observed in the same group (Supplementary Figure 18B).

- Line 222: The Phenomaster system data should be shown, even if no significant changes were observed. Were the mice single housed in this system? The methods mention that the mice were group housed in home cages, however, there are known behavioral changes when mice are single housed versus group housed.

As requested by the reviewer, the data from the Phenomaster system have now been included in new Supplementary Figure 8. As noted in the new figure, no differences in either food intake or locomotor activity were detected between the two groups, thus supporting our claim of the lack of obvious toxic effects induced by the TuD therapy. In addition, oxygen consumption, carbon dioxide production, energy expenditure and respiratory exchange ratio were also comparable between groups, as previously stated. A short description of the experiment is also provided now in the methods section; here we specify that the animals were indeed individually housed in the chambers of the Phenomaster system. Whether we have obtained a different outcome if the animals had been housed in groups remains to be determined, but group housing would have complicated the interpretation of the data immensely.

- Supplementary Figure 6 C and D: could use additional labels of 5 weeks and 3 months

We agree and have now added the labels accordingly. Please note that former Supplementary Figure 6 C and D is now new Supplementary Figure 9 C and D.

- Line 247: Does 6h fasting reduce blood glucose concentrations at the 3 month mark? This would be a better comparison for the data mentioned in Figure 4D, which examines pyruvate tolerance up to 3 months. Additionally, the text states that "The decrease in blood glucose concentrations after a short period of fasting (6 h) was maintained for 5-6 weeks (Fig.4B)," yet Figure 4B only shows data up to 5 weeks.

In the new version of the manuscript, former Figure 4B has been replaced by current Figure 5B, which shows short-term (6 h) fasting glucose levels in both male and female mice, in response to the AAV-TuD therapy. In this new panel, it can be clearly appreciated that TuD significantly reduced fasting glucose levels for up to 7 weeks after rAAV administration. Accordingly, we have modified the text as follows:

"The decrease in blood glucose concentrations after a short period of fasting (6 h) was maintained for up to 7 weeks…."

Thus far, we have only run one study for longer than seven weeks, which is the 3 month-long study presented also in this manuscript. In that study, we found a trend (P=0.06) towards significantly reduced fasting glucose levels, in the animals receiving the AAV-TuD therapy, as late as 9 weeks following rAAV administration (NC, 142.6±4.4 vs. TuD, 128.7±5.5 mg/dl; data not shown in the manuscript). Unfortunately, it is not possible for us to precisely determine how long the reductions in basal glucose levels can be maintained at this point. However, in said study, a pyruvate tolerance test was run 3 months after rAAV administration (shown in Figure 5D in the

new version of the manuscript). Although at that point fasting glucose levels had already returned to baseline (NC, 141.1±5.7 vs. TuD, 143.8±6.5 mg/dl), the glucose response to a pyruvate overload was still significantly reduced in the animals treated with the TuD; demonstrating that the effect on hepatic glucose out was still present. This observation is presented in the results section as follows:

"Reduced glucose levels following a pyruvate tolerance test were observed up to 3 months after the rAAV administration (Fig.5D; Supplementary Fig.10E), despite basal glucose levels had already returned to baseline, demonstrating that effects on hepatic glucose output were still present at that point."

- Figure 5G: Missing label for the bar graph groups.

This mistake has now been amended. Please note that former Figure 5G is new Figure 6F in the revised version of the manuscript.

- Data in Figure 7 are interesting. Not in the scope of this paper, but it would be interesting to see 1) the mitochondrial experiments shown in Figure 6 completed on the in vivo STZ and DIO tissues generated in Figure 7 to elucidate a potential mechanism, and 2) small RNA sequencing and bulk RNA sequencing of the liver tissues of these mice to assess potential adaptions in response to the TuD inhibitor.

Although interesting studies, we agree with the reviewer that duplicating all the experiments conducted in chow-fed animals also in STZ-treated and DIO mice would require extensive work and would significantly increase the complexity of the study. In turn, we dedicated our efforts to complement our findings in chow-fed animals. Thus, we have now studied oxygen consumption rate in mitochondrial extracts from livers with repression of the *miR-379/miR-410* cluster. In

agreement with our previous observations on enhanced mitochondrial connectivity, expression of the TuD inhibitor was associated with a remarkable elevation in oxygen consumption rate in conditions of either basal respiration, ADP-induced respiration or maximal respiration in response to FCCP incubation (new Figure 7E and 7F). Overall, supporting elevated mitochondrial activity. In addition, we have also provided novel transcriptomics data, depicted in new Supplemental Figure 12. Interestingly, enriched pathway analysis demonstrated that the PI3K-AKT signaling pathway is among the top upregulated pathways in response to TuD expression, supporting our previous observations of enhanced PI3K-mTOR activity in the liver of the animals with repression of the *miR-379/miR-410* cluster.

- Line 411: This sentence is misleading because only a few microRNAs were tested, not the whole cluster and not all other tissues were evaluated. Would suggest removing it.

Following the reviewer's recommendation, we have now removed this sentence from the text. However, we must make clear that we are completely convinced of the liver specificity of our approach. In the original version of our manuscript, we showed no effects on the target microRNAs in white adipose tissue, skeletal muscle and heart. Now we have complemented these findings by showing no effects in brown adipose tissue or central nervous system (hypothalamus), these data are now presented in novel Figure 5C and 5D, as well as novel Supplementary Figure 6B. We understand we have not studied every single tissue/organ in the organism, but we feel that we have studied a high enough number of relevant metabolic tissues to back up our claim. In regards to the number of microRNAs tested, we have not studied the levels of expression of every single microRNA from the cluster because that would have increased the cost of the analysis immensely, but we feel that showing effects on a representative number of these microRNAs (*miR-379*, *miR-541*, *miR-382*, *miR-134*, *miR-409*) as well as the host transcripts *lncMGC* and *Mirg*, is sufficient to give an impression on the general response of the cluster.

- Line 428 is also hyperbolic in nature.

The discussion has been extensively modified in the new version of the manuscript, among the many changes we have removed this sentence.

- Line 532: Clarify the ns for each luciferase assay experiment ("at least 4 times" is vague).

The sentence has been corrected and we now specify that all luciferase experiments were repeated 4 times, as reflected in the respective panels of new Supplemental Figure 5.

- **Methods section should include a section about the Phenomaster experiments.**

As noted above, we have now included a description of the Phenomaster experiments in the supplemental methods section.

- STZ mice were assessed for 9 weeks, DIO mice were assessed for 5 weeks. Why were these time courses not consistent?

With respect, as already described in the methods:

"To study the effects of the TuD in a model of type I diabetes, diabetes was induced by STZ treatment as above and 4 weeks later the animals were administered the rAAV vectors. **The study was terminated 5 weeks after rAAV administration** and insulin therapy was not provided at any time. To study the effects of the TuD in the context of diet-induced obesity, 6 week-old mice were fed a high-fat, high-sucrose (HFHS) diet (45% kcal coming from fat and 20% from sucrose;

D12451; Research Diets, Inc.) for 6 weeks before **rAAV administration. Then, they were maintained on this diet for 5 extra weeks until the end of the study.**

The length of the TuD therapy was exactly the same in both studies (5 weeks) and, therefore, directly comparable. If the reviewer is intrigued by why we did not closely follow water intake and blood glucose levels on the animals of the DIO study during the weeks preceding rAAV administration, as we did on the STZ-treated animals, the response to that is simple: since insulin supporting therapy was not provided in the latter study in order to avoid potential confounder effects, the veterinary authorities of our animal facility requested that the animals should be closely monitored from the moment STZ was administered, so we could identify animals in potential need for such therapy in a timely manner. Fortunately, the physical condition of all animals was sufficiently good to avoid the supporting therapy. In contrast, the requirements for the evaluation of the physical condition of the animals on the high-fat, high-sucrose diet were not so strict and we did not conduct such a close monitorization in this experiment. However, if the reviewer is particularly interested in some metabolic parameters before the rAAV administration (such as 6-fasting glucose levels), we can disclose that information: Chow + NC, 144.2±4; chow + TuD, 145.2±3.7; HFHS + NC, 183.2±4.4; HFS + TuD, 186±7.2; the fasting glucose levels were 30% higher in the HFHS-fed group as compared to the regular chow-fed animals ($P<0.001$)

- One or two tailed t-tests in statistics section? The statistical methods section could use additional detail and clarification.

Two-tailed t-tests were used when comparing two independent groups. This information is now added to the description of the statistical analysis. We have now also included information regarding the correlation analysis performed on the human data, which was omitted by mistake in the first version of the manuscript. In addition, we have also provided a reference for the calculations of the Matsuda's composite index, we specified that the nonlinear curve fitting

calculation shown in novel Supplementary Figure 3A-C was performed using OriginPro 2024 software and we also described that the analysis of the body mass-dependent data shown in novel Supplementary Figure 8 was conducted using CalR. We hope that the reviewer will find the updated description of the statistical analysis satisfactory.

- Scale bars should be provided on images in figures 3D, 3E, 6B and 6C.

We agree and have now appended the scale bars to the images.

- Protein molecular weights should be provided on figures 5A-C and E-G.

We concur and have now added the molecular weight marks to all immunoblot images presented in the manuscript.

- More details should be provided on the gene expression assay section in the methods.

We have expanded the RNA analysis section of the methods by specifying that the pieces of tissue used for the analysis were snap-frozen upon collection and then homogenized in TRIzol prior to the isolation of the RNA. This procedure minimizes the risk of RNA degradation. Occasionally (although not for all the samples and experiments and that is why this is not mentioned in the description) RNA integrity was measured in a Bioanalyzer, obtaining RIN within the 8-10 range, and thus confirming the high quality of the RNA we were working with. For space constraint we have not expanded on the protocols followed for RNA isolation and reverse transcription, but we have indicated now that all steps were conducted according to manufacturer's recommendations of the kits we used. We have also now specified in the description that a QuantStudio™ 7 Flex Real-Time PCR System was employed to perform the qRT-PCR measurements. Finally, we now indicate that each sample was run in triplicate. We

hope that the reviewer will find the updated description of the RNA analysis satisfactory.

REVIEWER COMMENTS

Reviewer #2 (Remarks to the Author):

I would like to express my appreciation for the revised manuscript and to thank the authors for the extensive additional work, including the FGF21 knockout (KO) experiments, RNA seq, proteomics, and the revisions to the explanatory text. However, I still have a few concerns.

We are grateful for the reviewer's recognition of our effort to improve the manuscript.

The authors report mild but significant increases in hepatic transaminases and fat accumulation following TuD treatment in the diet induced obesity (DIO) model. While they attribute discrepancies with previous reports to differences in the KO model, this explanation seems insufficient. The manuscript would be strengthened by additional discussion to more fully address this point.

We acknowledge that our novel findings of increased hepatic fat accumulation in the diet-induced obese animals expressing the TuD has not been adequately discussed. We have now corrected that by including a segment in the discussion dealing with those findings as follows:

"Despite the enhanced lipoprotein uptake, no increased hepatic lipid deposition was observed in animals fed regular chow, likely because of the concomitantly elevated mitochondrial oxidation. However, hepatic triglyceride content was slightly elevated in our model of diet-induced obesity, suggesting that the increased mitochondrial function is not sufficient to overcome the augmented hepatic lipid flux in this model. In contrast, previous studies have found amelioration of hepatic steatosis in high-fat diet fed mice with constitutive deletion of a fragment of the cluster[20]. This discrepancy might be explained by the dramatic reduction in adipose depot mass observed in the constitutive model (likely decreasing the fatty acid influx from the adipose tissue); and by presumably enhanced fatty acid consumption in the skeletal muscle, as the constitutive model presents marked muscular hypertrophy[17] and inhibition of this cluster in the muscle has demonstrated to locally stimulate mitochondrial function[37]."

In addition, some sentences remain rather long. I kindly recommend making certain descriptions more concise, while elaborating a little more carefully on the key arguments where appropriate.

Following the reviewer's suggestion, we have now reduced the word count in the results and discussion section on more than 600 words, hoping it will improve readability.

Reviewer #3 (Remarks to the Author):

Gil-Lozano et al. completed additional work to address considerable comments from both reviewers in the manuscript titled "Combinatorial targeting 1 of the miR-379/miR-410 cluster normalizes glucose and lipid homeostasis in male models of diabetes and obesity." The authors clarified points that previously lacked detail in the manuscript, such as animal experiment design and potential toxicity of the TuD by completing additional imaging for liver inflammation in treated and control mice. In response to questions regarding sex as a biological factor, in the revised manuscript the authors included data in female mice for the TuD chow studies and clarified sample sex in the clinical dataset. Additionally, it was clarified that only cells with distinctive hepatocyte morphology were considered in these analyses and the authors completed studies to parse apart the role of glucocorticoids on the hepatocyte fraction relative to the non-parenchymal cell fraction. These efforts improved general data consistency throughout the manuscript.

We are thankful for the reviewer's appreciation of our work to improve the manuscript.

Major points:

1. The premise of this work relies upon the assertion that miR-379 and miR-541 are upregulated in the liver of clinical population members with an obese phenotype. During the initial review, information regarding the sex of the clinical study participants was requested, as this information was previously not included in the first draft of the manuscript. Sex as a biological factor is important (SAGER guidelines) and is known to

influence energy homeostasis, body weight, and miRNA expression (Mauvais-Jarvis 2015, Link et al. 2017, Sharma & Eghbali 2014). In the revisions, the authors did provide information on human donor sample sex in Supplemental Table 1. This data revealed that 3 non-obese males and 2 non-obese females were included in the control group, and 0 obese males and 11 obese females were included in the obese group- both sexes were used for controls, but only females were used for obese cases in the analysis. As mentioned, sex is known to play a critical role in miRNA expression and key sex differences exist in obesity. There is no discussion of this study design issue in the text and the subsequent analyses (Figure 1) are not balanced if only females are represented in the obese cohort. This represents a significant study design limitation. The authors could address this issue by including obese male cases in the data analysis, or by restricting the analysis to only female participants and clearly stating in the manuscript text and all associated figured that this data only applies to females ("obese human subjects" as currently stated in the current text would need to be changed to "obese female human subjects," for example).

Following the suggestions of the reviewer, we have now studied hepatic levels of expression of two representative miRNAs from the *miR-379/miR-410* cluster, *miR-379* and *miR-541*, in a new larger cohort of subjects including both males and females of non-diabetic lean participants (BMI<30; 14 males, 10 females), and non-diabetic obese individuals (BMI>40; 13 males, 29 females). The results are depicted in new supplemental figures 1B and 1C and they unequivocally show that the upregulation of the miRNAs is not sex dependent but rather an obesity driven effect. These novel findings are also presented in the results section as follows:

"Notably, upregulation of both miRNAs was confirmed in a larger cohort, with no sex differences in expression (Supplementary Fig.1B-C)."

In addition, we have also incorporated a sentence in the discussion stating that:

"…. comparable levels of expression of *miR-379* and *miR-541* were detected between males and females in both lean and obese subjects, indicating lack of sex differences in the hepatic expression of the cluster".

2. The in vivo portion of this study sought to determine if a tough decoy (TuD) against miR-379 and miR-541 could impact metabolic phenotypes in mice. The authors did replicate the initial study to determine TuD efficacy in chow fed male and female mice, which provided new information to the study, including the observation of a significant difference in plasma cholesterol response to TuD therapy in female mice relative to male mice. The authors did not replicate the models of diabetes or prediabetic diet-induced obesity studies in female mice, and revised the study's title accordingly. In the methods section (Line 640), it is stated that "Young male and female C57BL/6J mice (6 weeks of age) were obtained from Charles River Laboratories (Sulzfeld, Germany)." The authors need to clarify that both sexes were only obtained for the chow experiments, and it needs to be made explicitly clear in the manuscript text and figures/figure legends if only male mice were utilized for the given experiment. Ideally, the diabetes experiments should be replicated in female mice. If this is not feasible, the authors should provide additional discussion of this serious limitation of the studies in the manuscript.

As requested by the reviewer, we have now clarified in the methods section that female mice were only used in the chow studies by inserting a sentence stating that:

"Female mice were used in only one study designed to evaluate the sex-dependent effects of hepatocyte-specific repression of the *miR-379/miR-410* cluster in animals fed regular chow, for the rest of the studies only male mice were used."

In addition, we now clearly indicate in the legends whether the figures they refer to, show data from both males and females or only male mice.

We acknowledge that the exclusion of female mice from our experiments with diet-induced obese and STZ-induced diabetic models is a serious limitation of our manuscript, but since we have demonstrated that repression of the cluster promoted comparable metabolic effects in

both male and female mice fed regular chow, we are confident that improvements in glucose and lipid homeostasis are also expected in female models of obesity and diabetes in response to such manipulation. We have now addressed this important issue in the discussion by including a paragraph stating that:

“Finally, considering the relevance of sex as biologic factor for metabolism, we studied the effects of hepatocyte-specific repression of the *miR-379/miR-410* cluster in both males and females fed regular chow. As expected, female mice showed reduced circulating levels of triglycerides and fasting glycaemia, as well as improved glucose clearance and reduced glucose excursions in response to pyruvate and insulin tolerance test, as compared to males[65,66]. Despite the profound sex differences, repression of the cluster promoted comparable effects on these parameters irrespective of sex, denoting no sexual variation in the response to this manipulation. However, we failed to evaluate the effect of sex in our models of diet-induced obesity and STZ-induced diabetes as only males were used. We acknowledge this as an important limitation of our study. Because diabetic female patients respond better to insulin sensitizers than males[67], further studies on the repression of the cluster in female models of diabetes and prediabetes are clearly warranted.”

REVIEWERS' COMMENTS

Reviewer #2 (Remarks to the Author):

I would like to commend the authors for their substantial and rigorous revision of the manuscript. The additional experiments and analyses, including the Fgf21 knockout studies, transcriptomic and proteomic analyses, validation of liver-specific signaling pathways, and mitochondrial functional assays, have significantly strengthened the mechanistic depth and overall rigor of the study. In my view, most of the previous major scientific concerns have now been satisfactorily addressed.

The authors' response to the concerns regarding sex as a biological factor is also appropriate and transparent. The inclusion of a larger validation cohort comprising both male and female human subjects supports the conclusion that hepatic upregulation of miR-379/miR-541 is associated with obesity rather than being primarily sex-dependent. The authors also clearly acknowledge the limitation that the rodent disease models of diabetes and diet-induced obesity were examined only in males.

One remaining issue concerns the framing of safety. In the DIO model, TuD treatment is associated with increased hepatic steatosis and mild but significant elevations in circulating ALT and AST. The additional discussion explaining these findings in relation to hepatic lipid flux and differences between the hepatocyte-specific TuD model and previous constitutive knockout models is helpful and biologically plausible. However, the current safety framing still appears somewhat too strong. In particular, the abstract states that the treatment produced metabolic benefits "with no discernable side effects," and the conclusion refers to an "apparent lack of toxicity." These statements are not fully consistent with the DIO safety data.

We concur and have now removed these statements from the abstract and the conclusion.

I therefore recommend that the authors revise the abstract, summary/conclusion, and relevant discussion to explicitly acknowledge the mild hepatic safety signal observed

in the DIO model. The manuscript should make clear that no overt toxicity was observed under chow-fed conditions, but that increased hepatic fat accumulation and transaminase elevations were detected under diet-induced obesity. The potential implications for translation to obese, insulin-resistant, or fatty liver disease contexts should also be discussed more carefully.

The increased hepatic lipid accumulation observed in our diet-induced obese model following the repression of the cluster was already addressed in the results and in the discussion. However, recognizing the implications of this finding, we have now added a new statement in the discussion saying:

"Considering the increased lipid accumulation in our obese mice, the safety of repressing the cluster in other models of obesity, insulin resistance or MASLD deserves further evaluation."

In addition, there appear to be inconsistencies in the supplementary figure numbering. The STZ-related supplementary data are cited in the main text as Supplementary Fig. 16, whereas the supplementary material appears to present these data as Supplementary Fig. 17. Similarly, the DIO-related data are cited as Supplementary Fig. 17, while the supplementary material appears to present them as Supplementary Fig. 18. These cross-references should be corrected before publication.

We thank the reviewer for noticing these mistakes which have now been amended.

<u>Reviewer #3 (Remarks to the Author):</u>

I appreciate the additional work the authors completed to ensure sex differences were accounted for in the human portion of this manuscript. The new insights into miR-379 and miR-541 in both male and female obese and non-obese study participants strengthens the manuscript.

I still have considerable concerns about female mice not being included in the in vivo models of diet-induced obesity and STZ-induced diabetic models. I acknowledge that the authors provided additional clarifications within the manuscript text and discussion regarding the limitations of not studying sex differences in these models. However, I strongly disagree with the notion that "we are confident that improvements in glucose and lipid homeostasis are also expected in female models of obesity and diabetes in response to such manipulation." To be confident in this outcome, the work must be completed, especially since there are 1) profound metabolic differences in the STZ-induced and diet-induced obesity mouse models compared to mice fed standard rodent chow and 2) marked differences in metabolism between the sexes. For publication, I would strongly recommend that these experiments be completed, as sex as a biological variable is of preclinical significance.

We are glad that the reviewer finds the new human data compelling, and we regret our statement on the potential effects of our TuD in female models of obesity and diabetes as it was overstated. However, we would like to remind the reviewer that such claim was included in the rebuttal but not in the text of the manuscript where, after acknowledging the relevance of sex as a biological factor for metabolism, we more cautiously stated that "….we failed to evaluate the effect of sex in our models of diet-induced obesity and STZ-induced diabetes as only males were used. We acknowledge this as an important limitation of our study. Because diabetic female patients respond better to insulin sensitizers than males[67], further studies on the repression of the cluster in female models of diabetes and prediabetes are clearly warranted".
